# In Silico Design and Validation of a Novel HPPD‐Inhibiting Herbicide Candidate Based on Benzofuran and Arylthioacetic Acid Scaffolds

**DOI:** 10.1002/cbdv.202503221

**Published:** 2025-11-17

**Authors:** Luiz R. Capucho, Elaine F. F. Cunha, Matheus P. Freitas

**Affiliations:** ^1^ Department of Chemistry Institute of Natural Sciences Federal University of Lavras Lavras Brazil

**Keywords:** MIA‐QSAR, molecular docking, molecular dynamics, HPPD, benzofuran

## Abstract

Inhibition of 4‐hydroxyphenylpyruvate dioxygenase (HPPD) is a well‐established strategy for weed control, yet the emergence of resistance underscores the need for more potent inhibitors. In this study, datasets of benzofuran analogues and arylthioacetic acid–derived triketones were analyzed using multivariate image analysis of quantitative structure–activity relationships (MIA‐QSARs) to guide the identification of promising candidates. Molecular docking was conducted on HPPD, including Co(II) substitution to explore metal–ligand interactions, and molecular dynamics simulations, parametrized with MCPB.py, evaluated the stability and interaction patterns of the complexes. Fourteen candidates were proposed, six of which exhibited higher predicted activity and improved performance relative to mesotrione. Among them, candidate P1 emerged as the most promising, reproducing key interactions of mesotrione while displaying lower binding energy and stable convergence during dynamics. These results demonstrate P1 as a novel HPPD inhibitor and highlight the utility of combining MIA‐QSAR, docking, and MCPB.py–parametrized dynamics in rational metalloenzyme inhibitor design.

## Graphical abstract

A benzofuran‐triketone hybrid emerged as the most promising 4‐hydroxyphenylpyruvate inhibitor, showing the highest predicted activity and strongest binding compared to the best experimental compound. Free computational tools guided candidate selection, predicted interactions with the enzyme, and confirmed stable binding. These findings highlight the compound's potential as a novel herbicide, comparable to the commercial standard mesotrione, and demonstrate the value of integrated computational approaches for designing effective enzyme inhibitors.

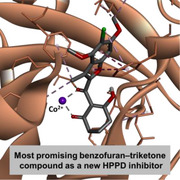

## 1 Introduction

The hydroxyphenylpyruvate dioxygenase (HPPD) active site is a key target in the search for safe and selective herbicides [1, 2, 3, 4]. Triketone compounds, designed to structurally compete with the natural substrate hydroxyphenylpyruvate, have been developed following the observation of the allelopathic effects of leptospermone on weeds surrounding the tree *Melaleuca citrina*, with sulcotrione being the first commercially recognized and patented compound in 1991 [5, 6, 7].

From a biological perspective, HPPD catalyzes the reaction that produces homogentisate [8]. This compound, as a precursor in the biosynthesis of plastoquinone and α‐tocopherol, is essential for plants because it participates directly in carotenoid synthesis, reactive oxygen species scavenging, and electron transport in the photosynthetic chain [1, 7, 9, 10]. Consequently, its depletion causes severe plant damage, primarily characterized by chlorophyll destruction, which has led to the classification of HPPD inhibitors as bleaching herbicides [3, 5, 11, 12].

However, the growing resistance of weeds to commercial compounds has driven research toward the development of new structures capable of inhibiting HPPD and, consequently, homogentisate synthesis [13]. Among the previously susceptible weed species in which HPPD inhibitors were once effective, seven have already been reported to have developed resistance [14]. This finding justifies the design of new, optimized molecules to overcome current limitations, such as the need for higher application rates and the use of undesirable combinations. Thus, the use of quantitative structure–activity relationship (QSAR) methods emerges as a promising strategy to guide the design of new candidate molecules, which, within a defined set of analogous and diverse structures, may exhibit improved inhibitory activity by exploring combinations of substituents reported in the literature [15].

In this context, a multivariate image analysis of QSAR (MIA‐QSAR) approach is particularly attractive due to the ease of descriptor generation and its low computational cost. In addition to its simplicity, enhancements that account for Van der Waals radii and color diversity in atom representations allow periodic properties to be considered for each element type [16], providing a more robust analysis than older binary descriptor methods, which could not incorporate additional information [17, 18]. Beyond model calibration, MIA plots complement this approach by visually representing the statistical results, thereby facilitating the interpretation of group performance and supporting the design of new molecules with enhanced activity [18].

**FIGURE 1:**
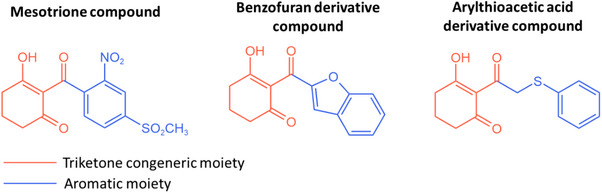
Analogous triketone hybrid compounds from standard compounds and proposed on the used dataset.

The requirements for an effective HPPD inhibitor are as previously described: a conserved chelation region, which mimics the α‐keto acid substrate moiety to enable competitive inhibition, and a binding site on an aromatic moiety, which exhibits higher variability among the set of possible interactions [19, 20]. Among all these possible compounds, different triketone HPPD hybrids are able to be combined for QSAR‐based optimization, due to their ketonic congeneric moiety, as shown in Figure 1.

Despite the stacking interactions promoted by the aromatic ligand moieties, electronic effects primarily govern the enhancement of these compounds’ bioactivity. The introduction of electron‐withdrawing groups on the aromatic ring is frequently recommended, as they increase the α‐carbonyl p*K*a, thereby promoting the formation of the enolic tautomer, which represents the active inhibitory form [21, 22, 23]. In addition, such substituents reduce the electron density of the aromatic rings, thereby facilitating stronger π–π interactions with phenylalanine residues [20, 24, 25].

Understanding these key interactions has encouraged researchers to explore the influence of different aromatic ring systems, aiming to optimize π–π stacking interactions among diverse structural frameworks. Given the well‐established bioactive properties of benzofuran and arylthioacetic acid derivatives, some studies introduced structural modifications at the triketone moiety to identify new hybrid triketone herbicides [4, 26, 27, 28, 29]. As a result, several active analog inhibitors were discovered *in vitro*, providing a valuable foundation for subsequent biological and computational studies.

In the present study, both compound sets were analyzed using the MIA‐QSAR approach, and new candidate molecules were proposed based on the obtained results. Rigid molecular docking was then performed to investigate potential enzyme–ligand interactions, followed by molecular dynamics simulations to validate the stability of these formed complexes.

## 2 Methods

### 2.1 MIA‐QSAR Procedure

Two datasets comprising 59 congeners derived from benzofuran and arylthioacetic acid [4, 28] were merged and analyzed using the MIA‐QSAR method. In the Y block, the inhibitory activity values, measured by in vitro assays in the original works, were converted to p*K*_i_ values (–log *K*_i_) [30, 31, 32] (Table 1). The selection of both scaffold molecules was guided by specific methodological requirements. First, the biological assay procedures reported in the original studies were comparable, and the reference compound exhibited similar activity values across both datasets, ensuring consistency in the bioactivity measurements. Second, the previously demonstrated inhibitory potential of these compounds confirmed their feasibility as biologically active candidates. Finally, the presence of a congeneric moiety within the molecular structures was considered essential for MIA‐QSAR modeling, allowing proper alignment and descriptor correlation.

**TABLE 1:** Triketone derivatives used in the calibration step of quantitative structure–activity relationship (QSAR) modeling and their corresponding p*K*_i_ values (*K*_i_ in mol/L).

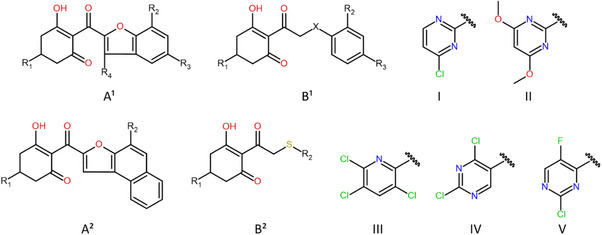						

Cpd.	R^1^	R^2^	R^3^	R^4^	X	p*K*_i_
**A^1^1**	H	H	H	H	—	6.5952
**A^1^2**	di‐CH_3_	H	H	H	—	6.2411
**A^1^3**	H	H	H	CH_3_	—	6.9547
**A^1^4**	di‐CH_3_	H	H	CH_3_	—	6.9101
**A^1^5**	H	OCH_3_	H	H	—	6.2790
**A^1^6**	di‐CH_3_	OCH_3_	H	H	—	6.1959
**A^1^7**	H	H	Cl	H	—	7.1308
**A^1^8**	di‐CH_3_	H	Cl	H	—	6.4572
**A^1^9**	H	H	NO_2_	H	—	7.0223
**A^1^10**	di‐CH_3_	H	NO_2_	H	—	6.8327
**A^1^11**	H	Cl	Cl	H	—	6.9914
**A^1^12**	di‐CH_3_	Cl	Cl	H	—	6.9547
**A^1^13**	H	NO_2_	H	CH_3_	—	6.6253
**A^1^14**	di‐CH_3_	NO_2_	H	CH_3_	—	6.1772
**A^2^15**	H	H	—	—	—	6.0670
**A^2^16**	H	NO_2_	—	—	—	5.8386
**A^2^17**	di‐CH_3_	NO_2_	—	—	—	5.6778
**B^1^1**	H	H	CH_3_	—	SO_2_	5.3736
**B^1^2**	H	H	H	—	SO_2_	5.5358
**B^1^3**	H	H	CH_3_	—	S	5.7762
**B^1^4**	di‐CH_3_	H	CH_3_	—	S	5.5405
**B^1^5**	H	H	Cl	—	S	7.2840
**B^1^6**	di‐CH_3_	H	Cl	—	S	7.0506
**B^1^7**	H	H	NO_2_	—	S	6.9318
**B^1^8**	di‐CH_3_	H	NO_2_	—	S	6.6968
**B^1^9**	H	NO_2_	H	—	S	7.6778
**B^1^10**	di‐CH_3_	NO_2_	H	—	S	7.1805
**B^1^11**	H	NO_2_	NO_2_	—	S	6.3737
**B^1^12**	di‐CH_3_	NO_2_	NO_2_	—	S	6.5157
**B^1^13**	H	CF_3_	NO_2_	—	S	6.4461
**B^1^14**	di‐CH_3_	CF_3_	NO_2_	—	S	6.5346
**B^1^15**	H	NO_2_	Cl	—	S	7.1739
**B^1^16**	di‐CH_3_	NO_2_	Cl	—	S	7.1079
**B^1^17**	H	NO_2_	CF_3_	—	S	6.9431
**B^1^18**	di‐CH_3_	NO_2_	CF_3_	—	S	6.8041
**B^2^19**	H	I	—	—	—	6.8125
**B^2^20**	di‐CH_3_	I	—	—	—	6.5622
**B^2^21**	H	II	—	—	—	7.1549
**B^2^22**	di‐CH_3_	II	—	—	—	7.0315
**B^2^23**	H	III	—	—	—	7.6021
**B^2^24**	di‐CH_3_	III	—	—	—	7.2441
**B^2^25**	H	IV	—	—	—	7.0757
**B^2^26**	di‐CH_3_	IV	—	—	—	7.0269
**B^2^27**	H	V	—	—	—	7.3468
**B^2^28**	di‐CH_3_	V	—	—	—	7.3010
**Mesotrione**	—	—	—	—	—	7.6990

The chemical structures for the X‐block (descriptors) were sketched in GaussView, ensuring consistent alignment across all compounds [33]. Shadowing effects were disabled, and the structures were saved in MOL2 format. Pictorial representations of each compound were obtained as pixel values (432 × 300 pixels), ranging from 0 to 765 on the RGB color scale. These values were then modified by incorporating periodic properties such as electronegativity (ε), Van der Waals radius (r_VdW_), and the ratio r_VdW_/ε, and paired with inhibitory activities using Chemoface software [34]. The correlation between descriptors and responses was modeled using partial least squares (PLS) regression, and William´s plots were employed for outlier identification. Parameters from cross‐validation and Y‐randomization, such as *r* ^2^, *q* ^2^, ^c^ *r* ^2^_p_, and their respective root mean square error (RMSE) values, were used to assess model quality [35, 36, 37, 38].

External validation was performed using a bootstrapping methodology, in which 25% of the samples (totaling 14 samples for each cycle) were randomly separated in each of 10 modeling iterations, in which the 14 removed samples, based on which iteration, can be found in the Supporting Information. Validation parameters at this stage included *r* ^2^_pred_, Avg. *r* ^2^_m_, Δ*r* ^2^_m_ (Roy's parameters) [27], and the concordance correlation coefficient (CCC) [39, 40]. VIP and **b**‐plots, related to variance and PLS regression coefficients, were generated and used to propose new potential structures, whose activities were predicted with the previously developed models [18]. Finally, a synthetic route, inspired by the original dataset article, was proposed for the most active molecule obtained to illustrate the feasibility of its synthesis.

### 2.2 Molecular Docking Protocol

To evaluate the correlation between the inhibitory activity of the proposed molecules and their interaction energies within the enzyme cavity, molecular docking was performed with the HPPD enzyme from *Arabidopsis thaliana* (PDB ID: 5YWG; EC 1.13.11.27) [41]. The complex contained a cobalt (II) cofactor and the ligand mesotrione, which was also used in the QSAR dataset as the standard compound. For the removal of water molecules and ligand separation, Discovery Studio software was employed [42]. The ligand, saved in MOL2 format, was processed by adding hydrogens, calculating partial charges with the Gasteiger force field, and correcting atom hybridization and bond types.

The redocking procedure, as protocol validation, was carried out with AutoDockTools [43]. The enzyme (apo form, in PDB format) had polar hydrogens and partial charges added using the Gasteiger method. Cofactor parameters were set according to the user manual, and the file was saved in PDBQT format. Using a text editor, the charge of cobalt was manually adjusted to +2 to reflect its oxidation state.

The ligand, also converted to PDBQT format after identifying active torsions, was docked with a Genetic Algorithm search method, using a rigid protein structure. The binding cavity was defined based on the crystallized ligand position, with the grid box centered at coordinates (24.434, ‐7.429, ‐29.328) for the x, y, and z axes, respectively. To encompass the entire cavity region, 40 grid points were used in each dimension with a grid spacing of 0.375 Å.

The docking parameters included an initial population of 150 individuals, a mutation rate of 0.02, a crossover rate of 0.8, and a maximum of 27,000 generations. A total of 100 independent runs were conducted. The quality of the docking poses—and thus the reliability of the protocol—was evaluated using the Docking Accuracy (DA) equation, based on the root mean square deviation (RMSD) relative to the crystallized reference structure [44]. This analysis identifies poses with the smallest deviations from the experimental conformation, with DA values closer to 1 indicating better performance. Equation 1 expresses this relationship, where f corresponds to the fraction of poses within a defined RMSD range, and l and h denote the lower and upper RMSD limits, respectively (l < h) [44]. The adopted l and h values were 2 and 3 Å, consistent with those commonly used in the literature [45].

$$DA=f_{l}+0.5\;\left(f_{l}-f_{h}\right)$$

For the proposed ligands, the same preparation and docking protocol was applied, except for an additional structural minimization performed using the DREIDING force field in Discovery Studio software.

### 2.3 Molecular Dynamics

For molecular dynamics simulations, the MCPB.py tool in AmberTools24 was employed [46, 47, 48]. Based on the optimized mesotrione–HPPD complex, a hybrid approach was used, in which only the amino acid residues directly bound to the metal ion were included [49]. For the bonded‐system parametrization in Gaussian16, the cobalt (II) ion was coordinated with histidine and glutamate residues, and the standardized ligand was used to preserve octahedral geometry [50]. Calculations were performed at the B3LYP/6‐31G level, after which ligand–metal bonds were removed and the system was converted to GROMACS parameters [51].

Four simulation steps were performed in GROMACS: (i) energy minimization using the steepest descent method; (ii) NVT equilibration (–DPOSRES, 100 ps, dt = 2 fs) to stabilize temperature with positional restraints; (iii) NPT equilibration, under the same conditions, to adjust system density and pressure; and (iv) production dynamics without restraints, consisting of 100 ns simulated over 50 000 000 steps (dt = 2 fs). From the resulting trajectories, the RMSD of the ligand within the enzyme cavity was calculated, as well as root mean square fluctuation (RMSF) values for all residues. B‐factors were also used as a pictographic tool to visualize structural flexibility in both the enzyme and ligands [52].

### 2.4 Physicochemical Properties and ADMET Analysis

For the human safety evaluation, aimed at ensuring low health risks of the proposed compounds, ADMET predictions were performed using the ADMETlab webserver [53]. The two most promising proposed molecules, in comparison with mesotrione, were assessed based on commonly employed ADMET parameters reported in previous studies: solubility (log *S*), absorption (Caco‐2), distribution (BBB and PPB), and toxicity, including hepatotoxicity, skin sensitization, and carcinogenicity [2, 54, 55, 56, 57]. Finally, the log *P* value for each proposed molecule was predicted with the Molinspiration web server, a reliable predictor for this parameter [58].

## 3 Results and Discussions

### 3.1 MIA‐QSAR Procedure

The MIA‐QSAR method applied in this study is advantageous because it reduces both operational and computational costs while providing valuable interpretative insights through MIA plots. The resulting models typically exhibit high accuracy and predictive power, further supported by docking analyses that confirm the correlation between inhibitory activity and the binding energies of the predicted poses within the biological target.

The dataset was monitored to identify potential outliers. Of the total samples, 59 were deemed suitable for analysis, as they had defined inhibitory activity values (*K*_i_). The most suitable models were defined as those showing the lowest RMSE in the cross‐validation procedure. Within this subset, two samples (B^1^9 and B^1^10) showed significant influence in the Williams plot, as evaluated by the threshold of 2.5 for *p* in the studentized residual analysis (see Supporting Information). The decision to remove these two samples was based on improvements in the statistical parameters adopted and on the increased information content in both the X and Y blocks.

**FIGURE 2:**
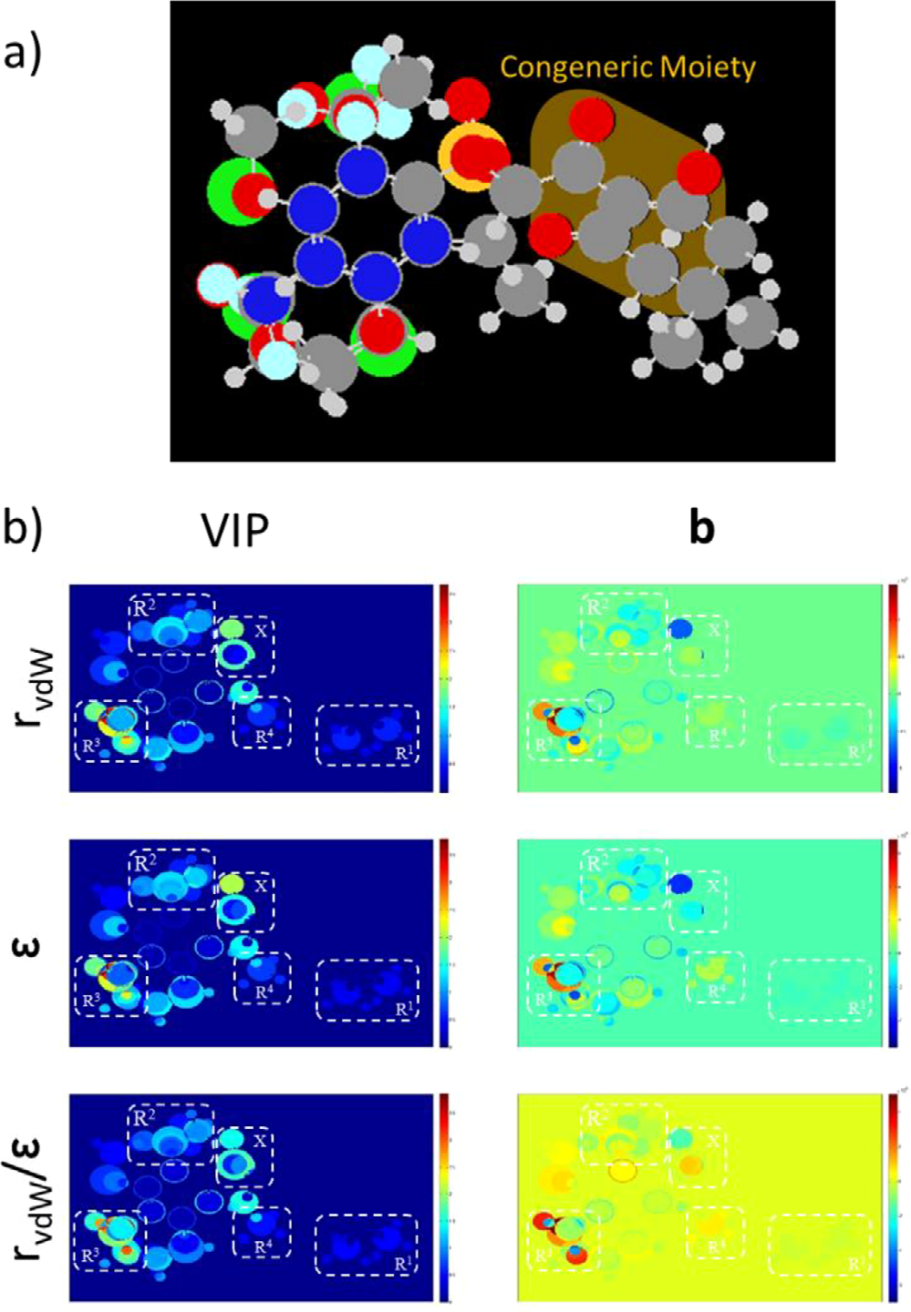
Multivariate image analysis (MIA) plots of the best‐performing model (Model 7).

In the developed models, the PLS method demonstrated consistent performance across the three variables used as descriptors for block X. Performance evaluation included the goodness of fit (*r* ^2^), the correlation between dependent variables (*r* ^2^_y‐rand_ and ^c^ *r* ^2^_p_), the predictive ability for the calibration set (*q* ^2^), and the accuracy in predicting the behavior of external samples (*r* ^2^_pred_, *r* ^2^_m_, CCC). Acceptable values were obtained for all three adopted properties, as summarized in Table 2. Among the models generated for each periodic property, the best‐performing model was defined as the one with the highest average values above 0.5. Accordingly, Model 7 (from 10 bootstrap runs) was identified as the most suitable. Its MIA plots, shown in Figure 2, allow a clear distinction of the groups through visualization of the superimposed sample images.

**TABLE 2:** Statistical parameters to multivariate image analysis of quantitative structure–activity relationship (MIA‐QSAR) validation obtained from the bootstrapping procedure.

Parameter	r_vdW_	ε	r_vdW_/ε	Average	Std. Dev.	Cut‐off
**PLS comp**.	6.60	5.30	7.80	6.5667	1.2503	—
**RMSEC**	0.1725	0.1802	0.1681	0.1736	0.0061	—
***r* ^2^**	0.9022	0.8932	0.9057	0.9004	0.0064	≥ 0.6 [38, 59 ]
**RMSEy‐rand**	0.3772	0.3886	0.3695	0.3784	0.0096	—
***r* ^2^_y‐rand_**	0.5268	0.5017	0.5487	0.5258	0.0235	[*r* ^2^_y‐rand_ << *r* ^2^] [38]
**^c^ *r* ^2^_p_**	0.5788	0.5908	0.5679	0.5792	0.0115	≥ 0.5 [35]
**RMSECV**	0.3663	0.3634	0.3594	0.3630	0.0035	—
***q* ^2^**	0.5819	0.5868	0.5939	0.5875	0.0060	≥ 0.5 [38, 39, 59]
**RMSEP**	0.2555	0.2399	0.2731	0.2561	0.0166	—
***r* ^2^_pred_**	0.7315	0.7676	0.7098	0.7363	0.0292	≥ 0.5 [39]
**Avg. *r* ^2^_m_**	0.6006	0.6573	0.5697	0.6092	0.0445	≥ 0.5 [39, 40]
**Δ*r* ^2^_m_**	0.1384	0.1196	0.1279	0.1286	0.0094	< 0.2
**CCC**	0.8326	0.8600	0.8157	0.8361	0.0223	≥ 0.8 [60]

The overall analysis of the MIA plots revealed significant variance and strong correlations between substituents and inhibitory activity, particularly at the R_3_ position. The –OCH_3_ and –Cl substituents were identified as the most effective contributors to increased activity, consistent with previous reports highlighting the superior performance of electron‐withdrawing groups directly attached to the aromatic ring adjacent to the triketone carbonyl. In contrast, alkyl substitutions at the R_1_ position produced undesirable effects, as also noted in earlier studies, due to an unfavorable increase in steric hindrance. Other positions showed beneficial, though less pronounced, effects. Consequently, –Cl and –F at R_2_ and –CH_3_ at R_4_ were also considered in the design of new structures, as summarized in Table 3.

**TABLE 3:** Proposed structures based on the multivariate image analysis (MIA) plot from quantitative structure–activity relationship (QSAR) analysis.

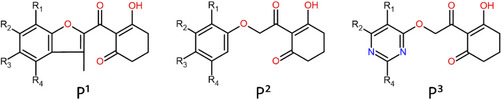									

Cpd.	R_1_	R_2_	R_3_	R_4_	r_vdW_	ε	r_vdW_/ε	p*K*i	Std. Dev.
P^1^1	H	OCH_3_	Cl	OCH_3_	8.0549	8.1459	8.0104	8.0704	0.0691
P^1^2	Cl	OCH_3_	Cl	OCH_3_	8.0167	8.2153	7.9353	8.0558	0.1440
P^2^3	H	OCH_3_	CF_3_	OCH_3_	7.4897	7.6888	7.2750	7.4845	0.2070
P^2^4	F	OCH_3_	CF_3_	OCH_3_	7.5984	7.9110	7.2831	7.5975	0.3139
P^2^5	H	Cl	CF_3_	Cl	7.7436	7.8505	7.5295	7.7079	0.1635
P^2^6	H	OCH_3_	Cl	OCH_3_	7.7334	7.7879	7.5855	7.7023	0.1047
P^2^7	Cl	OCH_3_	Cl	OCH_3_	7.6952	7.8573	7.5104	7.6876	0.1736
P^2^8	H	Cl	Cl	Cl	7.9887	7.9510	7.8411	7.9269	0.0767
P^2^9	Cl	Cl	Cl	Cl	7.9505	8.0204	7.7660	7.9123	0.1314
P^3^10	H	OCH_3_	—	OCH_3_	7.4896	7.4049	7.5171	7.4706	0.0585
P^3^11	F	OCH_3_	—	OCH_3_	7.5983	7.6271	7.5253	7.5836	0.0525
P^3^12	Cl	OCH_3_	—	OCH_3_	7.4514	7.4743	7.4420	7.4559	0.0166
P^3^13	H	Cl	—	Cl	7.7422	7.5662	7.7701	7.6928	0.1105
P^3^14	Cl	Cl	—	Cl	7.7040	7.6356	7.6950	7.6782	0.0372

**FIGURE 3:**
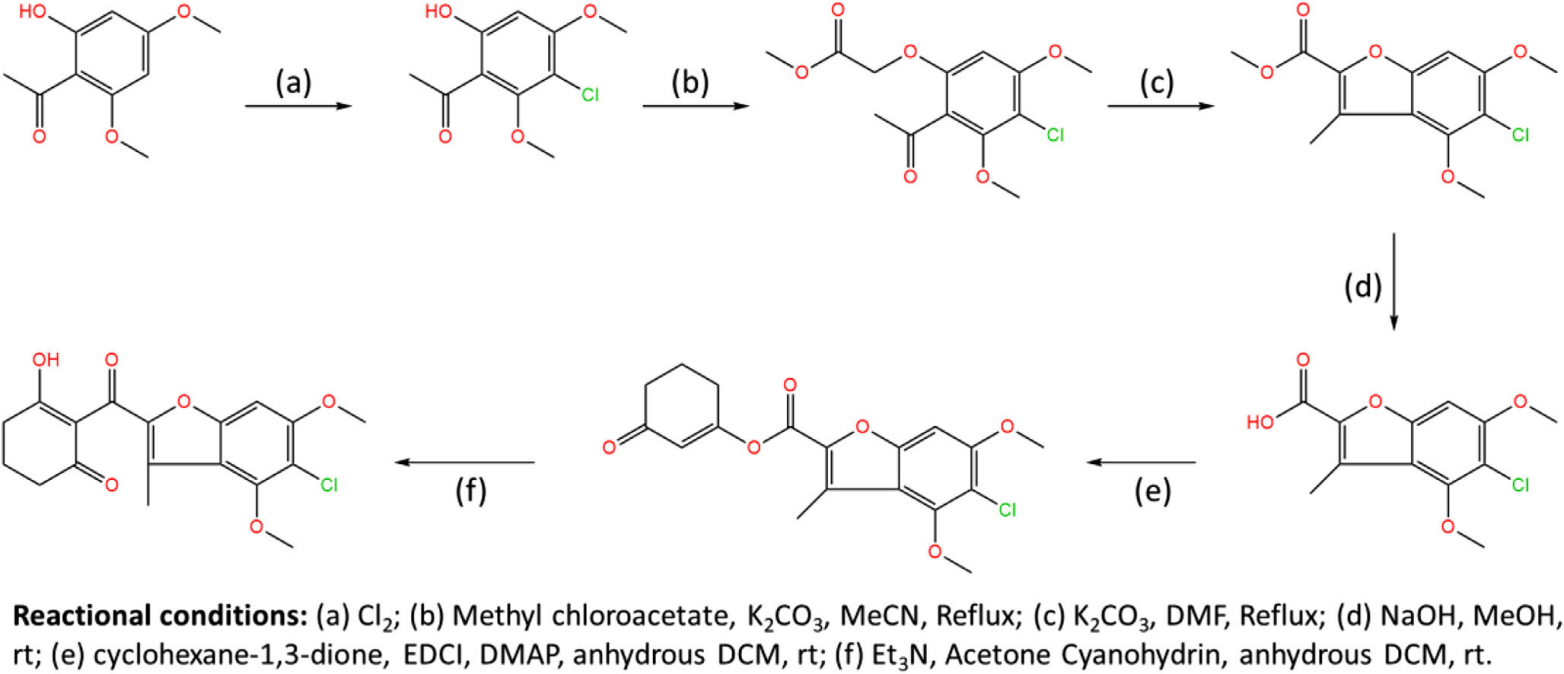
Synthetic route for **P1** compound synthesis inspired by the original article.

As shown in Table 3, compounds **P1** and **P2** had the highest predicted activity values, followed by **P8** and **P9**. Moreover, 6 of the 14 proposed molecules displayed higher predicted activity than the reference compound mesotrione (p*K*i = 7.699), with 9 of them surpassing the activity of all other compounds. Based on the original Wang et al. report [4], the synthetic route for **P1**, the molecule with the highest predicted activity, was developed to provide guidance for its future synthesis. The proposed route is illustrated in Figure 3.

### 3.2 Molecular Docking Protocol

For the docking methodology, the selected enzymatic complex was used in the redocking procedure, where the crystallographic ligand served as the reference in activity assays. To evaluate the obtained poses, the Docking Accuracy (DA) equation was applied as described in the methodology section. In the first range, defined by an RMSD of up to 2 Å from the crystallized pose, 83 poses were found, while the remaining 17 were located within the second range, between 2 and 3 Å. Given that all poses were distributed within these predefined ranges and considering the high DA value obtained (DA = 0.915), the adopted docking methodology was considered suitable.

Among all compounds, representative samples were selected based on their activity performance and interaction energy profiles. These included: the best compounds from each original dataset (**A7** and **B9**), the least active sample (**B1**), the best proposed compound (**P1**), and the crystallized ligand (Mesotrione), along with its best redocked pose (69th pose). Their binding energy components are summarized in Table 4. The free binding energy was calculated as the sum of all interaction energies, excluding the internal energy. In this context, torsional and internal terms describe ligand intramolecular contributions, while electrostatic and van der Waals terms represent intermolecular interactions with the enzymatic cavity.

**TABLE 4:** Decomposition of binding free energy values obtained from AutoDock 4 for the most favorable pose of each compound (kcal/mol).

Samples	ΔG _bind._	Electro.	VdW	Internal	Torsional
**B9**	−7.99	−2.24	−7.55	−0.41	1.79
**A7**	−9.12	−1.68	−8.33	−0.63	0.89
**B1**	−8.56	−2.32	−7.73	−0.05	1.49
**P1**	−9.57	−1.48	−9.58	−1.39	1.49
**Mesotrione Cryst**.	−6.36	−2.81	−3.97	−1.06	1.49
**Mesotrione – 69^th^**	−6.56	−2.05	−6.01	−2.15	1.49

Regarding the binding free energy calculated with AutoDock 4, the **P1** compound was identified as the most stable ligand inside the enzymatic cavity. Although **B9** exhibited the highest experimental activity within the calibration set, its binding free energy was less favorable than that of both **B1** and **A7**.

To better understand ligand accommodation within the enzyme's active site, key molecular interactions were analyzed. Metal chelation appears to be the primary factor underlying the ligand's competitive inhibitory behavior—preventing substrate binding and, consequently, catalytic activity. In addition, π–π stacking interactions between the ligand's aromatic moiety and phenylalanine residues (PHE321 and PHE356 in the 5YWG structure) support an effective binding mode consistent with inhibition [25, 61]. Considering that the docking protocol treats the protein as a rigid system, any structural variations in ligand fitting mainly reflect differences in how individual inhibitor moieties interact with the surrounding amino acid residues. Figures 5 and 6 depict the three‐dimensional orientations of the ligand conformations and highlight the specific interactions established within the binding cavity.

**FIGURE 4:**
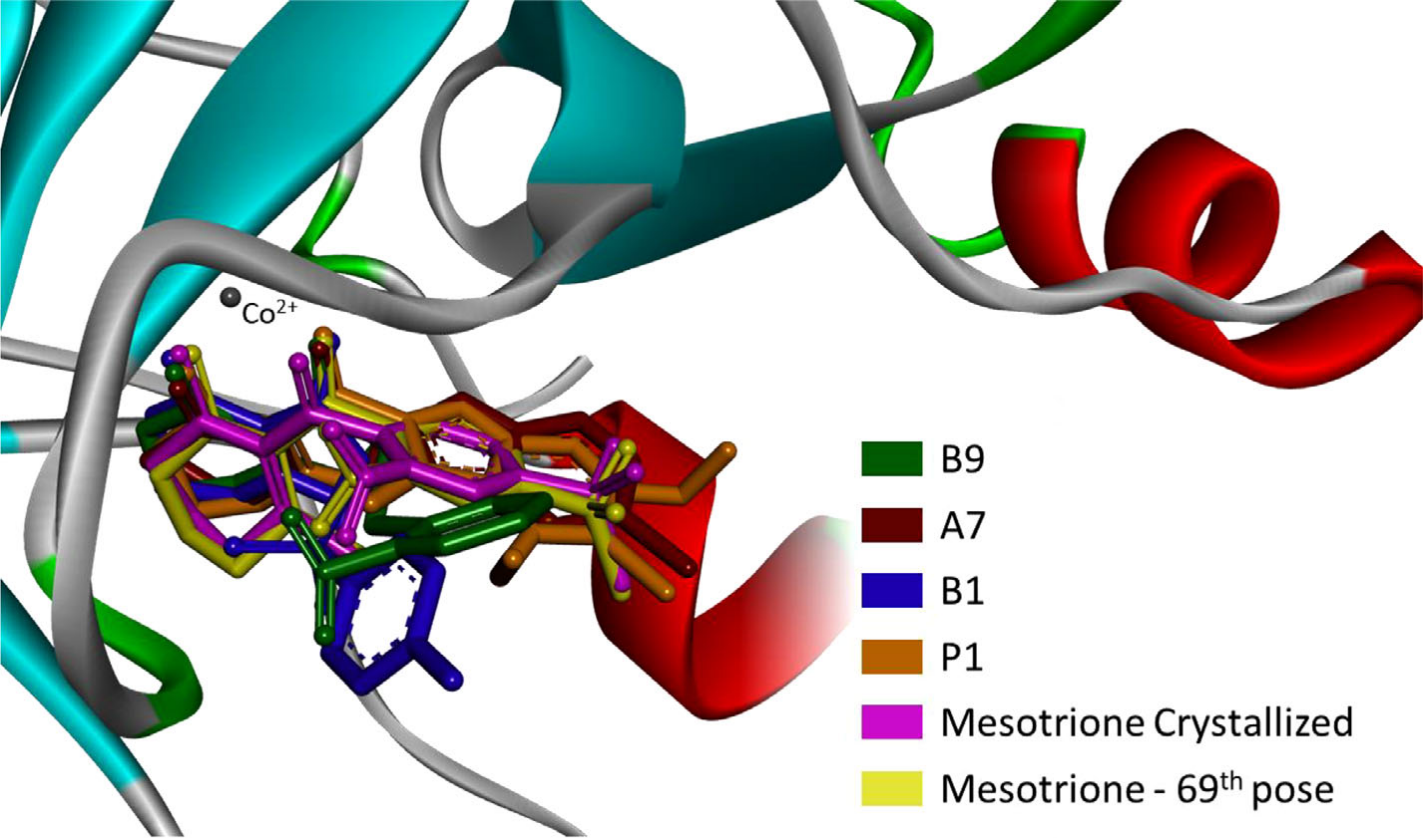
Superimposition of the most suitable ligand poses of the standard, worst, best, and proposed compounds within the enzyme cavity.

From Figure 4, while **B1** and **B9** exhibited greater deviations in their aromatic moieties, the remaining compounds showed better structural alignment. Notably, analysis of Figure 6 indicates that all presented compounds were able to chelate the cobaltous ion. Regarding π–π stacking interactions, only **B9** failed to establish an interaction with the PHE356 residue, although the expected interaction with PHE321 was preserved. The compound **A7** displayed the most similar interactions to the crystallized ligand, maintaining both aromatic and LYS353 alkyl interactions. However, despite adopting a different binding mode, **P1** exhibited the lowest binding energy among the compounds. As shown in Table 4, this is likely due to a strong Van der Waals contribution, reflected in the alkyl interactions (Pi‐alkyl: HIS248, PHE321; alkyl: LYS353, LEU308, and LEU359).

**FIGURE 5:**
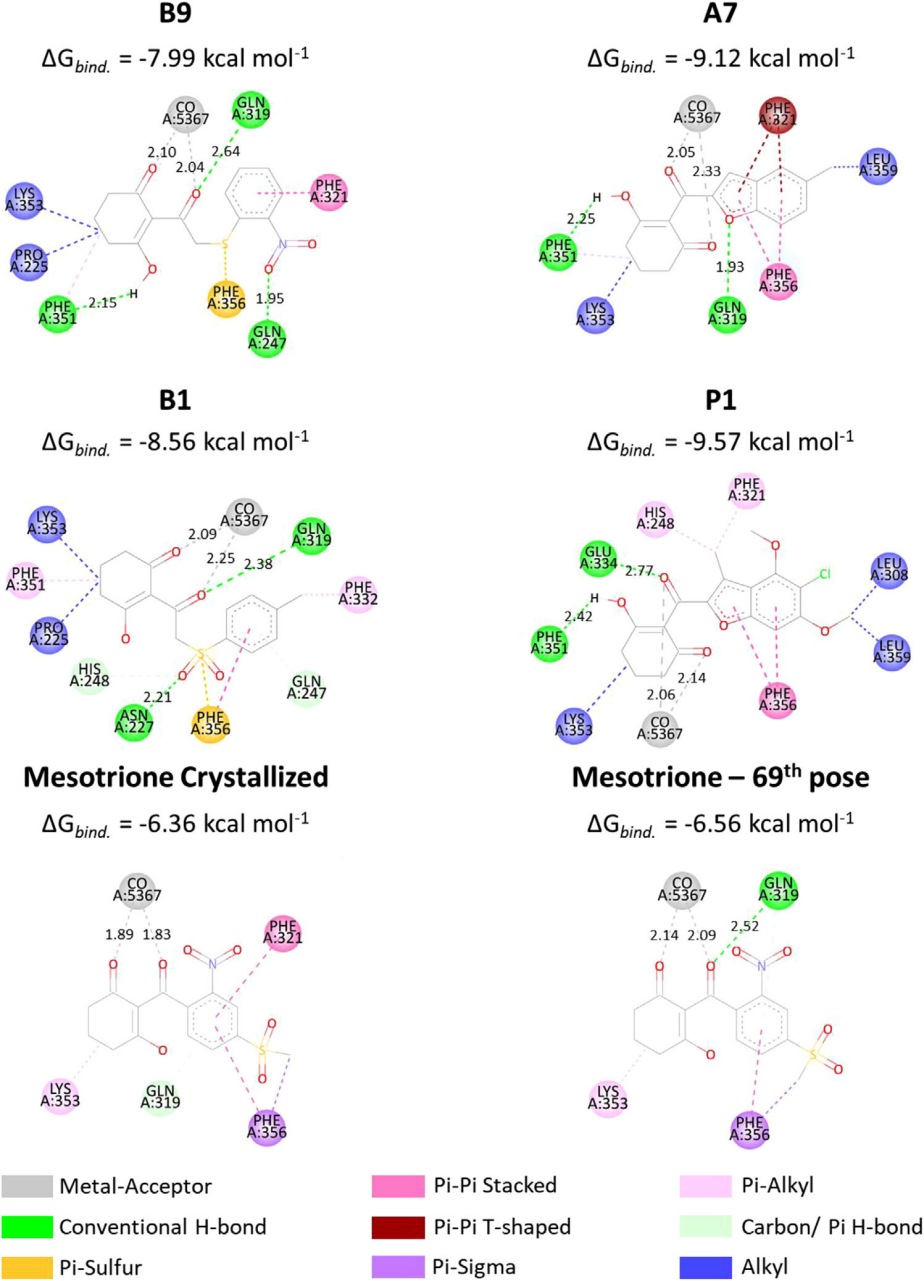
Docking‐derived interaction diagrams of the studied ligands.

**FIGURE 6:**
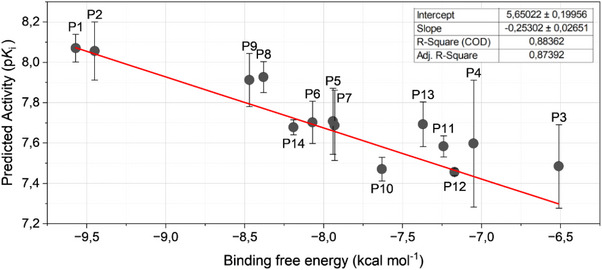
Binding energy versus predicted activity correlation of the proposed compounds.

To assess the correlation between the QSAR‐predicted activities and the binding free energies of the 14 proposed compounds, a plot was constructed (Figure 6). The X‐axis represents the binding free energy obtained from the most favorable docking poses, while the Y‐axis corresponds to the three activity values derived from each periodic property used in the MIA‐QSAR procedure, as denoted in Table 3. For each sample, the error bars on Y were calculated as the standard deviation of the three measurements, and a linear regression was performed.

The linear fit yielded a slope of –0.25 ± 0.03 and an intercept of 5.6 ± 0.2. The correlation coefficients indicate a strong agreement between predicted activity and binding affinity (R^2^ = 0.884; adjusted R^2^ = 0.874). Compounds **P1** and **P2** occupy nearly identical positions on the plot, corresponding to the highest predicted activity and the lowest binding free energy. Additional analyses were performed to ensure the reliability of the results: outlier analysis gave studentized residual as 2.7084, and the lack‐of‐fit test yielded F = 1.177 with p = 0.345, indicating no significant deviation from linearity and confirming that the linear model adequately represents the system.

### 3.3 Molecular Dynamics

Molecular dynamics simulations were performed to evaluate the binding stability of each ligand within the enzyme cavity. Three ligands were selected for comparison: the reference ligand mesotrione, **P1** (the top candidate from docking and QSAR analyses), and **P2** (the second‐best candidate). For each ligand, the initial pose corresponded to the lowest‐energy conformation obtained from docking.

**FIGURE 7:**
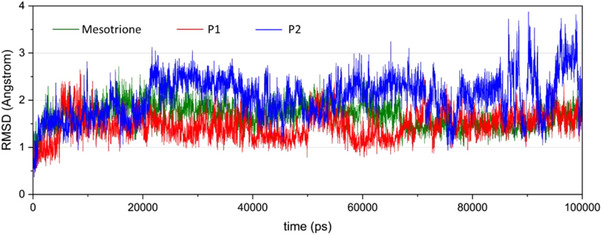
Mesotrione and the two best molecules root mean square deviation (RMSD) in the enzymatic cavity over time.

The analysis considered ligand RMSD convergence within the cavity, conservation of key residues via RMSF, and structural flexibility of both enzyme and ligand through B‐factors. As shown in Figure 7, all ligands converged within the first 20,000 ps, with fluctuations confined to the 1–3 Å range. No deviations exceeded 4 Å over the 100 000 ps simulation, although **P2** showed slightly higher deviations between 80 000 ps and the end of the simulation.

From the generated trajectories, mesotrione and **P1** exhibited the most similar RMSD behavior, with the **P1** curve showing slightly smaller deviations until convergence at 80 000 ps.

**FIGURE 8:**
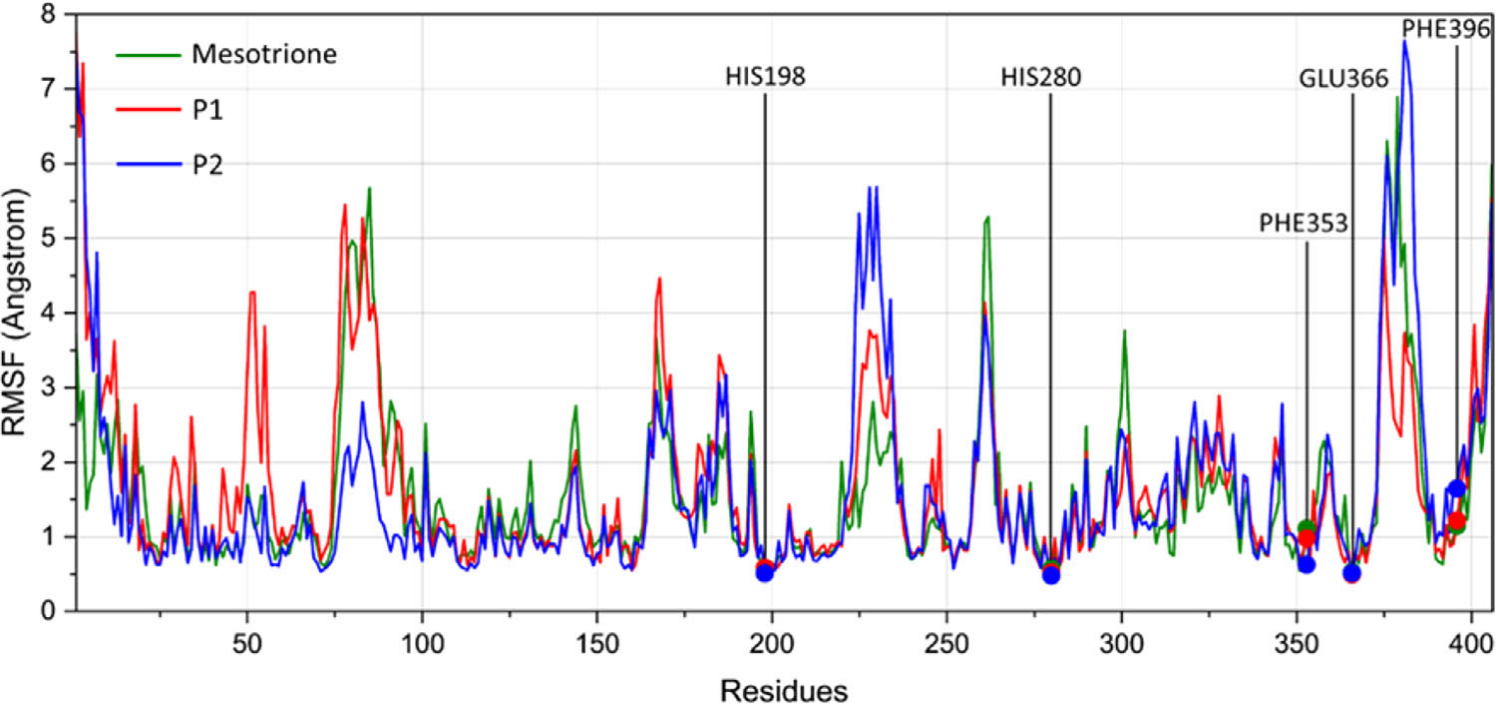
Root mean square fluctuation (RMSF) values by amino acid residues from molecular dynamics analysis of the three systems.

RMSF analysis (Figure 8) identified five key enzyme residues: His198, His280, and Glu366, which coordinate the cobalt (II) ion, and Phe353 and Phe396 (previously Phe321 and Phe356 in the docking analysis, as no loop modeling was performed), which are involved in π–π stacking interactions with the ligand's aromatic moiety. Due to the absence of coil regions in the crystallized 5YWG structure, some modeled residues displayed slight positional differences compared to the docking results. Across all monitored residues and ligand types, no deviations greater than 2 Å were observed. For the metal‐coordinating residues, the mean RMSF was 0.5 Å, whereas the phenylalanine residues exhibited greater flexibility over time. Specifically, Phe353 was more flexible in the presence of mesotrione (RMSF = 1.105 Å), while Phe396 showed increased flexibility with **P2** (RMSF = 1.641 Å).

Although the small deviations observed for these residues during the dynamic analysis suggest structural conservation of the enzyme cavity in the presence of ligands, some regions of the enzyme exhibited higher deviations. To identify these flexible regions, B‐factors were obtained, and colored structural representations were generated using DiscoveryStudio. The B‐factor is an index used to describe the inherent flexibility of atoms in a crystal lattice; considering thermal vibrations, it reflects atomic displacements around equilibrium positions throughout the trajectory and is strongly correlated with RMSF values [52].

**FIGURE 9:**
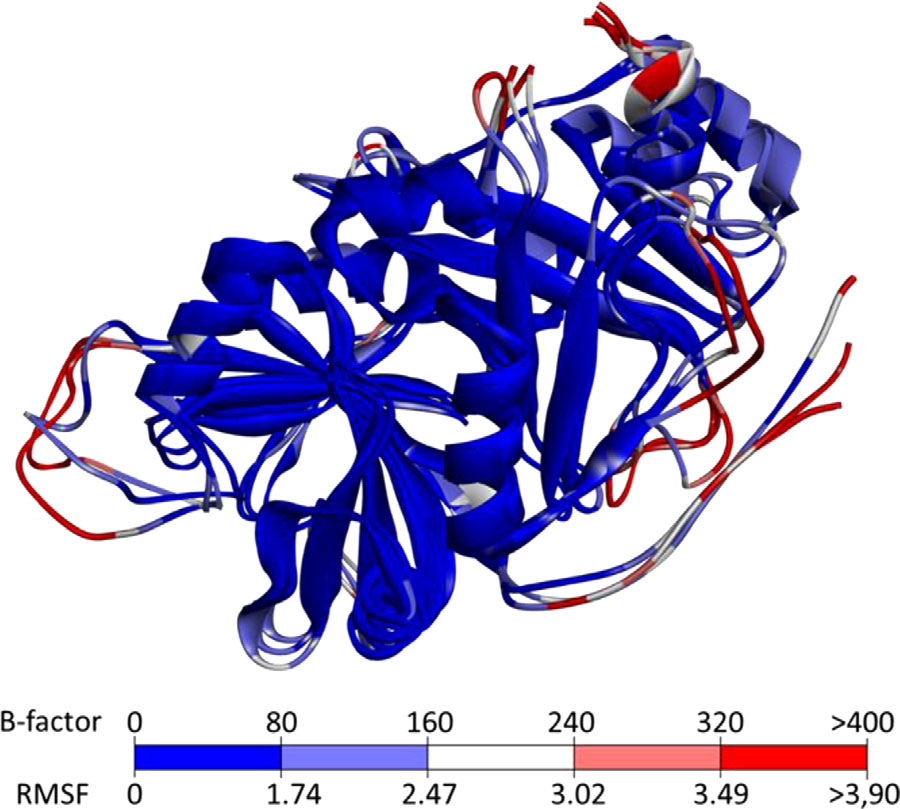
Superimposition of 4‐hydroxyphenylpyruvate dioxygenase (HPPD) from molecular dynamics analysis of the three systems, with amino acid residues colored according to B‐factors.

Given the similar findings across all molecular dynamics simulations, the macromolecular structures were superimposed. The coloring scale represents structural flexibility, with blue indicating the most conserved regions and red indicating the most flexible areas, as determined from B‐factors and atomic fluctuations around equilibrium positions (Figure 9).

In the superimposed structural representations, unfolded regions—such as loops and terminal segments—showed larger deviations and were highlighted as white/red residues, indicating a lack of convergence. In contrast, well‐defined secondary structure elements exhibited smaller deviations, reflecting their stability over time. These observations, consistent with previous HPPD studies, suggest that despite the presence of different ligands in the enzyme cavity, the overall effects of exogenous molecules on enzyme flexibility were similar [62].

### 3.4 Physicochemical Properties and ADMET Analysis

To assess the potential risks associated with the **P1** molecule compared to the standard compound mesotrione, ADMET parameter predictions were performed. Predictions were conducted using the ADMETlab web server, chosen for its high accuracy, precision, and coverage [63]. The obtained results are summarized in Table 5.

**TABLE 5:** ADMET properties calculated for **P1** and mesotrione.

Parameter	P1	Mesotrione
**Solubility**	−4.246	−3.135
**Caco‐2**	−4.710	−4.582
**BBB**	0.035	0.005
**PPB**	97.088%	58.991%
**Hepatoxicity**	0.596	0.583
**Skin sensibilization**	0.396	0.755
**Carcinogenicity**	0.581	0.579

Given the output values for each evaluated property, BBB penetration was assessed by the predicted probability of crossing the Blood‐Brain Barrier (range: 0–1), PPB by the Plasma Protein Binding index, and toxicity, skin sensitization, and carcinogenicity by the probability of being toxic or sensitizing (0 = false; 1 = true). Comparing **P1** with mesotrione, it is evident that mesotrione exhibits higher solubility. Conversely, parameters such as Caco‐2 permeability, BBB penetration, hepatotoxicity, and carcinogenicity show similar performance for both compounds. Specifically, both molecules display low predicted intestinal absorption (with ‐5.15 log units as the lower threshold) and limited BBB permeability, along with a moderate probability of hepatic and cardiac toxicity. The most pronounced differences between the two compounds were observed for PPB and skin sensitization. **P1** demonstrated a higher capacity for plasma protein binding, whereas mesotrione exhibited a greater likelihood of inducing allergenic or sensitizing effects.

**FIGURE 10:**
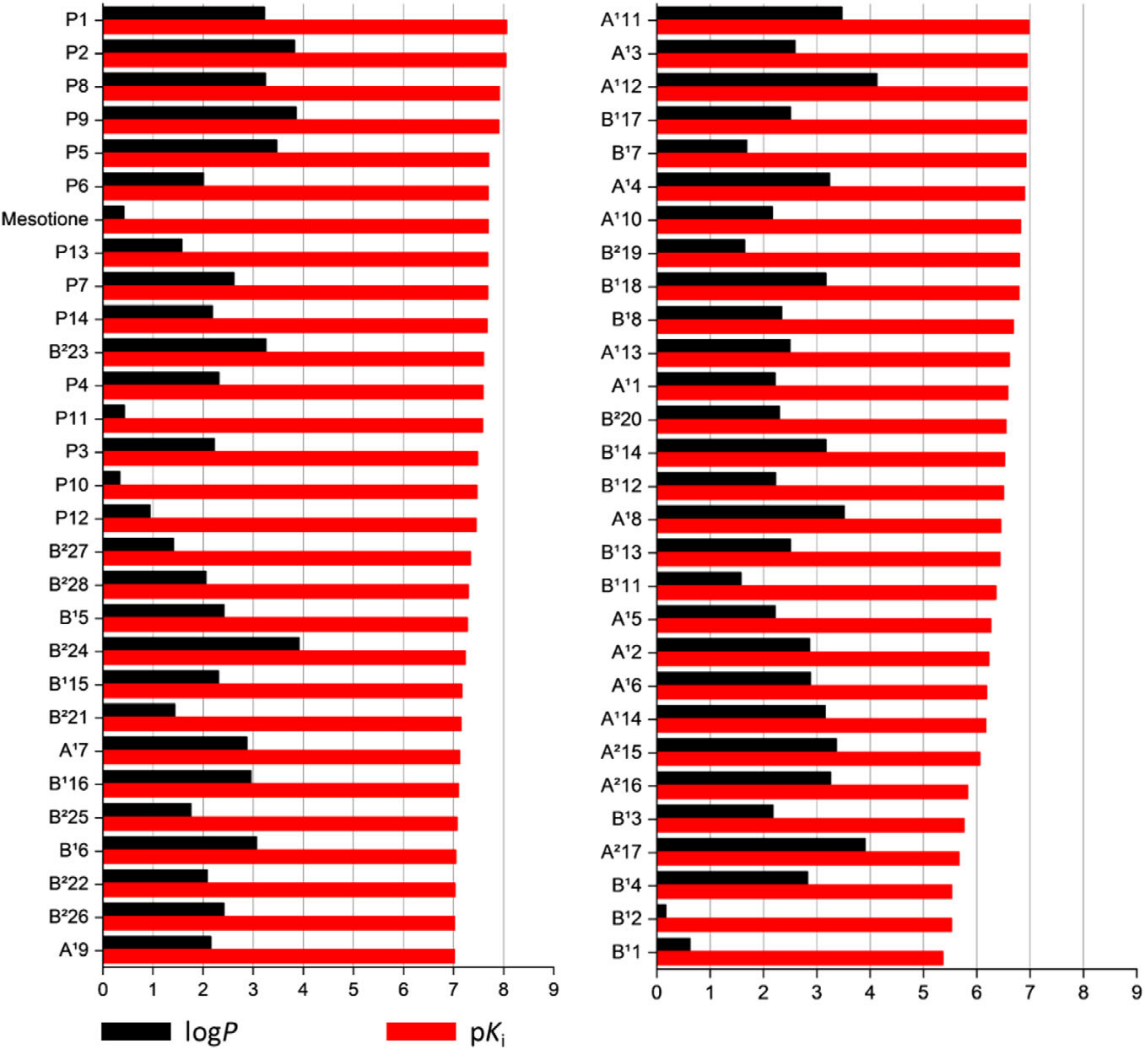
p*K*i and log *P* plots ranked by activity factor.

Regarding lipophilicity, the tenth proposed compound displayed the lowest log *P* value (0.34), compared with 0.42 for mesotrione (Figure 10). In contrast, the most active compound, **P1**, presented a log *P* value of 3.23, indicating considerably higher lipophilicity.

## 4 Conclusion

The dataset used was suitable for applying the MIA‐QSAR method, providing strong evidence that **P1** is the most promising herbicidal candidate among the studied and proposed compounds. Its potential was further supported by docking and molecular dynamics simulations.

In docking analyses, the high R^2^ value between predicted activities and binding energies indicates not only a strong correlation between these parameters but also the reliability of the original in vitro activity data, the close link between compound interactions and biological activity, and the effectiveness of the computational methods employed. During molecular dynamics simulations, **P1** and mesotrione exhibited similar behavior and convergence patterns, suggesting that **P1** could effectively inhibit HPPD, analogous to mesotrione. ADMET analysis further indicated comparable safety profiles, with **P1** showing even greater predicted safety, although appropriate management practices remain necessary.

This study provides an initial framework for designing new selective and potent herbicides. The higher predicted activity of **P1** compared to the commercial standard could translate into improved inhibition and potentially allow reduced application rates. Overall, these findings underscore the value of this computational approach for advancing environmentally sustainable and safe herbicidal design.

## Funding

This work was supported by Coordenação de Aperfeiçoamento de Pessoal de Nível Superior (CAPES, funding code 001), Conselho Nacional de Desenvolvimento Científico e Tecnológico (CNPq, grant number 306830/2021‐3), and Fundação de Amparo à Pesquisa do Estado de Minas Gerais (FAPEMIG).

## Conflicts of Interest

The authors declare no conflicts of interest.

## Supporting information




**Supporting File 1**: cbdv70695‐sup‐0001‐SuppMat.docx

## Data Availability

The authors have nothing to report.
