# Supplementary material for: In Silico Design and Validation of a Novel HPPD‐Inhibiting Herbicide Candidate Based on Benzofuran and Arylthioacetic Acid Scaffolds
Source: Chem Biodivers. 2025 Nov 17;23(1):e03221. doi: 10.1002/cbdv.202503221 (PMC12761364; doi:10.1002/cbdv.202503221)
Supplement: Supplementary file 1 — Supporting File 1: cbdv70695‐sup‐0001‐SuppMat.docx [file CBDV-23-e03221-s001.docx]

**Supporting Information**

**In Silico Design and Validation of a Novel HPPD-Inhibiting Herbicide Candidate Based on Benzofuran and Arylthioacetic Acid Scaffolds**

Luiz R. Capucho,^a^ Elaine F. F. Cunha,^a^ Matheus P. Freitas^a,^*

*^a^ Department of Chemistry, Institute of Natural Sciences, Federal University of Lavras, 37200-900, Lavras, MG, Brazil.*

** Corresponding author: matheus@ufla.br*

Summary

[S.I. 1 - Outlier analysis for electronegativity based generated models. 3](#_Toc212754862)

[S.I. 2 - Outlier analysis for Van derWaals radius based generated models. 4](#_Toc212754863)

[S.I. 3 - Outlier analysis for van der Waals radius/electronegativity ratio based generated models. 5](#_Toc212754864)

[S.I. 4. - Improvement of statistical parameters by sample deletion in output analysis 6](#_Toc212754865)

[S.I. 5 - Samples used as external group for model validation 7](#_Toc212754866)

[S.I. 6 – QSAR models statistic validation – Electronegativity 8](#_Toc212754867)

[S.I. 7 – QSAR models statistic validation – Van derWaals radius 9](#_Toc212754868)

[S.I. 8 - QSAR models statistic validation – van der Waals radius/electronegativity ratio 10](#_Toc212754869)

[S.I. 9 - MIA plot’s: PLS regression 11](#_Toc212754870)

[S.I. 10 - MIA plot’s: VIP 14](#_Toc212754871)

[S.I. 11 - Activity prediction of proposed compounds based on electronegativity 17](#_Toc212754872)

[S.I. 12 - Activity prediction of proposed compounds based on van der Waals radius 18](#_Toc212754873)

[S.I. 13 - Predicted activity of proposed compounds derived from van der Waals radius/electronegativity ratio 19](#_Toc212754874)

[S.I. 14 - Ranking of compounds according to pKi, with corresponding Log P values 20](#_Toc212754875)

[S.I. 15 - Docking output: mesotrione in the crystallographic HPPD structure (PDB 5YWG) 21](#_Toc212754876)

[S.I. 16 - Docking output: mesotrione best pose (pose 69, lowest RMSD) 22](#_Toc212754877)

[S.I. 17 - Docking output: B9 compound lowest-energy pose 23](#_Toc212754878)

[S.I. 18 - Docking output: B1 compound lowest-energy pose 24](#_Toc212754879)

[S.I. 19 - Docking output: A7 compound lowest-energy pose 25](#_Toc212754880)

[S.I. 20 - Docking output: P1 compound lowest-energy pose 26](#_Toc212754881)

[S.I. 21 - Docking output: P2 compound lowest-energy pose 27](#_Toc212754882)

[S.I. 22 - Docking output: P3 compound lowest-energy pose 28](#_Toc212754883)

[S.I. 23 - Docking output: P4 compound lowest-energy pose 29](#_Toc212754884)

[S.I. 24 - Docking output: P5 compound lowest-energy pose 30](#_Toc212754885)

[S.I. 25 - Docking output: P6 compound lowest-energy pose 31](#_Toc212754886)

[S.I. 26 - Docking output: P7 compound lowest-energy pose 32](#_Toc212754887)

[S.I. 27 - Docking output: P8 compound lowest-energy pose 33](#_Toc212754888)

[S.I. 28 - Docking output: P9 compound lowest-energy pose 34](#_Toc212754889)

[S.I. 29 - Docking output: P10 compound lowest-energy pose 35](#_Toc212754890)

[S.I. 30 - Docking output: P11 compound lowest-energy pose 36](#_Toc212754891)

[S.I. 31 - Docking output: P12 compound lowest-energy pose 37](#_Toc212754892)

[S.I. 32 - Docking output: P13 compound lowest-energy pose 38](#_Toc212754893)

[S.I. 33 - Docking output: P14 compound lowest-energy pose 39](#_Toc212754894)

S.I. 1 - Outlier analysis for electronegativity based generated models.

|  | Complete model | Without B9 sample | Without B9 and B10 sample |
| --- | --- | --- | --- |
| Willian plot (p=2.5) | 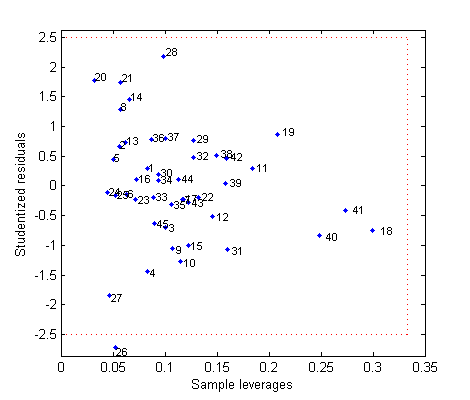 | 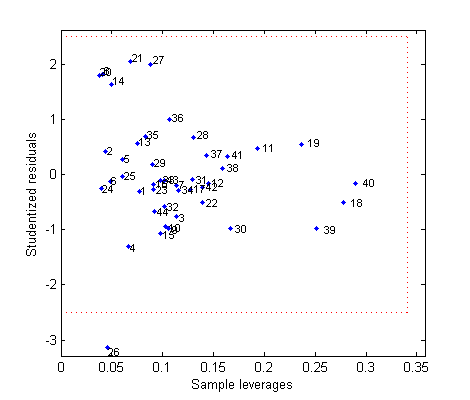 | 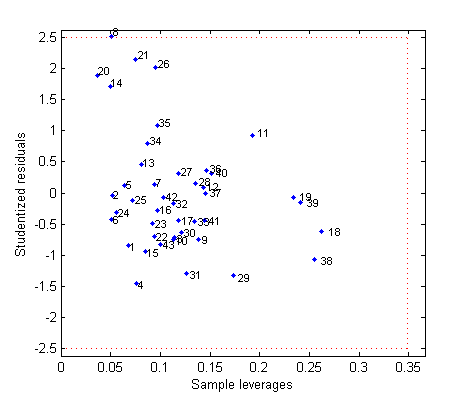 |
| Measured vs. predicted activities plot | 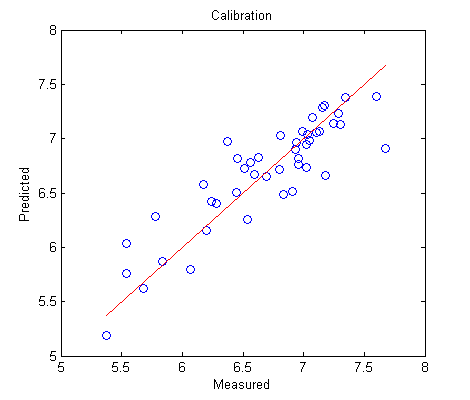 | 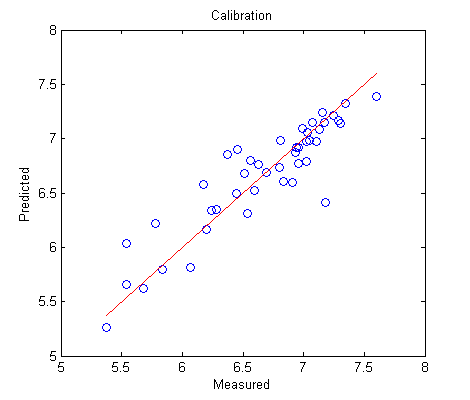 | 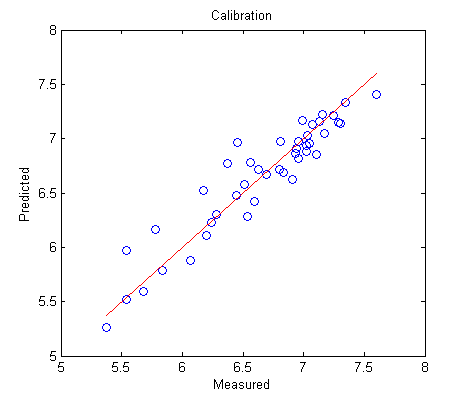 |
| Cumulative variance | \| L.V. \| X-block \| Y-Block \| \| --- \| --- \| --- \| \| 1.00 \| 13.12 \| 37.74 \| \| 2.00 \| 24.14 \| 58.42 \| \| 3.00 \| 35.01 \| 71.21 \| \| 4.00 \| 45.54 \| 75.24 \| \| 5.00 \| 51.37 \| 77.98 \| | \| L.V. \| X-block \| Y-Block \| \| --- \| --- \| --- \| \| 1.00 \| 13.55 \| 40.22 \| \| 2.00 \| 25.04 \| 62.35 \| \| 3.00 \| 35.69 \| 76.05 \| \| 4.00 \| 46.06 \| 80.16 \| \| 5.00 \| 52.67 \| 82.09 \| | \| L.V. \| X-block \| Y-Block \| \| --- \| --- \| --- \| \| 1.00 \| 13.90 \| 42.48 \| \| 2.00 \| 25.66 \| 66.80 \| \| 3.00 \| 36.15 \| 81.98 \| \| 4.00 \| 46.48 \| 86.34 \| \| 5.00 \| 52.30 \| 88.27 \| |

S.I. 2 - Outlier analysis for Van derWaals radius based generated models.

|  | Complete model | Without B9 sample | Without B9 and B10 sample |
| --- | --- | --- | --- |
| Willian plot (p=2.5) | 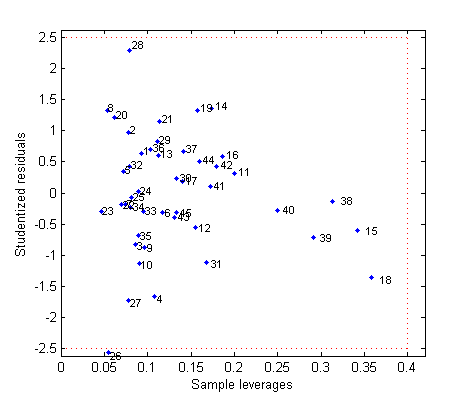 | 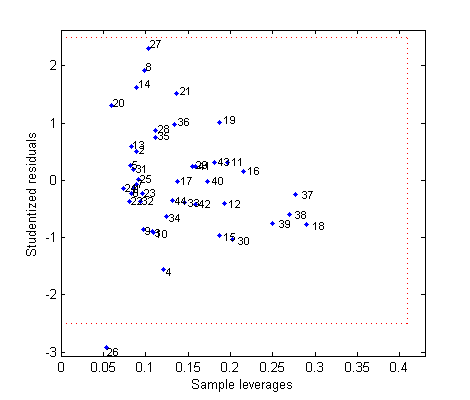 | 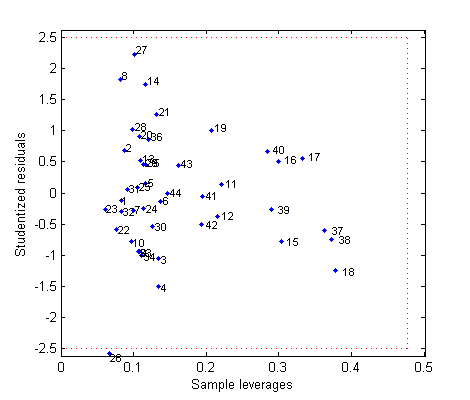 |
| Measured vs. predicted activities plot | 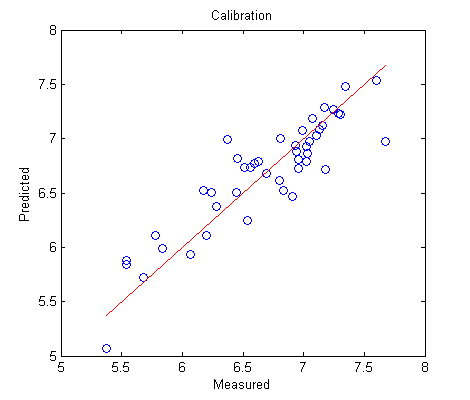 | 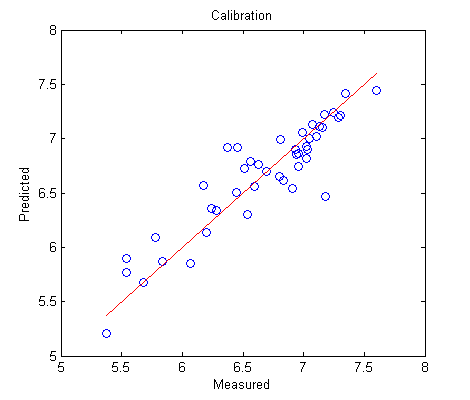 | 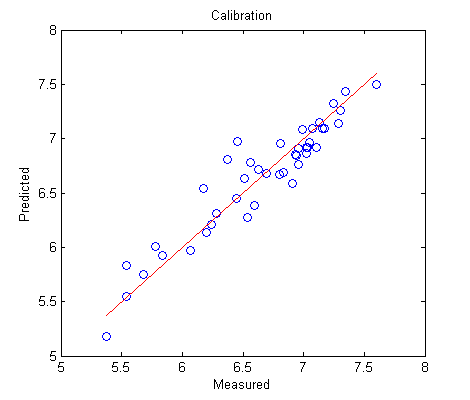 |
| Cumulative variance | \| L.V. \| X-block \| Y-Block \| \| --- \| --- \| --- \| \| 1.00 \| 16.00 \| 33.97 \| \| 2.00 \| 25.76 \| 54.10 \| \| 3.00 \| 30.28 \| 73.20 \| \| 4.00 \| 44.66 \| 75.17 \| \| 5.00 \| 49.40 \| 78.55 \| \| 6.00 \| 58.64 \| 80.41 \| | \| L.V. \| X-block \| Y-Block \| \| --- \| --- \| --- \| \| 1.00 \| 15.95 \| 35.88 \| \| 2.00 \| 25.92 \| 57.84 \| \| 3.00 \| 30.95 \| 77.23 \| \| 4.00 \| 45.71 \| 79.37 \| \| 5.00 \| 53.11 \| 81.76 \| \| 6.00 \| 65.01 \| 83.34 \| | \| L.V. \| X-block \| Y-Block \| \| --- \| --- \| --- \| \| 1.00 \| 15.95 \| 37.61 \| \| 2.00 \| 26.11 \| 61.47 \| \| 3.00 \| 31.08 \| 82.73 \| \| 4.00 \| 45.44 \| 84.88 \| \| 5.00 \| 51.96 \| 87.28 \| \| 6.00 \| 58.73 \| 89.32 \| |

S.I. 3 - Outlier analysis for van der Waals radius/electronegativity ratio based generated models.

|  | Complete model | Without B9 sample | Without B9 and B10 sample |
| --- | --- | --- | --- |
| Willian plot (p=2.5) | 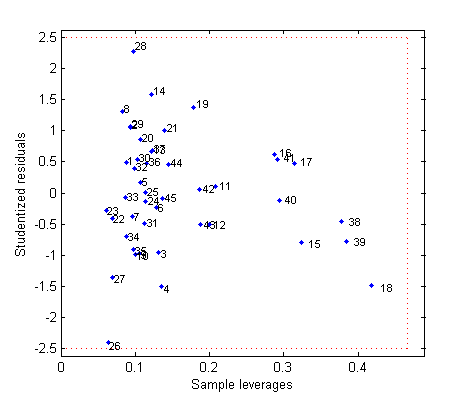 | 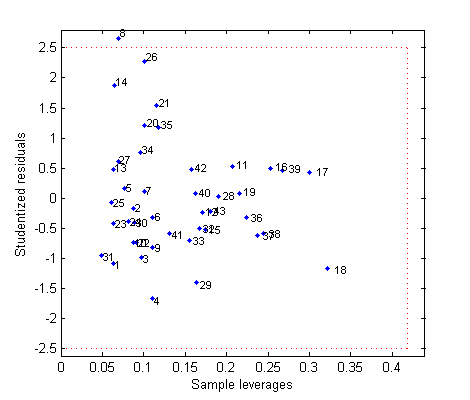 | 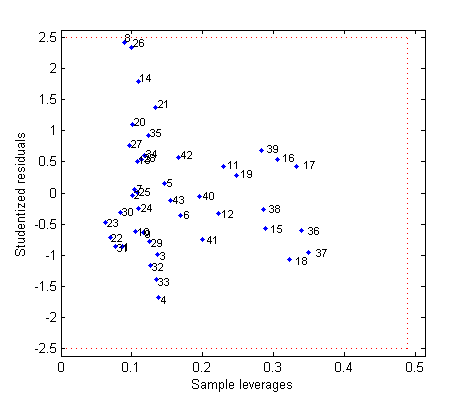 |
| Measured vs. predicted activities plot | 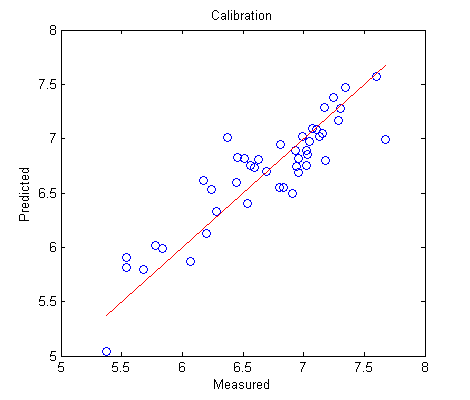 | 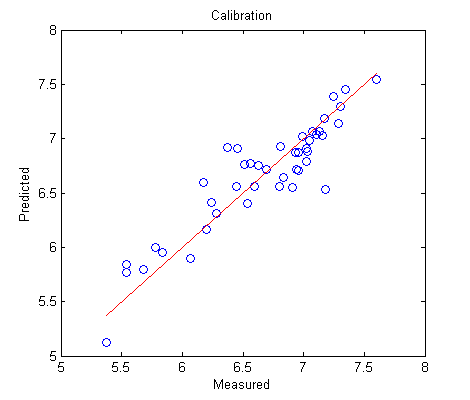 | 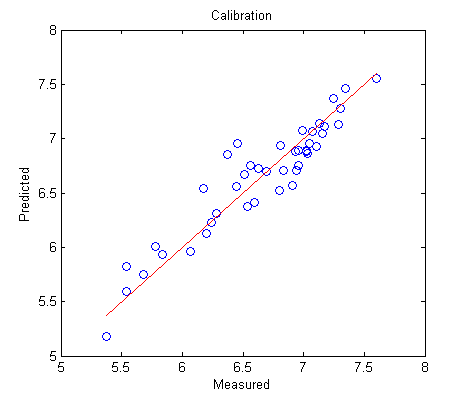 |
| Cumulative variance | \| L.V. \| X-block \| Y-Block \| \| --- \| --- \| --- \| \| 1.00 \| 18.38 \| 32.80 \| \| 2.00 \| 29.60 \| 51.99 \| \| 3.00 \| 37.71 \| 65.62 \| \| 4.00 \| 45.50 \| 73.96 \| \| 5.00 \| 59.76 \| 76.26 \| \| 6.00 \| 74.78 \| 77.58 \| \| 7.00 \| 79.29 \| 79.98 \| | \| L.V. \| X-block \| Y-Block \| \| --- \| --- \| --- \| \| 1.00 \| 15.95 \| 37.61 \| \| 2.00 \| 26.11 \| 61.47 \| \| 3.00 \| 31.08 \| 82.73 \| \| 4.00 \| 45.44 \| 84.88 \| \| 5.00 \| 51.96 \| 87.28 \| \| 6.00 \| 58.73 \| 89.32 \| \| 7.00 \| 65.89 \| 91.16 \| | \| L.V. \| X-block \| Y-Block \| \| --- \| --- \| --- \| \| 1.00 \| 17.98 \| 35.33 \| \| 2.00 \| 29.68 \| 57.49 \| \| 3.00 \| 38.34 \| 72.80 \| \| 4.00 \| 45.88 \| 82.23 \| \| 5.00 \| 57.30 \| 84.79 \| \| 6.00 \| 74.90 \| 85.86 \| \| 7.00 \| 80.26 \| 88.29 \| |

S.I. 4. - Improvement of statistical parameters by sample deletion in output analysis

|  | Electronegativity | | | Van derWaals radius | | | r_VdW_/ε | | |
| --- | --- | --- | --- | --- | --- | --- | --- | --- | --- |
|  | Complete | Without B9 | Without B9  and B10 | Complete | Without B9 | Without B9  and B10 | Complete | Without B9 | Without B9  and B10 |
| V.L. | 5.00 | 5.00 | 5.00 | 6.00 | 6.00 | 6.00 | 7.00 | 7.00 | 7.00 |
| RMSEc | 0.2611 | 0.2295 | 0.1859 | 0.2463 | 0.2213 | 0.1774 | 0.2490 | 0.2203 | 0.1857 |
| *r*² | 0.7798 | 0.8208 | 0.8827 | 0.8041 | 0.8334 | 0.8932 | 0.7998 | 0.8350 | 0.8829 |
| RMSE_y-rand_ | 0.4459 | 0.4300 | 0.4243 | 0.4491 | 0.4060 | 0.4063 | 0.4386 | 0.4137 | 0.3935 |
| *r*² _y-rand_ | 0.3563 | 0.3693 | 0.3839 | 0.3461 | 0.4342 | 0.4335 | 0.3735 | 0.4146 | 0.4679 |
| ^c^­*r*²_p_ | 0.5747 | 0.6088 | 0.6635 | 0.6069 | 0.5769 | 0.6408 | 0.5839 | 0.5925 | 0.6053 |
| RMSEcv | 0.3830 | 0.3390 | 0.2819 | 0.3747 | 0.3368 | 0.2800 | 0.3826 | 0.3426 | 0.2890 |
| *q*² | 0.5515 | 0.6183 | 0.7313 | 0.5649 | 0.6262 | 0.7388 | 0.5481 | 0.6120 | 0.7193 |

S.I. 5 - Samples used as external group for model validation

Model 1: A2;A9;A12;A16;A17;B3;B5;B7;B19;B20;B28

Model 2: A1;A6;A7;A11;A15;A16;B4;B12;B13;B15;B17

Model 3: A2;A3;A5;A6;A11;B5;B19;B20;B21;B24;B26

Model 4: A7;A9;A11;A12;B2;B5;B6;B14;B17;B21;B25

Model 5: A2;A4;A7;A11;A14;B2;B4;B8;B14;B22;B24

Model 6: A1;A3;A11;A13;B4;B7;B8;B14;B16;B18;B22

Model 7: A1;A9;A11;A12;A14;A16;B2;B8;B12;B18;B27

Model 8: A8;A10;A13;A15;A16;B5;B6;B8;B15;B19;B26

Model 9: A4;A9;A12;A17;B7;B11;B12;B18;B21;B24;B25

Model 10: A4;A10;A12;A13;A16;B2;B7;B12;B25;B26;B28

S.I. 6 – QSAR models statistic validation – Electronegativity

|  | Model 1 | Model 2 | Model 3 | Model 4 | Model 5 | Model 6 | Model 7 | Model 8 | Model 9 | Model 10 | Mean | St. Dev. |
| --- | --- | --- | --- | --- | --- | --- | --- | --- | --- | --- | --- | --- |
| V.L. | 5.00 | 5.00 | 5.00 | 6.00 | 5.00 | 7.00 | 6.00 | 5.00 | 5.00 | 4.00 | 5.30 | 0.82 |
| RMSEc | 0.1916 | 0.1884 | 0.1947 | 0.1767 | 0.1714 | 0.1577 | 0.1642 | 0.1801 | 0.1762 | 0.2013 | 0.1802 | 0.0138 |
| *r*² | 0.8685 | 0.8785 | 0.8867 | 0.8958 | 0.8934 | 0.9260 | 0.9105 | 0.9020 | 0.9043 | 0.8667 | 0.8932 | 0.0187 |
| RMSE_y-rand_ | 0.3713 | 0.3779 | 0.4274 | 0.3663 | 0.3699 | 0.4124 | 0.3958 | 0.3802 | 0.3826 | 0.4025 | 0.3886 | 0.0202 |
| *r*² _y-rand_ | 0.5015 | 0.5009 | 0.4493 | 0.5465 | 0.4990 | 0.4884 | 0.4762 | 0.5563 | 0.5367 | 0.4626 | 0.5017 | 0.0355 |
| ^c^­*r*²_p_ | 0.5646 | 0.5759 | 0.6227 | 0.5594 | 0.5936 | 0.6365 | 0.6289 | 0.5584 | 0.5766 | 0.5918 | 0.5908 | 0.0293 |
| RMSEcv | 0.3734 | 0.3642 | 0.3310 | 0.3867 | 0.3569 | 0.3548 | 0.3387 | 0.3971 | 0.3562 | 0.3748 | 0.3634 | 0.0204 |
| *q*² | 0.5113 | 0.5540 | 0.6820 | 0.5240 | 0.6205 | 0.6467 | 0.6307 | 0.5389 | 0.6169 | 0.5425 | 0.5868 | 0.0592 |
| RMSEp | 0.2895 | 0.2596 | 0.2627 | 0.2242 | 0.2383 | 0.2352 | 0.1824 | 0.2281 | 0.2529 | 0.2257 | 0.2399 | 0.0288 |
| *r*²_pred_ | 0.7738 | 0.7505 | 0.6542 | 0.7975 | 0.8442 | 0.6886 | 0.8893 | 0.7395 | 0.7034 | 0.8351 | 0.7676 | 0.0749 |
| *r*²_m_ Avg. | 0.6386 | 0.6558 | 0.4546 | 0.7163 | 0.7266 | 0.5745 | 0.7944 | 0.6466 | 0.5976 | 0.7684 | 0.6573 | 0.1008 |
| Δ*r*²_m_ | 0.1822 | 0.1858 | 0.2734 | 0.0673 | 0.1358 | 0.1326 | 0.1011 | 0.0125 | 0.0311 | 0.0740 | 0.1196 | 0.0795 |
| CCC | 0.8624 | 0.8533 | 0.7525 | 0.8896 | 0.9079 | 0.8165 | 0.9348 | 0.8535 | 0.8354 | 0.8940 | 0.8600 | 0.0517 |

S.I. 7 – QSAR models statistic validation – Van derWaals radius

|  | Model 1 | Model 2 | Model 3 | Model 4 | Model 5 | Model 6 | Model 7 | Model 8 | Model 9 | Model 10 | Mean | St. Dev. |
| --- | --- | --- | --- | --- | --- | --- | --- | --- | --- | --- | --- | --- |
| V.L. | 8.00 | 7.00 | 4.00 | 7.00 | 7.00 | 7.00 | 7.00 | 7.00 | 6.00 | 6.00 | 6.60 | 1.07 |
| RMSEc | 0.1760 | 0.1780 | 0.2215 | 0.1677 | 0.1592 | 0.1597 | 0.1594 | 0.1585 | 0.1647 | 0.1801 | 0.1725 | 0.0191 |
| *r*² | 0.8890 | 0.8915 | 0.8534 | 0.9062 | 0.9081 | 0.9241 | 0.9156 | 0.9241 | 0.9165 | 0.8934 | 0.9022 | 0.0214 |
| RMSE_y-rand_ | 0.3611 | 0.3642 | 0.4372 | 0.3631 | 0.3524 | 0.3753 | 0.3698 | 0.3939 | 0.3532 | 0.4018 | 0.3772 | 0.0266 |
| *r*² _y-rand_ | 0.5234 | 0.5350 | 0.4230 | 0.5507 | 0.5458 | 0.5692 | 0.5388 | 0.5229 | 0.5942 | 0.4652 | 0.5268 | 0.0495 |
| ^c^­*r*²_p_ | 0.5701 | 0.5637 | 0.6061 | 0.5676 | 0.5736 | 0.5726 | 0.5874 | 0.5846 | 0.5435 | 0.6184 | 0.5788 | 0.0215 |
| RMSEcv | 0.3661 | 0.3660 | 0.3785 | 0.3879 | 0.3538 | 0.3739 | 0.3446 | 0.3669 | 0.3718 | 0.3537 | 0.3663 | 0.0129 |
| *q*² | 0.5390 | 0.5600 | 0.5817 | 0.5544 | 0.5474 | 0.6075 | 0.6319 | 0.6089 | 0.5915 | 0.5967 | 0.5819 | 0.0307 |
| RMSEp | 0.2746 | 0.2234 | 0.3316 | 0.2447 | 0.2427 | 0.2322 | 0.2114 | 0.2430 | 0.3320 | 0.2190 | 0.2555 | 0.0438 |
| *r*²_pred_ | 0.8010 | 0.8219 | 0.4327 | 0.7241 | 0.8402 | 0.7053 | 0.8479 | 0.6978 | 0.5643 | 0.8801 | 0.7315 | 0.1413 |
| *r*²_m_ Avg. | 0.7219 | 0.7500 | 0.2926 | 0.6170 | 0.6804 | 0.5955 | 0.6782 | 0.5944 | 0.4318 | 0.6441 | 0.6006 | 0.1394 |
| Δ*r*²_m_ | 0.1509 | 0.1421 | 0.2063 | 0.1623 | 0.1518 | 0.1266 | 0.1538 | 0.0219 | 0.1084 | 0.1599 | 0.1384 | 0.0481 |
| CCC | 0.8848 | 0.8936 | 0.6220 | 0.8453 | 0.8995 | 0.8211 | 0.9026 | 0.8339 | 0.7420 | 0.8816 | 0.8326 | 0.0889 |

S.I. 8 - QSAR models statistic validation – van der Waals radius/electronegativity ratio

|  | Model 1 | Model 2 | Model 3 | Model 4 | Model 5 | Model 6 | Model 7 | Model 8 | Model 9 | Model 10 | Mean | St. Dev. |
| --- | --- | --- | --- | --- | --- | --- | --- | --- | --- | --- | --- | --- |
| V.L. | 8.00 | 7.00 | 8.00 | 7.00 | 10.00 | 4.00 | 8.00 | 9.00 | 7.00 | 10.00 | 7.80 | 1.66 |
| RMSEc | 0.1797 | 0.1834 | 0.1846 | 0.1882 | 0.1377 | 0.2243 | 0.1546 | 0.1283 | 0.1624 | 0.1382 | 0.1681 | 0.0280 |
| *r*² | 0.8844 | 0.8848 | 0.8982 | 0.8817 | 0.9312 | 0.8502 | 0.9207 | 0.9503 | 0.9188 | 0.9372 | 0.9057 | 0.0294 |
| RMSE_y-rand_ | 0.3754 | 0.3464 | 0.3931 | 0.3584 | 0.3318 | 0.4052 | 0.3935 | 0.3711 | 0.3790 | 0.3411 | 0.3695 | 0.0233 |
| *r*² _y-rand_ | 0.4861 | 0.5787 | 0.5362 | 0.5702 | 0.5960 | 0.5065 | 0.4793 | 0.5729 | 0.5530 | 0.6083 | 0.5487 | 0.0429 |
| ^c^­*r*²_p_ | 0.5934 | 0.5204 | 0.5702 | 0.5241 | 0.5587 | 0.5405 | 0.6375 | 0.5988 | 0.5797 | 0.5552 | 0.5679 | 0.0343 |
| RMSEcv | 0.3751 | 0.3791 | 0.3423 | 0.3720 | 0.3253 | 0.4071 | 0.3415 | 0.3647 | 0.3827 | 0.3043 | 0.3594 | 0.0291 |
| *q*² | 0.5173 | 0.5253 | 0.6594 | 0.5647 | 0.6202 | 0.5213 | 0.6281 | 0.6273 | 0.5757 | 0.6995 | 0.5939 | 0.0595 |
| RMSEp | 0.2795 | 0.2325 | 0.2817 | 0.2760 | 0.2488 | 0.2911 | 0.2660 | 0.2517 | 0.3404 | 0.2634 | 0.2731 | 0.0280 |
| *r*²_pred_ | 0.7909 | 0.8033 | 0.6867 | 0.6601 | 0.8743 | 0.5959 | 0.7495 | 0.6866 | 0.4992 | 0.7518 | 0.7098 | 0.1032 |
| *r*²_m_ Avg. | 0.7064 | 0.7268 | 0.5883 | 0.5121 | 0.6041 | 0.4676 | 0.5540 | 0.5802 | 0.3626 | 0.5949 | 0.5697 | 0.1013 |
| Δ*r*²_m_ | 0.1581 | 0.1314 | 0.0171 | 0.2685 | 0.1767 | 0.0166 | 0.2318 | 0.0480 | 0.0150 | 0.2160 | 0.1279 | 0.0924 |
| CCC | 0.8792 | 0.8859 | 0.7917 | 0.7744 | 0.8887 | 0.7341 | 0.8366 | 0.8270 | 0.7026 | 0.8373 | 0.8157 | 0.0609 |

S.I. 9 - MIA plot’s: PLS regression

|  | Electronegativity | Van derWalls radius | r_VdW_/ε ratio |
| --- | --- | --- | --- |
| Model 1 | 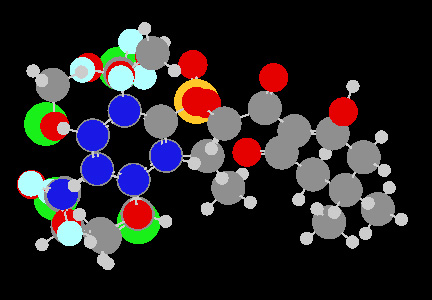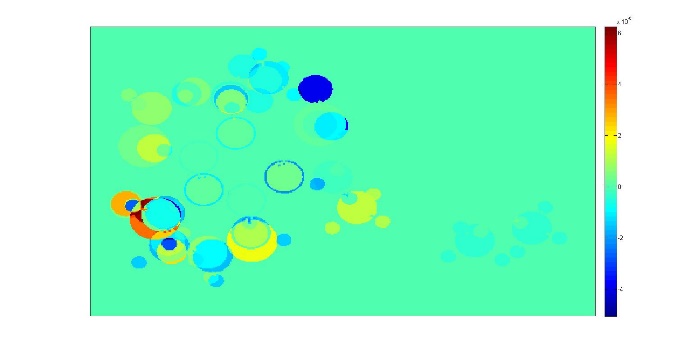 | 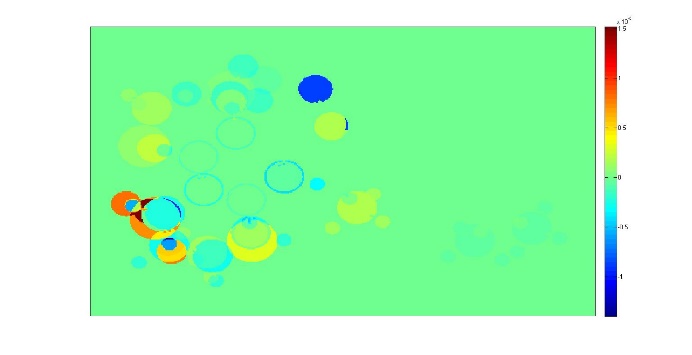 | 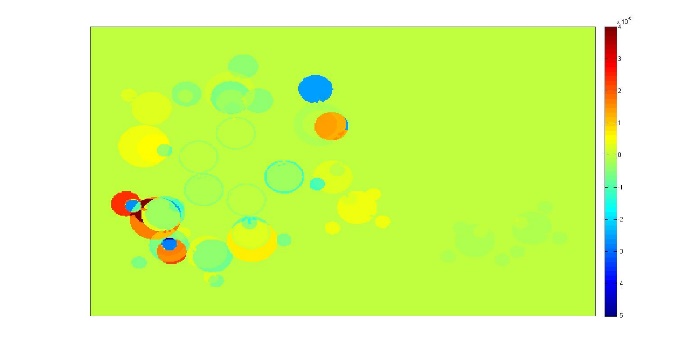 |
| Model 2 | 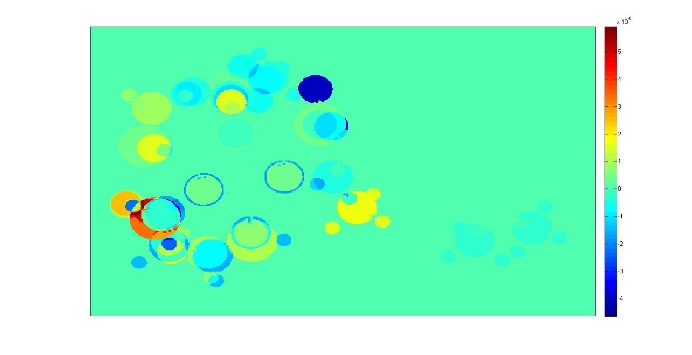 | 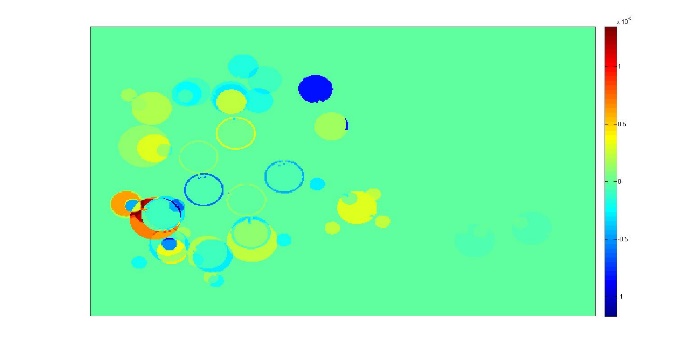 | 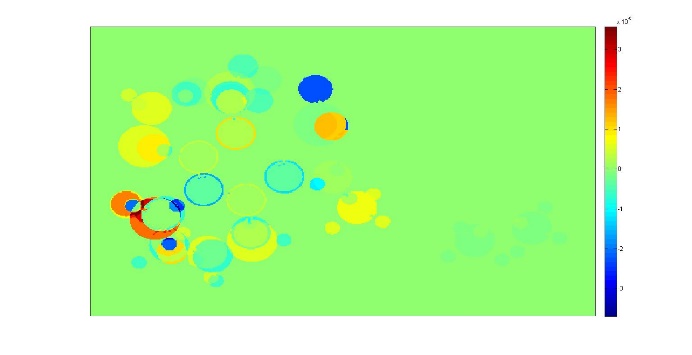 |
| Model 3 | 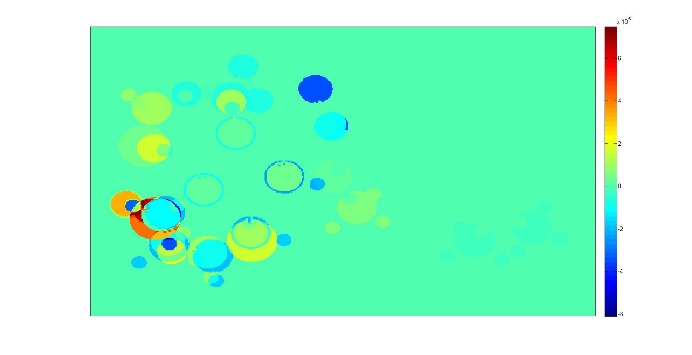 | 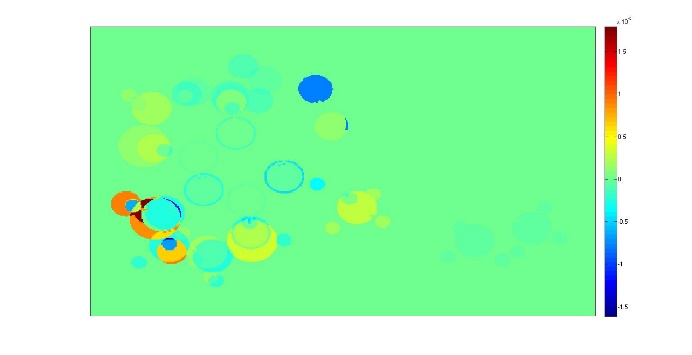 | 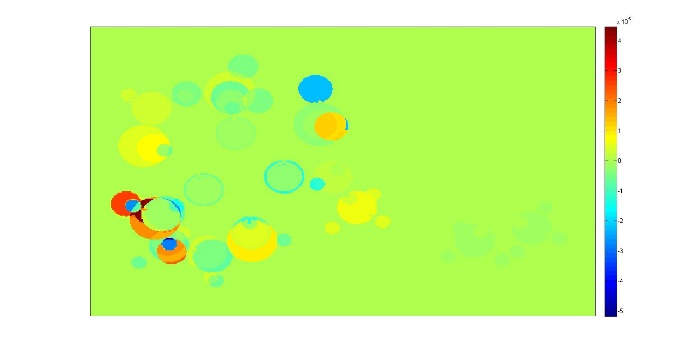 |
| Model 4 | 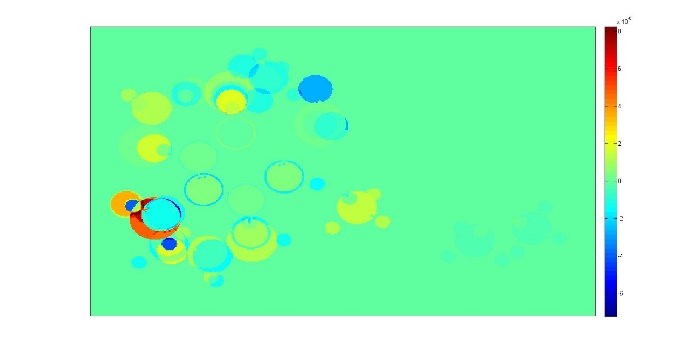 | 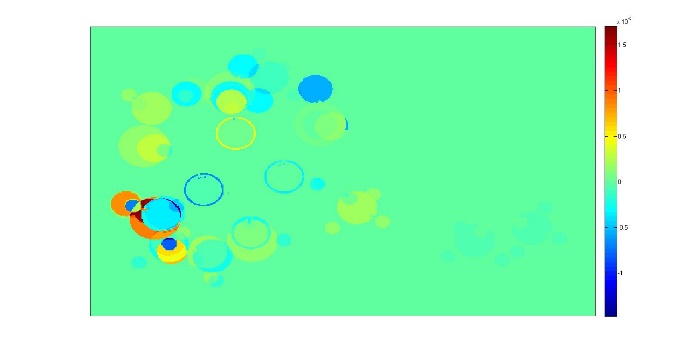 | 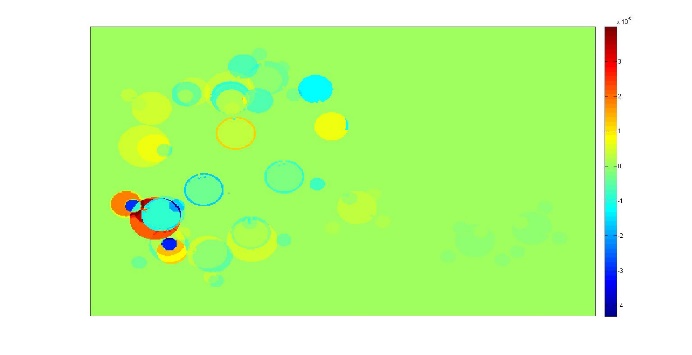 |
| Model 5 | 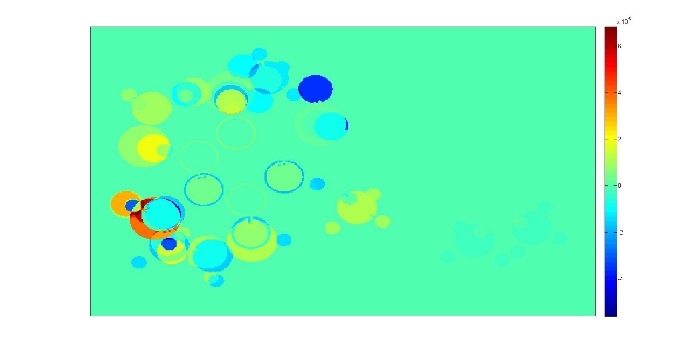 | 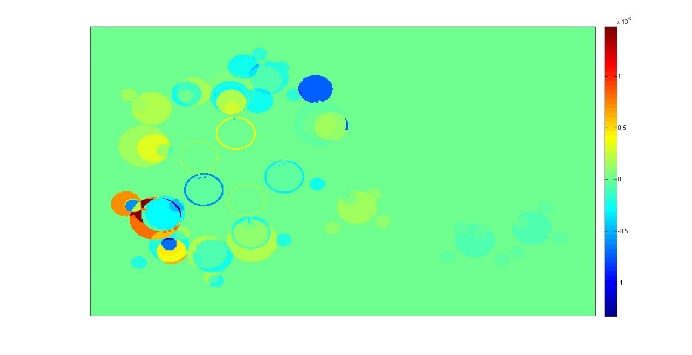 | 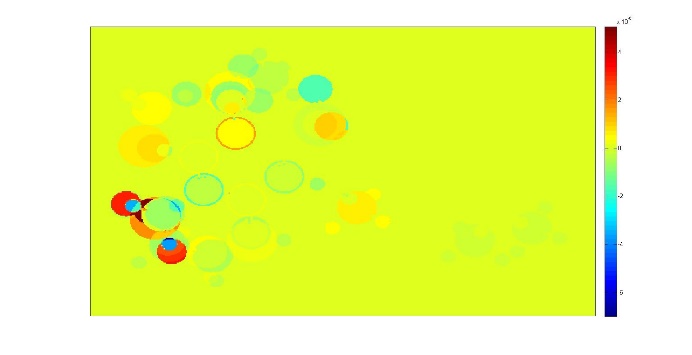 |
| Model 6 | 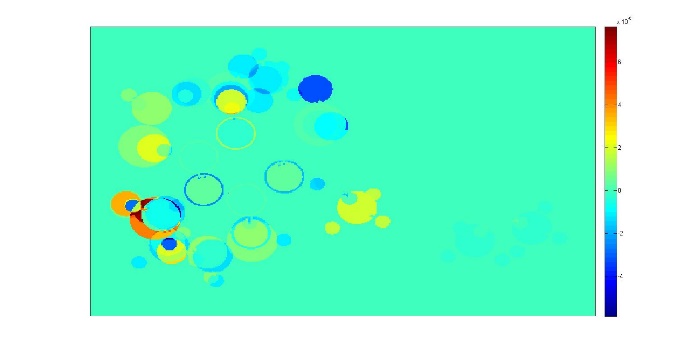 | 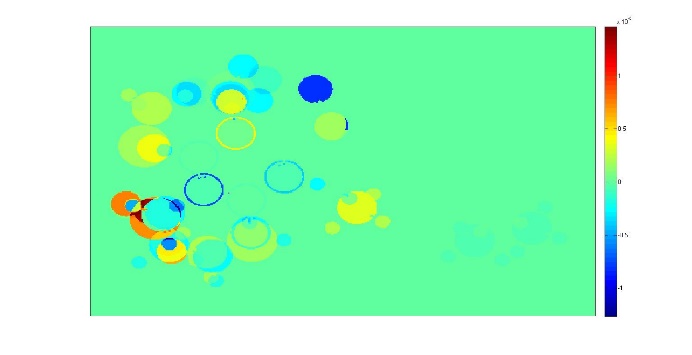 | 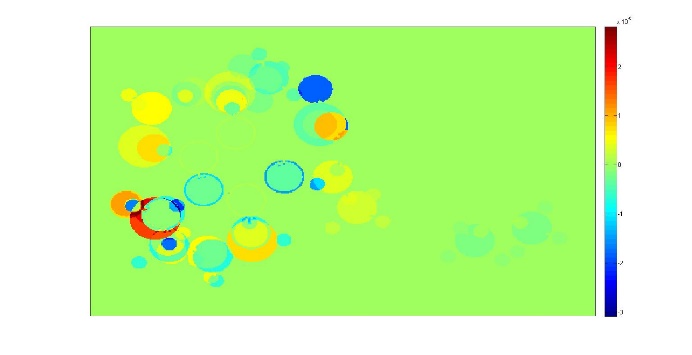 |
| Model 7 | 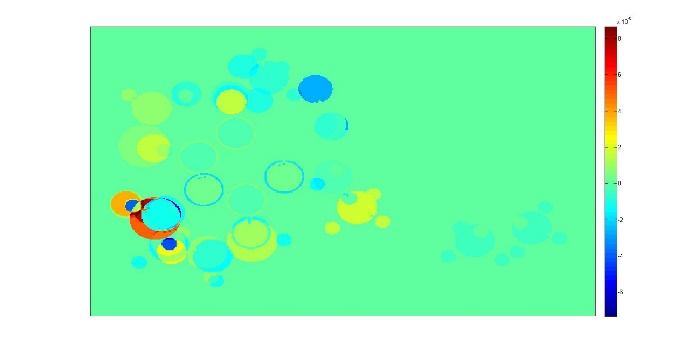 | 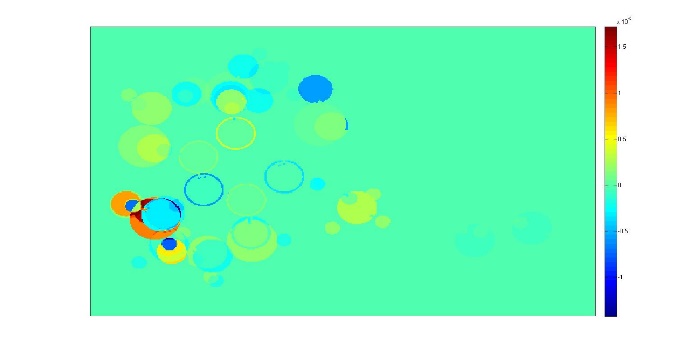 | 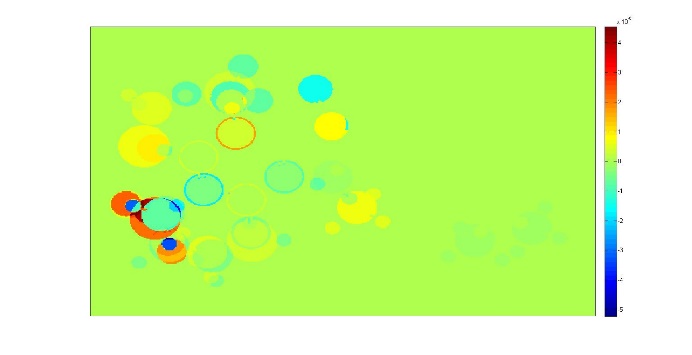 |
| Model 8 | 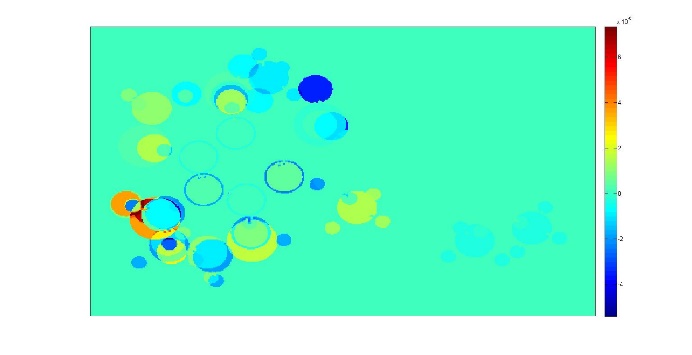 | 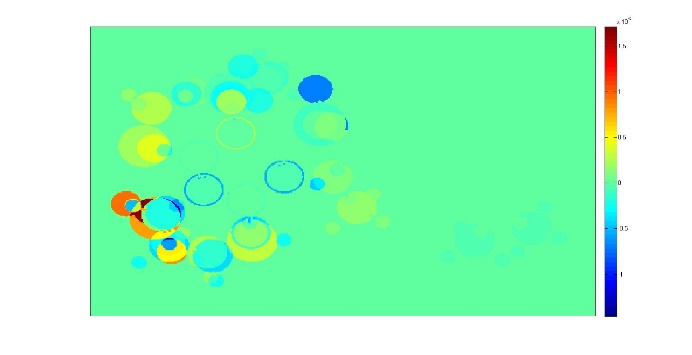 | 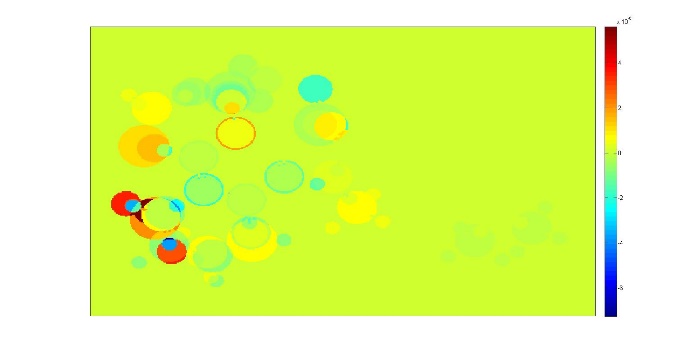 |
| Model 9 | 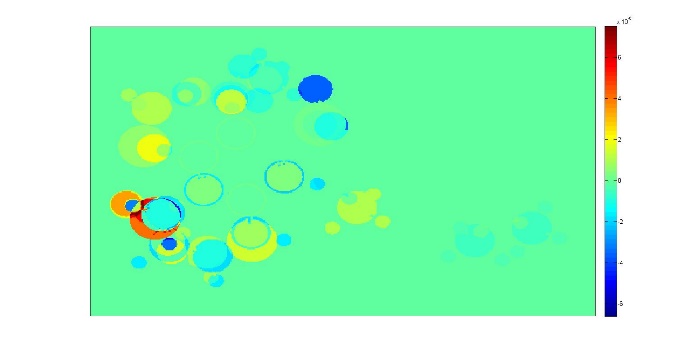 | 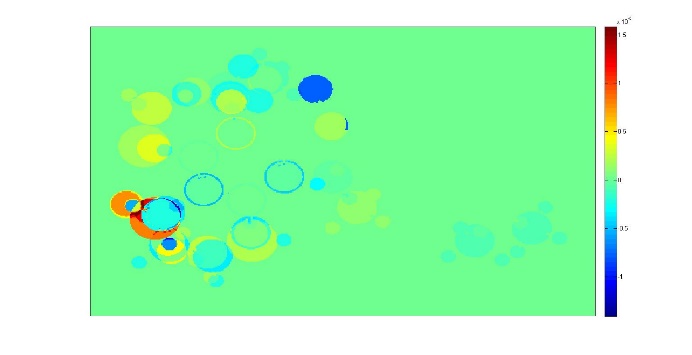 | 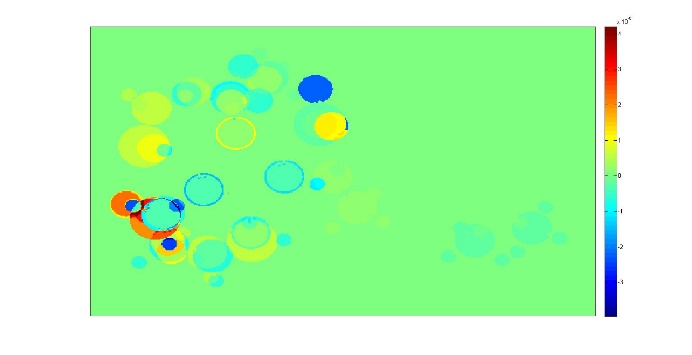 |
| Model 10 | 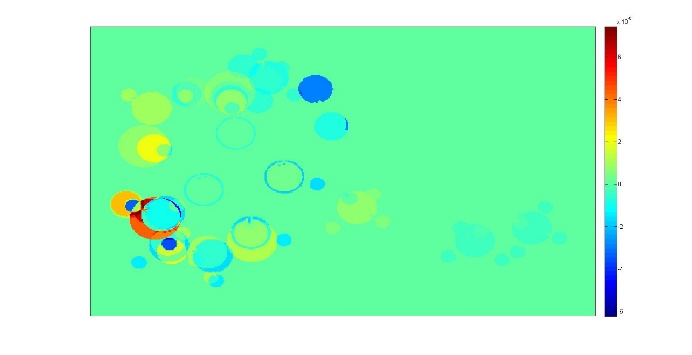 | 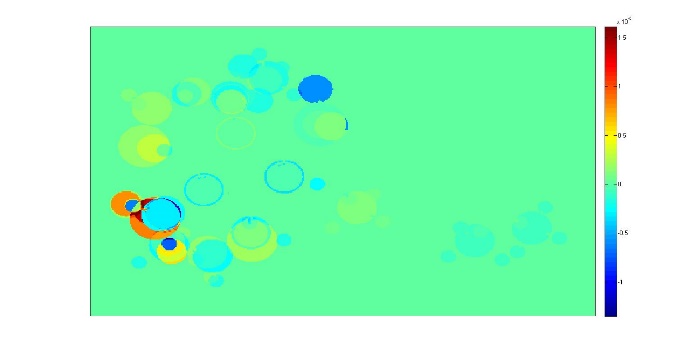 | 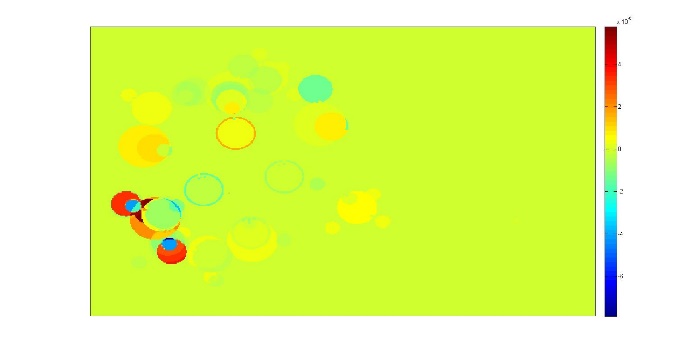 |

S.I. 10 - MIA plot’s: VIP

|  | Electronegativity | Van derWalls radius | r_VdW_/ε ratio |
| --- | --- | --- | --- |
| Model 1 | 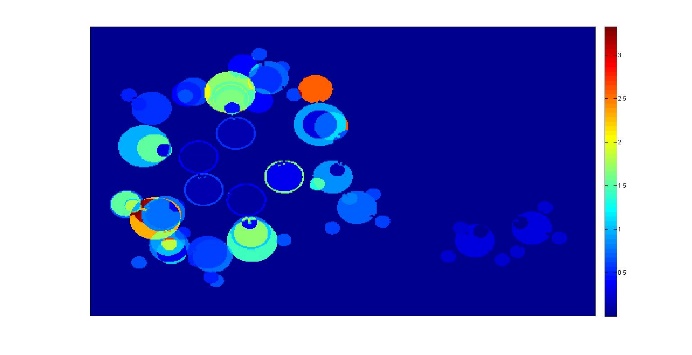 | 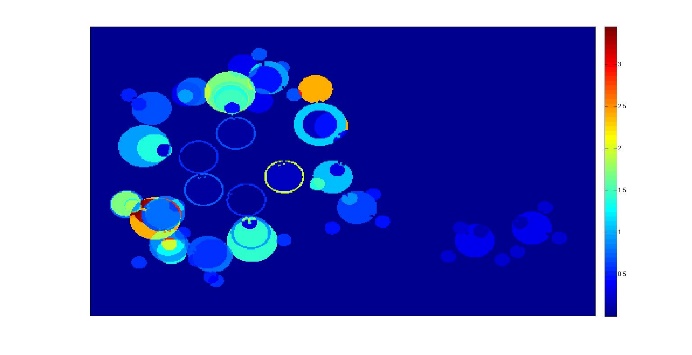 | 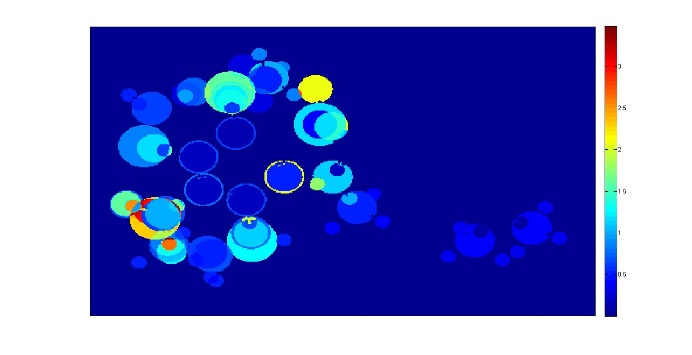 |
| Model 2 | 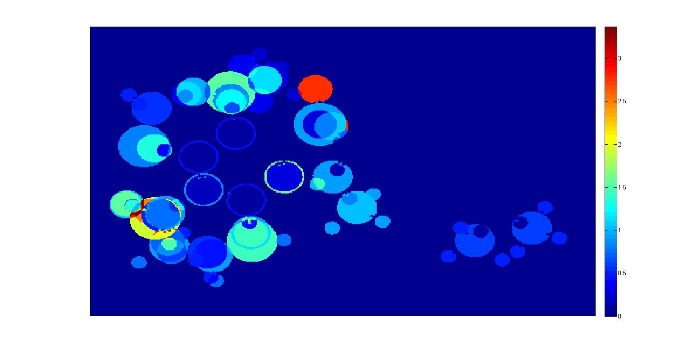 | 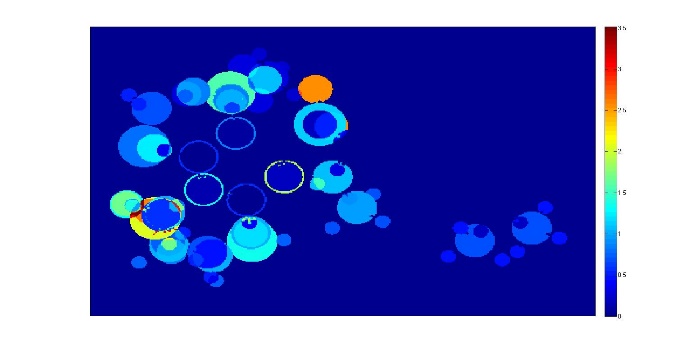 | 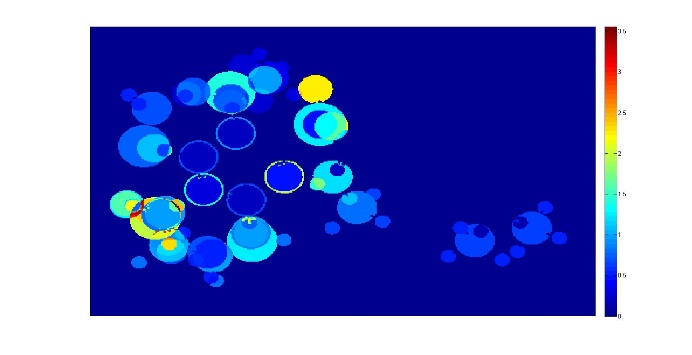 |
| Model 3 | 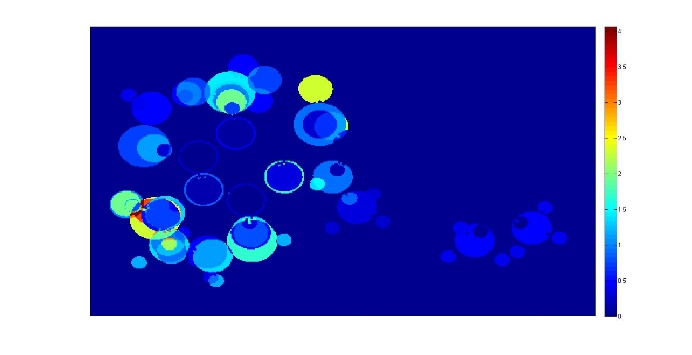 | 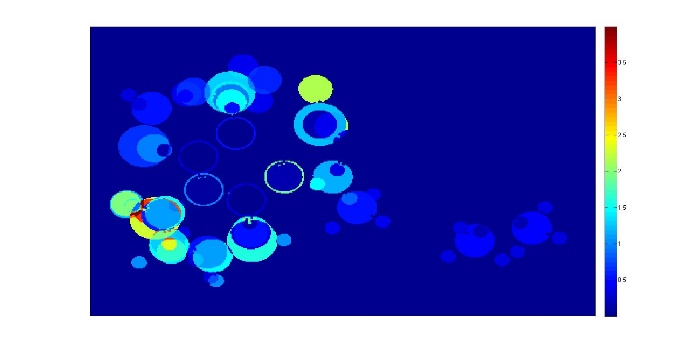 | 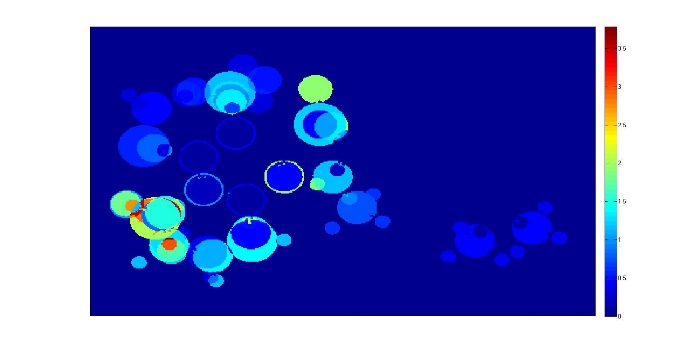 |
| Model 4 | 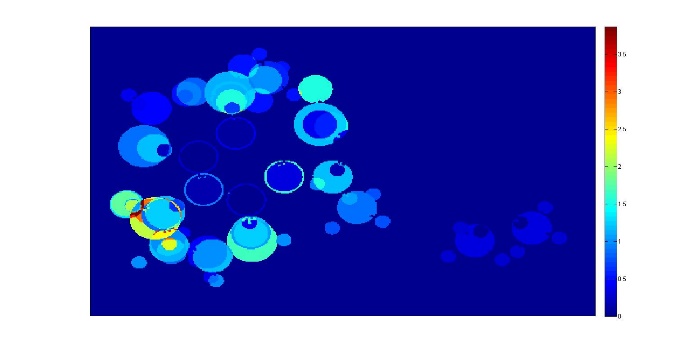 | 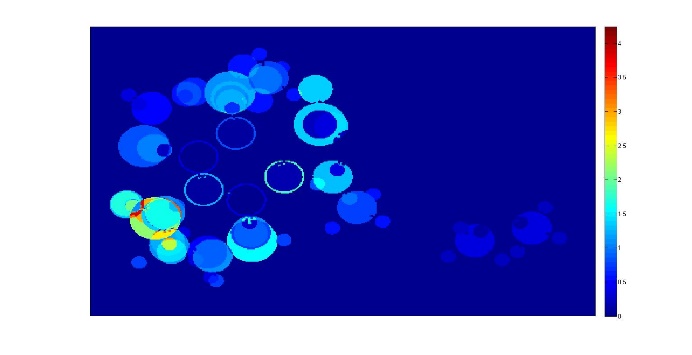 | 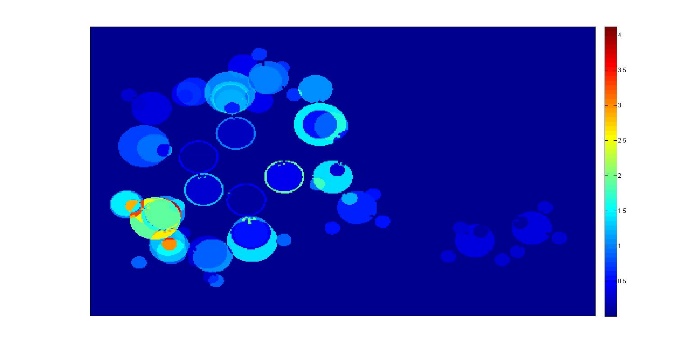 |
| Model 5 | 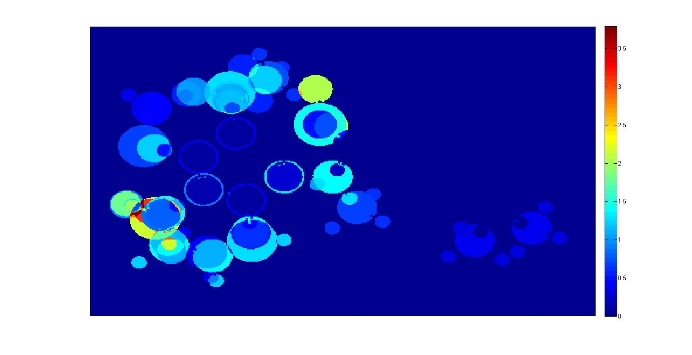 | 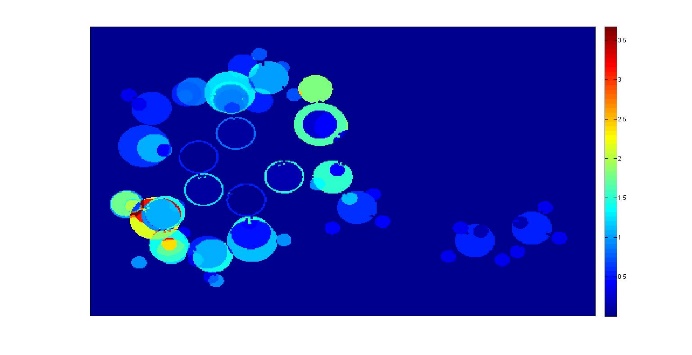 | 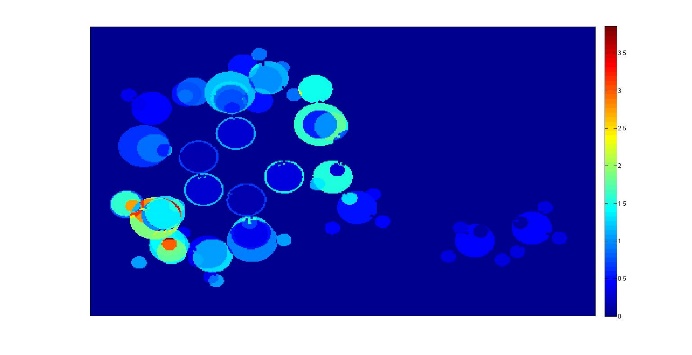 |
| Model 6 | 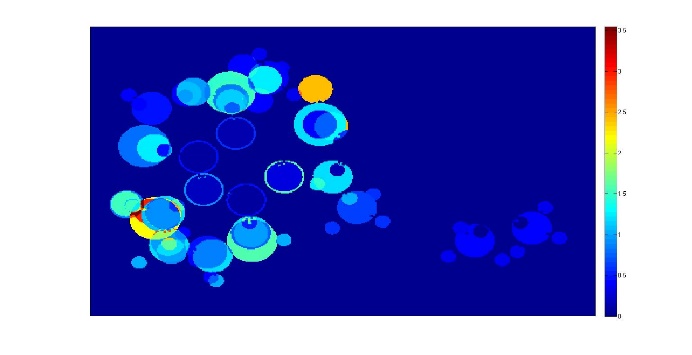 | 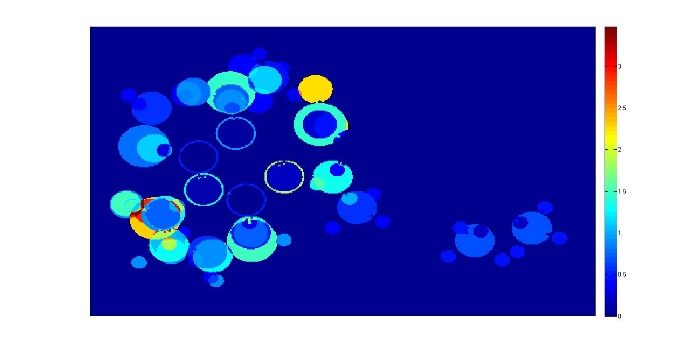 | 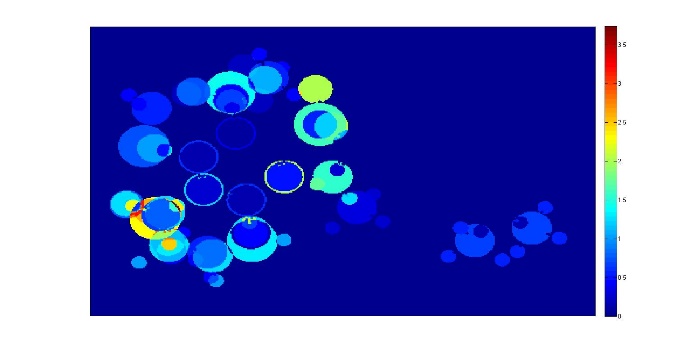 |
| Model 7 | 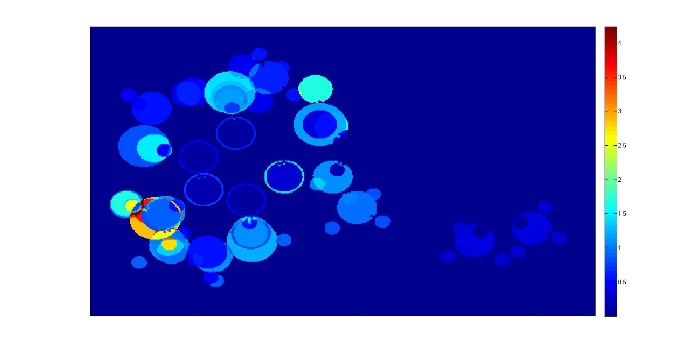 | 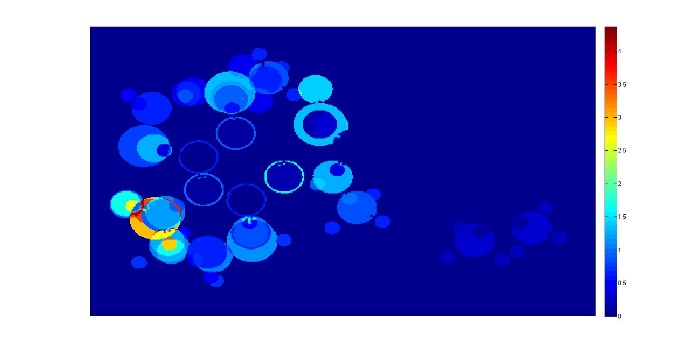 | 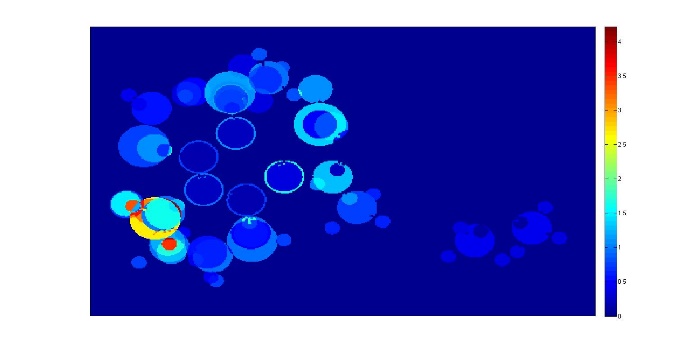 |
| Model 8 | 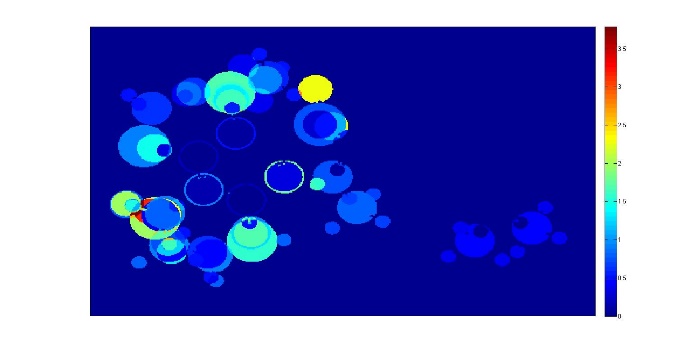 | 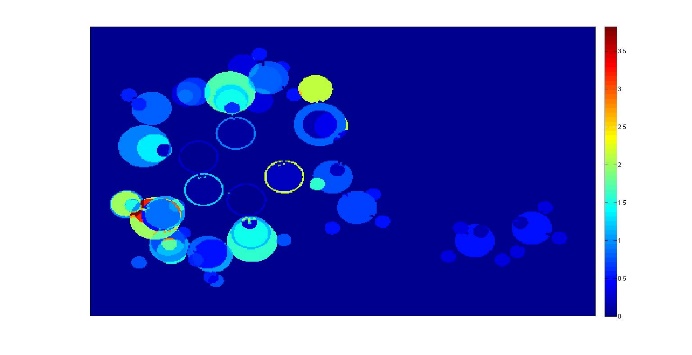 | 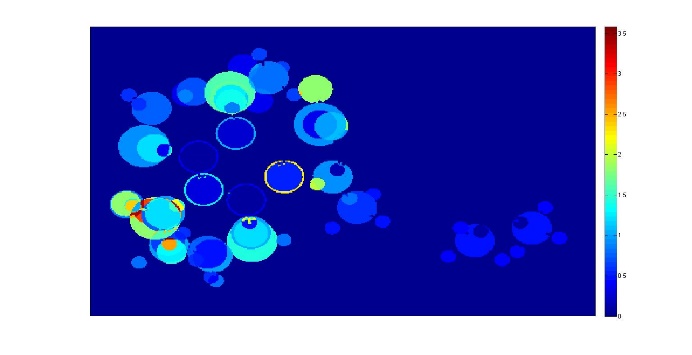 |
| Model 9 | 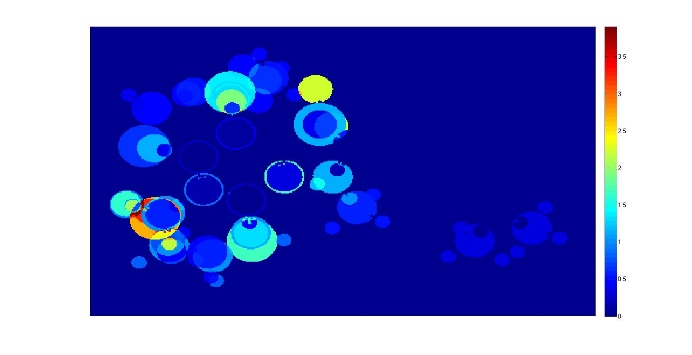 | 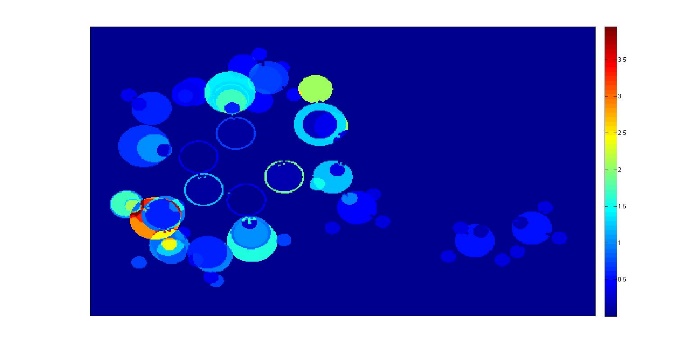 | 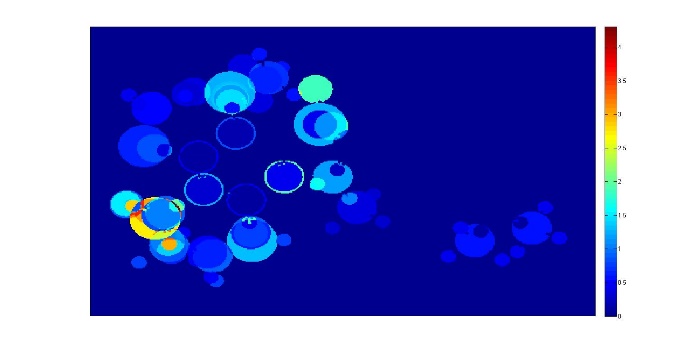 |
| Model 10 | 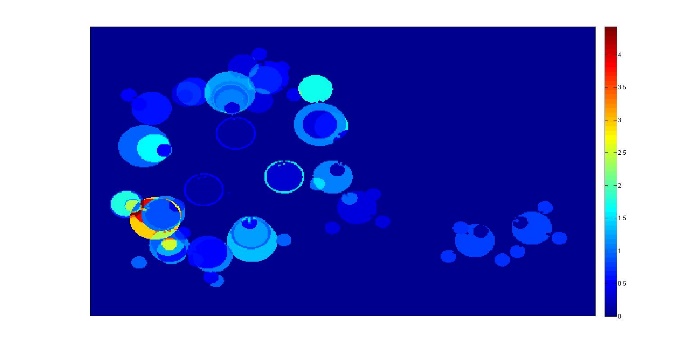 | 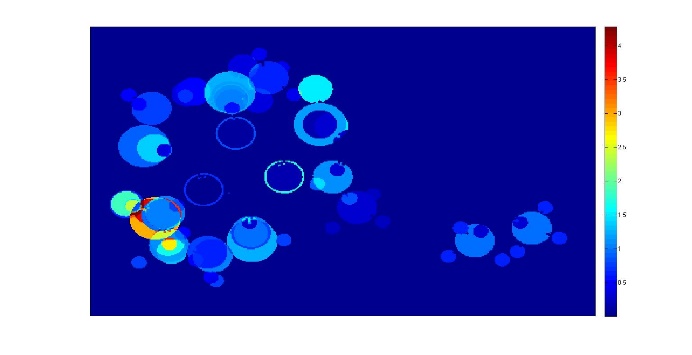 | 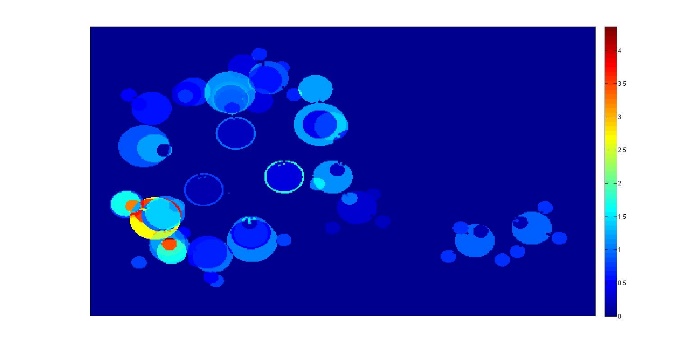 |

S.I. 11 - Activity prediction of proposed compounds based on electronegativity

| Samples | Model 1 | Model 2 | Model 3 | Model 4 | Model 5 | Model 6 | Model 7 | Model 8 | Model 9 | Model 10 | Mean | St. Dev. |
| --- | --- | --- | --- | --- | --- | --- | --- | --- | --- | --- | --- | --- |
| P1 | 7.8938 | 8.0737 | 7.9666 | 8.2699 | 8.0701 | 8.5068 | 8.3199 | 8.1560 | 8.1412 | 8.0607 | 8.1459 | 0.1795 |
| P2 | 7.8535 | 8.1686 | 7.9781 | 8.4586 | 8.1586 | 8.5619 | 8.3783 | 8.2343 | 8.1617 | 8.1990 | 8.2153 | 0.2113 |
| P3 | 7.5013 | 7.5050 | 7.6548 | 7.6820 | 7.6664 | 8.0647 | 7.6660 | 7.8254 | 7.6472 | 7.6751 | 7.6888 | 0.1609 |
| P4 | 7.5751 | 7.7627 | 7.8146 | 7.9923 | 7.8772 | 8.4179 | 7.9621 | 8.0463 | 7.8766 | 7.7854 | 7.9110 | 0.2228 |
| P5 | 7.8796 | 7.5998 | 7.9400 | 7.6156 | 7.8765 | 8.0926 | 7.8705 | 8.0216 | 7.8573 | 7.7517 | 7.8505 | 0.1584 |
| P6 | 7.5638 | 7.6565 | 7.7412 | 7.9030 | 7.7659 | 8.0030 | 7.8121 | 7.7069 | 7.8517 | 7.8751 | 7.7879 | 0.1285 |
| P7 | 7.5234 | 7.7514 | 7.7527 | 8.0917 | 7.8544 | 8.0582 | 7.8704 | 7.7852 | 7.8722 | 8.0134 | 7.8573 | 0.1696 |
| P8 | 7.9431 | 7.7524 | 8.0277 | 7.8383 | 7.9772 | 8.0323 | 8.0183 | 7.9042 | 8.0633 | 7.9532 | 7.9510 | 0.0970 |
| P9 | 7.9027 | 7.8473 | 8.0392 | 8.0271 | 8.0657 | 8.0875 | 8.0766 | 7.9825 | 8.0838 | 8.0915 | 8.0204 | 0.0846 |
| P10 | 7.2484 | 7.2724 | 7.3888 | 7.5106 | 7.4399 | 7.5729 | 7.3735 | 7.3729 | 7.4748 | 7.3948 | 7.4049 | 0.1000 |
| P11 | 7.3222 | 7.5301 | 7.5486 | 7.8209 | 7.6507 | 7.9262 | 7.6697 | 7.5938 | 7.7042 | 7.5051 | 7.6271 | 0.1699 |
| P12 | 7.2080 | 7.3673 | 7.4003 | 7.6993 | 7.5284 | 7.6281 | 7.4318 | 7.4512 | 7.4953 | 7.5330 | 7.4743 | 0.1380 |
| P13 | 7.6248 | 7.3672 | 7.6729 | 7.4437 | 7.6504 | 7.6013 | 7.5782 | 7.5676 | 7.6847 | 7.4714 | 7.5662 | 0.1059 |
| P14 | 7.5845 | 7.4621 | 7.6844 | 7.6325 | 7.7389 | 7.6564 | 7.6365 | 7.6459 | 7.7052 | 7.6096 | 7.6356 | 0.0759 |

S.I. 12 - Activity prediction of proposed compounds based on van der Waals radius

| Samples | Model 1 | Model 2 | Model 3 | Model 4 | Model 5 | Model 6 | Model 7 | Model 8 | Model 9 | Model 10 | Mean | St. Dev. |
| --- | --- | --- | --- | --- | --- | --- | --- | --- | --- | --- | --- | --- |
| P1 | 7.7703 | 8.2544 | 7.5528 | 8.1079 | 7.9741 | 8.4422 | 8.1771 | 8.3372 | 8.1040 | 7.8294 | 8.0549 | 0.2749 |
| P2 | 7.6783 | 8.2164 | 7.6143 | 8.1464 | 7.9638 | 8.4260 | 8.1232 | 8.1821 | 8.0513 | 7.7654 | 8.0167 | 0.2596 |
| P3 | 7.4281 | 7.4395 | 7.1803 | 7.5313 | 7.4523 | 7.7479 | 7.3822 | 7.9435 | 7.4729 | 7.3195 | 7.4897 | 0.2157 |
| P4 | 7.4422 | 7.5829 | 7.2533 | 7.6789 | 7.5803 | 7.9191 | 7.5040 | 8.0551 | 7.5985 | 7.3692 | 7.5984 | 0.2411 |
| P5 | 7.8551 | 7.6438 | 7.6768 | 7.5552 | 7.6947 | 7.8671 | 7.5999 | 8.2835 | 7.6010 | 7.6590 | 7.7436 | 0.2159 |
| P6 | 7.4767 | 7.7520 | 7.4581 | 7.8213 | 7.7138 | 7.8891 | 7.7593 | 7.9562 | 7.8949 | 7.6128 | 7.7334 | 0.1717 |
| P7 | 7.3848 | 7.7140 | 7.5196 | 7.8599 | 7.7036 | 7.8729 | 7.7054 | 7.8011 | 7.8422 | 7.5489 | 7.6952 | 0.1635 |
| P8 | 7.9047 | 7.9576 | 7.9559 | 7.8468 | 7.9575 | 8.0097 | 7.9787 | 8.2974 | 8.0248 | 7.9539 | 7.9887 | 0.1195 |
| P9 | 7.8127 | 7.9196 | 8.0174 | 7.8853 | 7.9472 | 7.9934 | 7.9248 | 8.1423 | 7.9721 | 7.8899 | 7.9505 | 0.0895 |
| P10 | 7.2002 | 7.4490 | 7.2718 | 7.6656 | 7.5488 | 7.6708 | 7.4715 | 7.6581 | 7.5866 | 7.3741 | 7.4896 | 0.1672 |
| P11 | 7.2143 | 7.5923 | 7.3448 | 7.8132 | 7.6768 | 7.8420 | 7.5933 | 7.7697 | 7.7123 | 7.4238 | 7.5983 | 0.2098 |
| P12 | 7.1082 | 7.4110 | 7.3332 | 7.7041 | 7.5386 | 7.6546 | 7.4176 | 7.5030 | 7.5340 | 7.3101 | 7.4514 | 0.1753 |
| P13 | 7.6243 | 7.6527 | 7.7657 | 7.6889 | 7.7909 | 7.7889 | 7.6889 | 7.9960 | 7.7136 | 7.7123 | 7.7422 | 0.1048 |
| P14 | 7.5324 | 7.6147 | 7.8272 | 7.7274 | 7.7806 | 7.7727 | 7.6350 | 7.8409 | 7.6609 | 7.6483 | 7.7040 | 0.1012 |

S.I. 13 - Predicted activity of proposed compounds derived from van der Waals radius/electronegativity ratio

| Samples | Model 1 | Model 2 | Model 3 | Model 4 | Model 5 | Model 6 | Model 7 | Model 8 | Model 9 | Model 10 | Mean | St. Dev. |
| --- | --- | --- | --- | --- | --- | --- | --- | --- | --- | --- | --- | --- |
| P1 | 7.6578 | 8.1839 | 7.9125 | 7.7722 | 7.9219 | 7.9615 | 8.1101 | 8.6504 | 7.9556 | 7.9781 | 8.0104 | 0.2697 |
| P2 | 7.5254 | 8.1380 | 7.8200 | 7.7435 | 7.9091 | 8.1361 | 8.0268 | 8.2988 | 7.8873 | 7.8678 | 7.9353 | 0.2230 |
| P3 | 7.0769 | 7.1348 | 7.1741 | 6.9543 | 7.4356 | 7.0450 | 7.1991 | 7.9904 | 7.1717 | 7.5675 | 7.2750 | 0.3099 |
| P4 | 7.0519 | 7.1588 | 7.1549 | 6.9750 | 7.4348 | 7.1164 | 7.1981 | 7.9721 | 7.2047 | 7.5647 | 7.2831 | 0.2979 |
| P5 | 7.5570 | 7.3264 | 7.8015 | 6.9555 | 7.6467 | 7.1922 | 7.3178 | 8.4010 | 7.3341 | 7.7625 | 7.5295 | 0.4041 |
| P6 | 7.2312 | 7.5916 | 7.3776 | 7.5726 | 7.4954 | 7.5698 | 7.6608 | 8.0130 | 7.6771 | 7.6659 | 7.5855 | 0.2056 |
| P7 | 7.0989 | 7.5458 | 7.2851 | 7.5439 | 7.4827 | 7.7443 | 7.5775 | 7.6614 | 7.6088 | 7.5557 | 7.5104 | 0.1877 |
| P8 | 7.7120 | 7.7845 | 8.0060 | 7.5753 | 7.7072 | 7.7181 | 7.7809 | 8.4245 | 7.8412 | 7.8617 | 7.8411 | 0.2346 |
| P9 | 7.5797 | 7.7386 | 7.9135 | 7.5466 | 7.6945 | 7.8927 | 7.6976 | 8.0729 | 7.7728 | 7.7514 | 7.7660 | 0.1584 |
| P10 | 7.0549 | 7.4843 | 7.1203 | 7.6557 | 7.5452 | 7.5283 | 7.6627 | 7.8689 | 7.6633 | 7.5874 | 7.5171 | 0.2502 |
| P11 | 7.0299 | 7.5084 | 7.1012 | 7.6764 | 7.5444 | 7.5997 | 7.6617 | 7.8506 | 7.6964 | 7.5845 | 7.5253 | 0.2607 |
| P12 | 6.9226 | 7.4385 | 7.0278 | 7.6270 | 7.5325 | 7.7029 | 7.5794 | 7.5174 | 7.5950 | 7.4771 | 7.4420 | 0.2584 |
| P13 | 7.5320 | 7.6750 | 7.7448 | 7.6559 | 7.7560 | 7.6734 | 7.7813 | 8.2768 | 7.8240 | 7.7813 | 7.7701 | 0.1969 |
| P14 | 7.3997 | 7.6292 | 7.6523 | 7.6272 | 7.7433 | 7.8480 | 7.6979 | 7.9252 | 7.7557 | 7.6711 | 7.6950 | 0.1418 |

S.I. 14 - Ranking of compounds according to pKi, with corresponding Log P values

| Compound | Log P | pKi |
| --- | --- | --- |
| P1 | 3.23 | 8.0704 |
| P2 | 3.83 | 8.0558 |
| P8 | 3.25 | 7.9269 |
| P9 | 3.86 | 7.9123 |
| P5 | 3.47 | 7.7079 |
| P6 | 2.01 | 7.7023 |
| Mesotrione | 0.42 | 7.6990 |
| P13 | 1.58 | 7.6928 |
| P7 | 2.62 | 7.6876 |
| P14 | 2.19 | 7.6782 |
| B²23 | 3.26 | 7.6021 |
| P4 | 2.32 | 7.5975 |
| P11 | 0.43 | 7.5836 |
| P3 | 2.23 | 7.4845 |
| P10 | 0.34 | 7.4706 |
| P12 | 0.94 | 7.4559 |
| B²27 | 1.41 | 7.3468 |
| B²28 | 2.06 | 7.3010 |
| B¹5 | 2.42 | 7.2840 |
| B²24 | 3.92 | 7.2441 |
| B¹15 | 2.31 | 7.1739 |
| B²21 | 1.44 | 7.1549 |
| A¹7 | 2.88 | 7.1308 |
| B¹16 | 2.96 | 7.1079 |
| B²25 | 1.76 | 7.0757 |
| B¹6 | 3.07 | 7.0506 |
| B²22 | 2.09 | 7.0315 |
| B²26 | 2.42 | 7.0269 |
| A¹9 | 2.16 | 7.0223 |
| A¹11 | 3.48 | 6.9914 |
| A¹3 | 2.60 | 6.9547 |
| A¹12 | 4.14 | 6.9547 |
| B¹17 | 2.52 | 6.9431 |
| B¹7 | 1.70 | 6.9318 |
| A¹4 | 3.25 | 6.9101 |
| A¹10 | 2.18 | 6.8327 |
| B²19 | 1.66 | 6.8125 |
| B¹18 | 3.18 | 6.8041 |
| B¹8 | 2.35 | 6.6968 |
| A¹13 | 2.51 | 6.6253 |
| A¹1 | 2.23 | 6.5952 |
| B²20 | 2.31 | 6.5622 |
| B¹14 | 3.18 | 6.5346 |
| B¹12 | 2.24 | 6.5157 |
| A¹8 | 3.53 | 6.4572 |
| B¹13 | 2.52 | 6.4461 |
| B¹11 | 1.59 | 6.3737 |
| A¹5 | 2.23 | 6.2790 |
| A¹2 | 2.88 | 6.2411 |
| A¹6 | 2.89 | 6.1959 |
| A¹14 | 3.17 | 6.1772 |
| A²15 | 3.38 | 6.0670 |
| A²16 | 3.27 | 5.8386 |
| B¹3 | 2.19 | 5.7762 |
| A²17 | 3.92 | 5.6778 |
| B¹4 | 2.84 | 5.5405 |
| B¹2 | 0.18 | 5.5358 |
| B¹1 | 0.63 | 5.3736 |

S.I. 15 - Docking output: mesotrione in the crystallographic HPPD structure (PDB 5YWG)

| Atom | | Energies | | | | Partial  charge | Coordinates | | |
| --- | --- | --- | --- | --- | --- | --- | --- | --- | --- |
| Number | Type | Total | vdW+Hbond | Electrostatic | Desolvation |  | x | y | z |
| 1 | S | 0.4906 | -0.5441 | 0.2390 | 0.7958 | 1.251 | 24.503 | -2.615 | -31.638 |
| 2 | OA | -0.1494 | -0.3692 | -0.1193 | 0.3390 | -0.578 | 23.436 | -1.651 | -32.160 |
| 3 | OA | -0.1605 | -0.5631 | -0.0659 | 0.4685 | -0.578 | 25.026 | -3.363 | -32.834 |
| 4 | C | -0.3713 | -0.3768 | -0.0018 | 0.0073 | -0.013 | 25.738 | -1.477 | -30.851 |
| 5 | A | -0.3770 | -0.3957 | -0.0164 | 0.0351 | -0.051 | 24.060 | -3.864 | -30.870 |
| 6 | A | -0.4451 | -0.4514 | -0.0025 | 0.0088 | -0.011 | 24.577 | -5.094 | -31.257 |
| 7 | A | -0.4231 | -0.4448 | 0.0094 | 0.0122 | 0.015 | 24.101 | -6.249 | -30.663 |
| 8 | A | -0.2494 | -0.3938 | 0.0905 | 0.0539 | 0.072 | 23.115 | -6.128 | -29.699 |
| 9 | A | 0.0181 | -0.2754 | 0.1667 | 0.1267 | 0.183 | 22.650 | -4.882 | -29.362 |
| 10 | A | -0.2635 | -0.3120 | 0.0215 | 0.0271 | 0.043 | 23.134 | -3.729 | -29.902 |
| 11 | C | 0.3055 | -0.4693 | 0.6112 | 0.1637 | 0.203 | 22.524 | -7.276 | -29.043 |
| 12 | OA | -1.0809 | 0.5668 | -1.9179 | 0.2703 | -0.288 | 21.347 | -7.475 | -29.415 |
| 13 | C | -0.1478 | -0.4262 | 0.2054 | 0.0731 | 0.091 | 23.315 | -8.101 | -28.107 |
| 14 | C | -0.2961 | -0.4102 | 0.0669 | 0.0472 | 0.063 | 24.559 | -7.532 | -27.572 |
| 15 | C | -0.3792 | -0.4799 | 0.0504 | 0.0503 | 0.062 | 25.209 | -8.080 | -26.291 |
| 16 | C | -0.4884 | -0.5137 | 0.0125 | 0.0128 | 0.015 | 25.161 | -9.584 | -26.414 |
| 17 | C | -0.3536 | -0.5391 | 0.1111 | 0.0744 | 0.081 | 23.693 | -9.981 | -26.529 |
| 18 | C | 0.1197 | -0.5228 | 0.4920 | 0.1505 | 0.169 | 22.894 | -9.275 | -27.666 |
| 19 | OA | -1.6112 | 0.2309 | -2.1394 | 0.2973 | -0.294 | 21.679 | -9.925 | -28.182 |
| 20 | OA | -0.4159 | -0.4216 | -0.2500 | 0.2557 | -0.364 | 25.289 | -6.197 | -27.871 |
| 21 | HD | 0.2420 | -0.0335 | 0.1156 | 0.1599 | 0.218 | 25.545 | -6.157 | -28.888 |
| 22 | N | 0.6440 | -0.4156 | 0.6430 | 0.4165 | 0.572 | 21.797 | -4.773 | -28.551 |
| 23 | OA | -0.8817 | -0.5090 | -0.7515 | 0.3788 | -0.430 | 20.749 | -5.325 | -28.631 |
| 24 | OA | -0.5135 | -0.4135 | -0.3846 | 0.2846 | -0.430 | 22.110 | -4.377 | -27.510 |
|  | Total | -6.7878 | -8.4832 | -2.8141 | 4.5096 | 0.001 |  |  |  |

Estimated Free Energy of Binding = -6.36 kcal/mol [=(1)+(2)+(3)-(4)]

(1) Final Intermolecular Energy = -6.79 kcal/mol

vdW + Hbond + desolv Energy = -3.97 kcal/mol

Electrostatic Energy = -2.81 kcal/mol

(2) Final Total Internal Energy = -1.06 kcal/mol

(3) Torsional Free Energy = +1.49 kcal/mol

(4) Unbound System's Energy = +0.00 kcal/mol

S.I. 16 - Docking output: mesotrione best pose (pose 69, lowest RMSD)

| **Coordinates** | | | **Energies** | | **Partial charge** | **Atom type** |
| --- | --- | --- | --- | --- | --- | --- |
| x | y | z | vdW+H-bond | Electrostatic |  |  |
| 24.422 | -2.346 | -31.348 | 0.24 | 0.25 | 1.251 | S |
| 23.330 | -1.278 | -31.263 | -0.02 | -0.13 | -0.578 | OA |
| 24.487 | -2.779 | -32.787 | -0.05 | -0.09 | -0.578 | OA |
| 25.930 | -1.441 | -30.756 | -0.34 | 0.00 | -0.013 | C |
| 24.137 | -3.743 | -30.790 | -0.35 | -0.02 | -0.051 | A |
| 24.689 | -4.839 | -31.442 | -0.46 | 0.00 | -0.011 | A |
| 24.348 | -6.116 | -31.035 | -0.39 | 0.01 | 0.015 | A |
| 23.457 | -6.249 | -29.983 | -0.37 | 0.08 | 0.072 | A |
| 22.950 | -5.124 | -29.382 | -0.20 | 0.17 | 0.183 | A |
| 23.305 | -3.858 | -29.738 | -0.29 | 0.02 | 0.043 | A |
| 23.007 | -7.539 | -29.504 | -0.35 | 0.49 | 0.203 | C |
| 22.120 | -8.029 | -30.236 | -0.39 | -1.34 | -0.288 | OA |
| 23.628 | -8.162 | -28.317 | -0.36 | 0.18 | 0.091 | C |
| 24.709 | -7.430 | -27.643 | -0.37 | 0.06 | 0.063 | C |
| 25.108 | -7.742 | -26.191 | -0.43 | 0.05 | 0.062 | C |
| 25.156 | -9.247 | -26.090 | -0.49 | 0.01 | 0.015 | C |
| 23.763 | -9.767 | -26.427 | -0.46 | 0.11 | 0.081 | C |
| 23.181 | -9.291 | -27.792 | -0.37 | 0.43 | 0.169 | C |
| 22.128 | -10.097 | -28.429 | -0.29 | -1.64 | -0.294 | OA |
| 25.421 | -6.099 | -27.994 | -0.15 | -0.24 | -0.364 | OA |
| 24.793 | -5.291 | -27.759 | 0.13 | 0.14 | 0.218 | HD |
| 22.177 | -5.231 | -28.494 | 0.02 | 0.73 | 0.572 | N |
| 21.059 | -5.600 | -28.646 | -0.18 | -0.84 | -0.430 | OA |
| 22.611 | -5.281 | -27.423 | -0.09 | -0.48 | -0.430 | OA |

Estimated Free Energy of Binding = -6.56 kcal/mol [=(1)+(2)+(3)-(4)]

(1) Final Intermolecular Energy = -8.05 kcal/mol

vdW + Hbond + desolv Energy = -6.01 kcal/mol

Electrostatic Energy = -2.05 kcal/mol

(2) Final Total Internal Energy = -2.15 kcal/mol

(3) Torsional Free Energy = +1.49 kcal/mol

(4) Unbound System's Energy [=(2)] = -2.15 kcal/mol

S.I. 17 - Docking output: B9 compound lowest-energy pose

| **Coordinates** | | | **Energies** | | **Partial charge** | **Atom type** |
| --- | --- | --- | --- | --- | --- | --- |
| x | y | z | vdW+H-bond | Electrostatic |  |  |
| 23.065 | -3.867 | -28.777 | -0.27 | 0.03 | 0.057 | A |
| 23.334 | -3.272 | -30.003 | -0.31 | 0.01 | 0.019 | A |
| 22.075 | -3.327 | -27.948 | -0.23 | 0.12 | 0.187 | A |
| 21.371 | -2.206 | -28.366 | -0.31 | 0.03 | 0.055 | A |
| 21.643 | -1.620 | -29.590 | -0.35 | 0.00 | 0.004 | A |
| 22.627 | -2.150 | -30.411 | -0.31 | 0.00 | 0.002 | A |
| 23.939 | -5.354 | -28.351 | -0.36 | -0.09 | -0.114 | SA |
| 23.841 | -6.397 | -29.820 | -0.38 | 0.15 | 0.156 | C |
| 23.158 | -7.735 | -29.496 | -0.36 | 0.42 | 0.178 | C |
| 22.121 | -8.038 | -30.098 | -0.29 | -1.46 | -0.293 | OA |
| 23.813 | -8.777 | -28.607 | -0.37 | 0.17 | 0.087 | C |
| 25.162 | -8.825 | -28.502 | -0.39 | 0.06 | 0.063 | C |
| 25.841 | -9.853 | -27.616 | -0.46 | 0.05 | 0.062 | C |
| 24.964 | -10.213 | -26.418 | -0.53 | 0.01 | 0.015 | C |
| 23.648 | -10.764 | -26.945 | -0.45 | 0.12 | 0.081 | C |
| 2.983 | -9.744 | -27.810 | -0.41 | 0.47 | 0.168 | C |
| 21.750 | -9.714 | -27.850 | -0.13 | -1.78 | -0.294 | OA |
| 26.001 | -7.905 | -29.184 | -0.35 | -0.20 | -0.364 | OA |
| 25.871 | -7.962 | -30.167 | -0.41 | 0.04 | 0.218 | HD |
| 21.722 | -3.869 | -26.698 | -0.03 | 0.44 | 0.572 | N |
| 22.119 | -3.164 | -25.854 | -0.59 | -0.29 | -0.430 | OA |
| 21.104 | -4.819 | -26.963 | -0.24 | -0.53 | -0.430 | OA |

Estimated Free Energy of Binding = -7.99 kcal/mol [=(1)+(2)+(3)-(4)]

(1) Final Intermolecular Energy = -9.78 kcal/mol

vdW + Hbond + desolv Energy = -7.55 kcal/mol

Electrostatic Energy = -2.24 kcal/mol

(2) Final Total Internal Energy = -0.41 kcal/mol

(3) Torsional Free Energy = +1.79 kcal/mol

(4) Unbound System's Energy [=(2)] = -0.41 kcal/mol

S.I. 18 - Docking output: B1 compound lowest-energy pose

| **Coordinates** | | | **Energies** | | **Partial charge** | **Atom type** |
| --- | --- | --- | --- | --- | --- | --- |
| x | y | z | vdW+H-bond | Electrostatic |  |  |
| 24.368 | -9.608 | -26.912 | -0.37 | -0.06 | -0.054 | A |
| 24.367 | -10.492 | -25.845 | -0.46 | -0.01 | -0.012 | A |
| 25.457 | -9.525 | -27.761 | -0.40 | -0.01 | -0.012 | A |
| 26.561 | -10.340 | -27.547 | -0.46 | 0.00 | 0.002 | A |
| 26.567 | -11.231 | -26.479 | -0.46 | -0.02 | -0.051 | A |
| 25.469 | -11.303 | -25.628 | -0.47 | 0.00 | 0.002 | A |
| 27.759 | -12.110 | -26.242 | -0.58 | 0.00 | 0.044 | C |
| 23.000 | -8.553 | -27.181 | 0.51 | 2.89 | 1.262 | S |
| 22.338 | -8.428 | -25.813 | -0.53 | -1.10 | -0.578 | OA |
| 22.087 | -9.474 | -27.982 | 0.04 | -3.18 | -0.578 | OA |
| 23.652 | -6.953 | -28.013 | -0.32 | 0.11 | 0.076 | C |
| 22.859 | -6.418 | -29.220 | -0.34 | 0.27 | 0.160 | C |
| 21.714 | -6.942 | -29.548 | -0.34 | -1.05 | -0.295 | OA |
| 23.330 | -5.154 | -29.919 | -0.34 | 0.07 | 0.085 | C |
| 24.050 | -5.242 | -31.054 | -0.47 | 0.03 | 0.063 | C |
| 24.528 | -4.013 | -31.795 | -0.50 | 0.01 | 0.062 | C |
| 23.633 | -2.810 | -31.500 | -0.38 | 0.00 | 0.015 | C |
| 23.580 | -2.608 | -29.994 | -0.30 | 0.03 | 0.081 | C |
| 22.989 | -3.795 | -29.378 | -0.26 | 0.09 | 0.168 | C |
| 22.168 | -3.633 | -28.457 | -0.20 | -0.20 | -0.294 | OA |
| 24.380 | -6.470 | -31.551 | -0.67 | -0.11 | -0.364 | OA |
| 25.096 | -6.360 | -32.312 | -0.42 | -0.06 | 0.218 | HD |

Estimated Free Energy of Binding = -8.56 kcal/mol [=(1)+(2)+(3)-(4)]

(1) Final Intermolecular Energy = -10.05 kcal/mol

vdW + Hbond + desolv Energy = -7.73 kcal/mol

Electrostatic Energy = -2.32 kcal/mol

(2) Final Total Internal Energy = -0.05 kcal/mol

(3) Torsional Free Energy = +1.49 kcal/mol

(4) Unbound System's Energy [=(2)] = -0.05 kcal/mol

S.I. 19 - Docking output: A7 compound lowest-energy pose

| **Coordinates** | | | **Energies** | | **Partial charge** | **Atom type** |
| --- | --- | --- | --- | --- | --- | --- |
| x | y | z | vdW+H-bond | Electrostatic |  |  |
| 24.626 | -4.983 | -31.759 | -0.35 | 0.02 | 0.135 | A |
| 24.355 | -4.176 | -30.686 | -0.38 | 0.01 | 0.022 | A |
| 24.639 | -2.818 | -30.688 | -0.30 | 0.01 | 0.032 | A |
| 25.218 | -2.287 | -31.839 | -0.40 | 0.01 | 0.041 | A |
| 25.498 | -3.106 | -32.941 | -0.53 | 0.00 | 0.024 | A |
| 25.202 | -4.470 | -32.910 | -0.48 | 0.00 | 0.049 | A |
| 23.796 | -4.995 | -29.728 | -0.32 | 0.04 | 0.063 | A |
| 24.270 | -6.204 | -31.501 | -0.61 | -0.16 | -0.453 | OA |
| 23.757 | -6.270 | -30.289 | -0.32 | 0.15 | 0.176 | A |
| 25.597 | -0.597 | -31.905 | -0.48 | -0.01 | -0.084 | Cl |
| 23.227 | -7.616 | -29.628 | -0.31 | 0.49 | 0.234 | C |
| 22.117 | -7.978 | -30.024 | -0.31 | -1.42 | -0.285 | OA |
| 23.964 | -8.551 | -28.538 | -0.36 | 0.16 | 0.095 | C |
| 25.327 | -8.589 | -28.434 | -0.45 | 0.06 | 0.064 | C |
| 26.031 | -9.475 | -27.420 | -0.44 | 0.05 | 0.062 | C |
| 25.244 | -10.728 | -27.120 | -0.48 | 0.01 | 0.015 | C |
| 23.907 | -10.294 | -26.586 | -0.47 | 0.10 | 0.081 | C |
| 23.206 | -9.454 | -27.602 | -0.38 | 0.40 | 0.169 | C |
| 21.997 | -9.590 | -27.699 | -0.28 | -1.45 | -0.294 | OA |
| 26.184 | -7.832 | -29.222 | -0.35 | -0.19 | -0.364 | OA |
| 25.772 | -7.723 | -30.182 | -0.35 | 0.04 | 0.218 | HD |

Estimated Free Energy of Binding = -9.12 kcal/mol [=(1)+(2)+(3)-(4)]

(1) Final Intermolecular Energy = -10.02 kcal/mol

vdW + Hbond + desolv Energy = -8.33 kcal/mol

Electrostatic Energy = -1.68 kcal/mol

(2) Final Total Internal Energy = -0.63 kcal/mol

(3) Torsional Free Energy = +0.89 kcal/mol

(4) Unbound System's Energy [=(2)] = -0.63 kcal/mol

S.I. 20 - Docking output: P1 compound lowest-energy pose

| **Coordinates** | | | **Energies** | | **Partial charge** | **Atom type** |
| --- | --- | --- | --- | --- | --- | --- |
| x | y | z | vdW+H-bond | Electrostatic |  |  |
| 24.257 | -2.831 | -30.217 | -0.23 | 0.04 | 0.108 | A |
| 24.067 | -4.180 | -30.420 | -0.34 | 0.02 | 0.044 | A |
| 24.581 | -4.874 | -31.481 | -0.40 | 0.02 | 0.104 | A |
| 24.272 | -6.125 | -31.401 | -0.79 | -0.12 | -0.304 | OA |
| 23.582 | -6.301 | -30.301 | -0.34 | 0.11 | 0.135 | A |
| 25.331 | -4.237 | -32.447 | -0.50 | 0.00 | 0.050 | A |
| 25.541 | -2.868 | -32.277 | -0.43 | 0.02 | 0.108 | A |
| 23.377 | -5.085 | -29.657 | -0.34 | 0.01 | 0.008 | A |
| 22.567 | -4.818 | -28.380 | -0.37 | 0.02 | 0.033 | C |
| 25.025 | -2.160 | -31.186 | -0.30 | 0.03 | 0.102 | A |
| 25.424 | -0.449 | -31.169 | -0.42 | -0.02 | -0.078 | Cl |
| 23.080 | -7.621 | -29.782 | -0.32 | 0.43 | 0.222 | C |
| 22.083 | -8.110 | -30.319 | -0.35 | -1.26 | -0.284 | OA |
| 23.944 | -8.507 | -28.801 | -0.35 | 0.12 | 0.093 | C |
| 25.299 | -8.375 | -28.657 | -0.42 | 0.05 | 0.073 | C |
| 26.061 | -9.239 | -27.675 | -0.40 | 0.04 | 0.051 | C |
| 25.197 | -9.633 | -26.489 | -0.49 | 0.01 | 0.015 | C |
| 23.969 | -10.379 | -26.958 | -0.46 | 0.06 | 0.070 | C |
| 23.227 | -9.557 | -27.941 | -0.37 | 0.33 | 0.178 | C |
| 22.008 | -9.752 | -28.037 | -0.24 | -1.19 | -0.291 | OA |
| 26.106 | -7.478 | -29.399 | -0.33 | -0.16 | -0.363 | OA |
| 25.715 | -7.375 | -30.300 | -0.38 | 0.03 | 0.217 | HD |
| 23.669 | -2.324 | -29.035 | -0.13 | -0.12 | -0.327 | OA |
| 22.457 | -1.650 | -29.167 | -0.22 | 0.06 | 0.181 | C |
| 26.285 | -2.156 | -33.198 | -0.32 | -0.05 | -0.327 | OA |
| 25.462 | -1.651 | -34.263 | -0.36 | 0.03 | 0.181 | C |

Estimated Free Energy of Binding = -9.57 kcal/mol [=(1)+(2)+(3)-(4)]

(1) Final Intermolecular Energy = -11.06 kcal/mol

vdW + Hbond + desolv Energy = -9.58 kcal/mol

Electrostatic Energy = -1.48 kcal/mol

(2) Final Total Internal Energy = -1.39 kcal/mol

(3) Torsional Free Energy = +1.49 kcal/mol

(4) Unbound System's Energy [=(2)] = -1.39 kcal/mol

S.I. 21 - Docking output: P2 compound lowest-energy pose

| **Coordinates** | | | **Energies** | | **Partial charge** | **Atom type** |
| --- | --- | --- | --- | --- | --- | --- |
| x | y | z | vdW+H-bond | Electrostatic |  |  |
| 23.417 | -3.118 | -29.044 | -0.22 | 0.04 | 0.108 | A |
| 23.938 | -2.156 | -29.940 | -0.22 | 0.03 | 0.103 | A |
| 24.463 | -2.522 | -31.187 | -0.29 | 0.03 | 0.119 | A |
| 24.486 | -3.867 | -31.590 | -0.39 | 0.03 | 0.104 | A |
| 23.376 | -6.607 | -29.848 | -0.33 | 0.15 | 0.135 | A |
| 23.059 | -5.623 | -28.910 | -0.33 | 0.01 | 0.008 | A |
| 23.461 | -4.432 | -29.485 | -0.29 | 0.02 | 0.045 | A |
| 23.974 | -4.777 | -30.703 | -0.33 | 0.05 | 0.114 | A |
| 23.912 | -6.044 | -30.894 | -0.85 | -0.18 | -0.303 | OA |
| 22.423 | -5.826 | -27.558 | -0.38 | 0.03 | 0.033 | C |
| 24.006 | -0.425 | -29.633 | -0.25 | -0.02 | -0.078 | Cl |
| 25.086 | -4.548 | -33.087 | -0.66 | 0.00 | -0.078 | Cl |
| 23.121 | -8.118 | -29.660 | -0.22 | 0.50 | 0.222 | C |
| 22.288 | -8.673 | -30.383 | -0.31 | -1.28 | -0.284 | OA |
| 23.852 | -9.008 | -28.624 | -0.34 | 0.14 | 0.093 | C |
| 25.211 | -8.965 | -28.480 | -0.36 | 0.06 | 0.073 | C |
| 25.900 | -9.842 | -27.470 | -0.48 | 0.03 | 0.051 | C |
| 25.003 | -10.122 | -26.276 | -0.52 | 0.01 | 0.015 | C |
| 23.741 | -10.812 | -26.730 | -0.42 | 0.06 | 0.07 | C |
| 23.074 | -9.991 | -27.783 | -0.40 | 0.32 | 0.178 | C |
| 21.893 | -10.224 | -28.013 | -0.33 | -1.03 | -0.291 | OA |
| 26.081 | -8.127 | -29.255 | -0.34 | -0.18 | -0.363 | OA |
| 25.635 | -7.918 | -30.109 | -0.46 | 0.04 | 0.217 | HD |
| 22.848 | -2.875 | -27.729 | -0.13 | -0.14 | -0.327 | OA |
| 21.571 | -2.241 | -27.636 | -0.25 | 0.07 | 0.181 | C |
| 24.936 | -1.412 | -31.935 | -0.16 | -0.07 | -0.326 | OA |
| 26.328 | -1.325 | -32.170 | -0.42 | 0.03 | 0.181 | C |

Estimated Free Energy of Binding = -9.45 kcal/mol [=(1)+(2)+(3)-(4)]

(1) Final Intermolecular Energy = -10.94 kcal/mol

vdW + Hbond + desolv Energy = -9.67 kcal/mol

Electrostatic Energy = -1.27 kcal/mol

(2) Final Total Internal Energy = -1.09 kcal/mol

(3) Torsional Free Energy = +1.49 kcal/mol

(4) Unbound System's Energy [=(2)] = -1.09 kcal/mol

S.I. 22 - Docking output: P3 compound lowest-energy pose

| **Coordinates** | | | **Energies** | | **Partial charge** | **Atom type** |
| --- | --- | --- | --- | --- | --- | --- |
| x | y | z | vdW+H-bond | Electrostatic |  |  |
| 22.177 | -4.135 | -28.372 | -0.28 | 0.06 | 0.102 | A |
| 22.441 | -3.036 | -29.238 | -0.25 | 0.05 | 0.116 | A |
| 23.175 | -3.262 | -30.421 | -0.24 | 0.04 | 0.102 | A |
| 23.640 | -4.535 | -30.733 | -0.30 | 0.02 | 0.049 | A |
| 22.644 | -5.371 | -28.756 | -0.31 | 0.04 | 0.049 | A |
| 23.377 | -5.601 | -29.899 | -0.30 | 0.07 | 0.099 | A |
| 23.872 | -6.874 | -30.209 | -0.59 | -0.27 | -0.322 | OA |
| 25.261 | -7.134 | -29.692 | -0.24 | 0.09 | 0.217 | C |
| 25.518 | -8.356 | -28.678 | -0.33 | 0.14 | 0.208 | C |
| 26.509 | -9.044 | -28.928 | -0.38 | -0.18 | -0.288 | OA |
| 24.533 | -8.870 | -27.608 | -0.36 | 0.08 | 0.090 | C |
| 23.155 | -9.308 | -27.988 | -0.36 | 0.35 | 0.177 | C |
| 22.174 | -9.766 | -26.955 | -0.44 | 0.13 | 0.070 | C |
| 22.808 | -9.364 | -29.165 | -0.34 | -1.03 | -0.291 | OA |
| 22.524 | -9.158 | -25.615 | -0.54 | 0.01 | 0.015 | C |
| 23.965 | -9.485 | -25.254 | -0.51 | 0.03 | 0.051 | C |
| 24.918 | -8.978 | -26.306 | -0.42 | 0.05 | 0.073 | C |
| 26.224 | -8.663 | -25.852 | -0.18 | -0.27 | -0.363 | OA |
| 26.673 | -9.506 | -25.566 | -0.41 | 0.12 | 0.217 | HD |
| 23.499 | -2.214 | -31.345 | -0.18 | -0.10 | -0.328 | OA |
| 24.522 | -2.657 | -32.230 | -0.35 | 0.04 | 0.181 | C |
| 21.483 | -4.283 | -27.033 | -0.28 | -0.16 | -0.328 | OA |
| 22.036 | -4.994 | -25.940 | -0.30 | 0.08 | 0.181 | C |
| 21.992 | -1.588 | -28.971 | -0.12 | 0.15 | 0.422 | C |
| 21.166 | -1.182 | -29.995 | -0.15 | -0.06 | -0.166 | F |
| 21.324 | -1.390 | -27.790 | -0.08 | -0.06 | -0.166 | F |
| 23.096 | -0.772 | -28.972 | -0.04 | -0.05 | -0.166 | F |

Estimated Free Energy of Binding = -6.51 kcal/mol [=(1)+(2)+(3)-(4)]

(1) Final Intermolecular Energy = -8.90 kcal/mol

vdW + Hbond + desolv Energy = -8.29 kcal/mol

Electrostatic Energy = -0.60 kcal/mol

(2) Final Total Internal Energy = -0.86 kcal/mol

(3) Torsional Free Energy = +2.39 kcal/mol

(4) Unbound System's Energy [=(2)] = -0.86 kcal/mol

S.I. 23 - Docking output: P4 compound lowest-energy pose

| **Coordinates** | | | **Energies** | | **Partial charge** | **Atom type** |
| --- | --- | --- | --- | --- | --- | --- |
| x | y | z | vdW+H-bond | Electrostatic |  |  |
| 22.364 | -2.772 | -29.636 | -0.26 | 0.04 | 0.102 | A |
| 22.822 | -2.091 | -30.804 | -0.24 | 0.04 | 0.118 | A |
| 23.865 | -2.631 | -31.602 | -0.27 | 0.04 | 0.129 | A |
| 24.005 | -4.507 | -30.121 | -0.28 | 0.06 | 0.126 | A |
| 24.456 | -3.849 | -31.240 | -0.30 | 0.05 | 0.184 | A |
| 22.984 | -3.979 | -29.341 | -0.28 | 0.03 | 0.051 | A |
| 25.477 | -4.485 | -31.912 | -0.15 | -0.01 | -0.200 | F |
| 24.635 | -5.681 | -29.830 | -0.26 | -0.15 | -0.320 | OA |
| 24.039 | -6.789 | -30.526 | -0.22 | 0.15 | 0.218 | C |
| 23.186 | -7.698 | -29.575 | -0.33 | 0.39 | 0.208 | C |
| 21.950 | -7.598 | -29.631 | -0.24 | -1.23 | -0.288 | OA |
| 23.867 | -8.656 | -28.570 | -0.37 | 0.13 | 0.090 | C |
| 25.210 | -8.686 | -28.384 | -0.42 | 0.06 | 0.073 | C |
| 25.846 | -9.645 | -27.402 | -0.47 | 0.04 | 0.051 | C |
| 24.891 | -9.951 | -26.255 | -0.51 | 0.01 | 0.015 | C |
| 23.608 | -10.547 | -26.795 | -0.46 | 0.07 | 0.070 | C |
| 23.019 | -9.640 | -27.823 | -0.38 | 0.35 | 0.177 | C |
| 21.857 | -9.851 | -28.182 | -0.07 | -1.41 | -0.291 | OA |
| 26.084 | -7.863 | -29.084 | -0.34 | -0.20 | -0.363 | OA |
| 25.922 | -7.981 | -30.055 | -0.42 | 0.05 | 0.217 | HD |
| 21.252 | -2.416 | -28.560 | -0.22 | -0.13 | -0.328 | OA |
| 21.262 | -1.236 | -27.706 | -0.24 | 0.06 | 0.181 | C |
| 24.297 | -1.867 | -32.805 | -0.19 | -0.07 | -0.326 | OA |
| 25.661 | -1.849 | -33.203 | -0.40 | 0.03 | 0.181 | C |
| 22.223 | -0.732 | -31.271 | -0.15 | 0.13 | 0.422 | C |
| 23.103 | 0.280 | -30.948 | -0.04 | -0.04 | -0.166 | F |
| 21.934 | -0.598 | -32.604 | -0.14 | -0.05 | -0.166 | F |
| 21.050 | -0.504 | -30.584 | -0.14 | -0.06 | -0.166 | F |

Estimated Free Energy of Binding = -7.05 kcal/mol [=(1)+(2)+(3)-(4)]

(1) Final Intermolecular Energy = -9.44 kcal/mol

vdW + Hbond + desolv Energy = -7.80 kcal/mol

Electrostatic Energy = -1.64 kcal/mol

(2) Final Total Internal Energy = -0.57 kcal/mol

(3) Torsional Free Energy = +2.39 kcal/mol

(4) Unbound System's Energy [=(2)] = -0.57 kcal/mol

S.I. 24 - Docking output: P5 compound lowest-energy pose

| **Coordinates** | | | **Energies** | | **Partial charge** | **Atom type** |
| --- | --- | --- | --- | --- | --- | --- |
| x | y | z | vdW+H-bond | Electrostatic |  |  |
| 24.004 | -6.521 | -30.909 | -0.91 | -0.21 | -0.323 | OA |
| 24.147 | -5.161 | -31.050 | -0.38 | 0.04 | 0.094 | A |
| 24.674 | -4.586 | -32.193 | -0.50 | 0.00 | 0.020 | A |
| 24.793 | -3.211 | -32.298 | -0.47 | 0.00 | -0.016 | A |
| 24.378 | -2.376 | -31.247 | -0.32 | 0.02 | 0.064 | A |
| 25.387 | -2.696 | -33.597 | -0.51 | 0.01 | 0.031 | C |
| 23.841 | -2.974 | -30.091 | -0.30 | -0.01 | -0.016 | A |
| 23.732 | -4.352 | -30.010 | -0.34 | 0.01 | 0.020 | A |
| 23.346 | -2.229 | -28.872 | -0.27 | 0.01 | 0.031 | C |
| 24.535 | -0.828 | -31.418 | -0.11 | 0.10 | 0.418 | C |
| 24.723 | -0.351 | -32.692 | -0.07 | -0.03 | -0.166 | F |
| 23.411 | -0.199 | -30.913 | -0.04 | -0.04 | -0.166 | F |
| 25.609 | -0.393 | -30.660 | -0.10 | -0.04 | -0.166 | F |
| 23.124 | -6.943 | -29.791 | -0.35 | 0.31 | 0.217 | C |
| 23.110 | -8.501 | -29.497 | -0.21 | 0.52 | 0.208 | C |
| 22.381 | -9.218 | -30.193 | -0.31 | -1.36 | -0.288 | OA |
| 23.964 | -9.140 | -28.360 | -0.35 | 0.12 | 0.090 | C |
| 25.314 | -8.974 | -28.288 | -0.41 | 0.06 | 0.073 | C |
| 26.129 | -9.607 | -27.179 | -0.45 | 0.03 | 0.051 | C |
| 25.306 | -9.762 | -25.912 | -0.46 | 0.01 | 0.015 | C |
| 24.081 | -10.604 | -26.191 | -0.42 | 0.05 | 0.070 | C |
| 23.300 | -10.005 | -27.316 | -0.38 | 0.24 | 0.177 | C |
| 22.111 | -10.297 | -27.398 | -0.41 | -0.63 | -0.291 | OA |
| 26.050 | -8.242 | -29.225 | -0.33 | -0.19 | -0.363 | OA |
| 25.440 | -7.958 | -29.953 | -0.41 | 0.06 | 0.217 | HD |

Estimated Free Energy of Binding = -7.94 kcal/mol [=(1)+(2)+(3)-(4)]

(1) Final Intermolecular Energy = -9.73 kcal/mol

vdW + Hbond + desolv Energy = -8.81 kcal/mol

Electrostatic Energy = -0.93 kcal/mol

(2) Final Total Internal Energy = -1.02 kcal/mol

(3) Torsional Free Energy = +1.79 kcal/mol

(4) Unbound System's Energy [=(2)] = -1.02 kcal/mol

S.I. 25 - Docking output: P6 compound lowest-energy pose

| **Coordinates** | | | **Energies** | | **Partial charge** | **Atom type** |
| --- | --- | --- | --- | --- | --- | --- |
| x | y | z | vdW+H-bond | Electrostatic |  |  |
| 23.851 | -4.925 | -30.625 | -0.34 | 0.05 | 0.099 | A |
| 24.810 | -4.430 | -31.505 | -0.44 | 0.01 | 0.049 | A |
| 25.113 | -3.068 | -31.562 | -0.35 | 0.02 | 0.108 | A |
| 24.427 | -2.189 | -30.731 | -0.27 | 0.03 | 0.102 | A |
| 23.457 | -2.672 | -29.852 | -0.24 | 0.04 | 0.108 | A |
| 24.767 | -0.497 | -30.795 | -0.34 | -0.02 | -0.078 | Cl |
| 23.180 | -4.034 | -29.793 | -0.28 | 0.02 | 0.049 | A |
| 26.100 | -2.583 | -32.465 | -0.35 | -0.06 | -0.327 | OA |
| 25.450 | -2.220 | -33.697 | -0.37 | 0.04 | 0.181 | C |
| 22.752 | -1.771 | -29.038 | -0.11 | -0.12 | -0.327 | OA |
| 21.387 | -1.594 | -29.457 | -0.31 | 0.06 | 0.181 | C |
| 24.340 | -7.172 | -29.688 | -0.27 | 0.16 | 0.217 | C |
| 23.555 | -8.094 | -28.724 | -0.28 | 0.33 | 0.208 | C |
| 22.357 | -8.273 | -28.987 | -0.17 | -1.30 | -0.288 | OA |
| 24.169 | -8.826 | -27.533 | -0.35 | 0.09 | 0.090 | C |
| 25.364 | -9.411 | -27.688 | -0.42 | 0.05 | 0.073 | C |
| 26.043 | -10.143 | -26.557 | -0.48 | 0.03 | 0.051 | C |
| 25.013 | -10.726 | -25.595 | -0.56 | 0.01 | 0.015 | C |
| 24.120 | -9.626 | -25.051 | -0.47 | 0.04 | 0.070 | C |
| 23.507 | -8.868 | -26.190 | -0.34 | 0.16 | 0.177 | C |
| 22.461 | -8.266 | -25.985 | -0.82 | -0.32 | -0.291 | OA |
| 26.040 | -9.300 | -28.882 | -0.26 | -0.23 | -0.363 | OA |
| 25.394 | -9.340 | -29.602 | -0.43 | 0.11 | 0.217 | HD |

Estimated Free Energy of Binding = -8.07 kcal/mol [=(1)+(2)+(3)-(4)]

(1) Final Intermolecular Energy = -10.16 kcal/mol

vdW + Hbond + desolv Energy = -9.12 kcal/mol

Electrostatic Energy = -1.05 kcal/mol

(2) Final Total Internal Energy = -0.48 kcal/mol

(3) Torsional Free Energy = +2.09 kcal/mol

(4) Unbound System's Energy [=(2)] = -0.48 kcal/mol

S.I. 26 - Docking output: P7 compound lowest-energy pose

| **Coordinates** | | | **Energies** | | **Partial charge** | **Atom type** |
| --- | --- | --- | --- | --- | --- | --- |
| x | y | z | vdW+H-bond | Electrostatic |  |  |
| 23.521 | -5.339 | -29.335 | -0.19 | -0.22 | -0.322 | OA |
| 23.529 | -4.189 | -30.122 | -0.27 | 0.05 | 0.109 | A |
| 24.443 | -4.012 | -31.155 | -0.39 | 0.01 | 0.050 | A |
| 24.385 | -2.858 | -31.922 | -0.34 | 0.03 | 0.109 | A |
| 23.422 | -1.896 | -31.641 | -0.26 | 0.03 | 0.103 | A |
| 22.498 | -2.050 | -30.607 | -0.24 | 0.04 | 0.119 | A |
| 23.367 | -0.438 | -32.624 | -0.40 | -0.02 | -0.078 | Cl |
| 22.562 | -3.234 | -29.839 | -0.22 | 0.04 | 0.103 | A |
| 21.523 | -3.689 | -28.493 | -0.51 | -0.04 | -0.078 | Cl |
| 25.289 | -2.642 | -32.993 | -0.27 | -0.07 | -0.327 | OA |
| 26.476 | -1.992 | -32.535 | -0.41 | 0.03 | 0.181 | C |
| 21.562 | -0.900 | -30.489 | -0.22 | -0.11 | -0.326 | OA |
| 20.993 | -0.491 | -29.233 | -0.26 | 0.06 | 0.181 | C |
| 24.060 | -6.530 | -30.022 | -0.35 | 0.16 | 0.217 | C |
| 23.384 | -7.891 | -29.578 | -0.22 | 0.36 | 0.208 | C |
| 22.317 | -8.214 | -30.118 | -0.32 | -1.22 | -0.288 | OA |
| 24.082 | -8.883 | -28.583 | -0.33 | 0.11 | 0.090 | C |
| 25.436 | -8.948 | -28.442 | -0.39 | 0.05 | 0.073 | C |
| 26.080 | -9.919 | -27.474 | -0.48 | 0.03 | 0.051 | C |
| 25.170 | -10.194 | -26.289 | -0.53 | 0.01 | 0.015 | C |
| 23.854 | -10.762 | -26.773 | -0.45 | 0.06 | 0.070 | C |
| 23.242 | -9.838 | -27.776 | -0.38 | 0.30 | 0.177 | C |
| 22.043 | -9.965 | -28.015 | -0.33 | -1.06 | -0.291 | OA |
| 26.329 | -8.160 | -29.176 | -0.30 | -0.19 | -0.363 | OA |
| 26.174 | -8.322 | -30.140 | -0.46 | 0.04 | 0.217 | HD |

Estimated Free Energy of Binding = -7.93 kcal/mol [=(1)+(2)+(3)-(4)]

(1) Final Intermolecular Energy = -10.02 kcal/mol

vdW + Hbond + desolv Energy = -8.51 kcal/mol

Electrostatic Energy = -1.50 kcal/mol

(2) Final Total Internal Energy = -0.59 kcal/mol

(3) Torsional Free Energy = +2.09 kcal/mol

(4) Unbound System's Energy [=(2)] = -0.59 kcal/mol

S.I. 27 - Docking output: P8 compound lowest-energy pose

| **Coordinates** | | | **Energies** | | **Partial charge** | **Atom type** |
| --- | --- | --- | --- | --- | --- | --- |
| x | y | z | vdW+H-bond | Electrostatic |  |  |
| 23.143 | -8.117 | -29.693 | -0.24 | 0.46 | 0.208 | C |
| 22.327 | -8.683 | -30.432 | -0.38 | -1.22 | -0.288 | OA |
| 23.909 | -8.946 | -28.617 | -0.33 | 0.13 | 0.090 | C |
| 23.155 | -9.909 | -27.732 | -0.39 | 0.30 | 0.177 | C |
| 23.850 | -10.689 | -26.663 | -0.45 | 0.06 | 0.070 | C |
| 21.959 | -10.126 | -27.906 | -0.36 | -0.97 | -0.291 | OA |
| 25.095 | -9.954 | -26.219 | -0.51 | 0.01 | 0.015 | C |
| 25.987 | -9.675 | -27.416 | -0.47 | 0.03 | 0.051 | C |
| 25.259 | -8.860 | -28.465 | -0.40 | 0.06 | 0.073 | C |
| 26.075 | -8.048 | -29.259 | -0.35 | -0.18 | -0.363 | OA |
| 25.563 | -7.771 | -30.060 | -0.40 | 0.05 | 0.217 | HD |
| 23.365 | -6.559 | -29.876 | -0.33 | 0.23 | 0.217 | C |
| 24.103 | -6.177 | -31.096 | -0.83 | -0.17 | -0.323 | OA |
| 24.079 | -4.794 | -31.379 | -0.36 | 0.03 | 0.094 | A |
| 23.480 | -3.870 | -30.526 | -0.31 | 0.01 | 0.020 | A |
| 23.482 | -2.518 | -30.840 | -0.32 | -0.01 | -0.024 | A |
| 22.828 | -1.532 | -29.910 | -0.29 | 0.01 | 0.031 | C |
| 24.087 | -2.081 | -32.013 | -0.34 | -0.01 | -0.030 | A |
| 24.090 | -0.619 | -32.357 | -0.30 | 0.01 | 0.030 | C |
| 24.689 | -2.998 | -32.866 | -0.45 | -0.01 | -0.024 | A |
| 25.348 | -2.536 | -34.136 | -0.35 | 0.01 | 0.031 | C |
| 24.685 | -4.350 | -32.548 | -0.52 | 0.00 | 0.020 | A |

Estimated Free Energy of Binding = -8.38 kcal/mol [=(1)+(2)+(3)-(4)]

(1) Final Intermolecular Energy = -9.87 kcal/mol

vdW + Hbond + desolv Energy = -8.71 kcal/mol

Electrostatic Energy = -1.16 kcal/mol

(2) Final Total Internal Energy = -0.78 kcal/mol

(3) Torsional Free Energy = +1.49 kcal/mol

(4) Unbound System's Energy [=(2)] = -0.78 kcal/mol

S.I. 28 - Docking output: P9 compound lowest-energy pose

| **Coordinates** | | | **Energies** | | **Partial charge** | **Atom type** |
| --- | --- | --- | --- | --- | --- | --- |
| x | y | z | vdW+H-bond | Electrostatic |  |  |
| 24.409 | -7.343 | -29.377 | -0.28 | 0.17 | 0.217 | C |
| 23.659 | -6.716 | -30.489 | -0.87 | -0.29 | -0.322 | OA |
| 23.819 | -5.314 | -30.570 | -0.34 | 0.06 | 0.107 | A |
| 24.686 | -4.723 | -31.487 | -0.45 | 0.01 | 0.037 | A |
| 24.816 | -3.341 | -31.542 | -0.38 | 0.02 | 0.071 | A |
| 25.918 | -2.611 | -32.704 | -0.73 | -0.01 | -0.081 | Cl |
| 24.077 | -2.543 | -30.677 | -0.28 | 0.02 | 0.080 | A |
| 24.238 | -0.791 | -30.742 | -0.32 | -0.02 | -0.081 | Cl |
| 23.208 | -3.128 | -29.763 | -0.27 | 0.03 | 0.082 | A |
| 22.268 | -2.117 | -28.669 | -0.39 | -0.03 | -0.081 | Cl |
| 23.079 | -4.511 | -29.711 | -0.25 | 0.05 | 0.091 | A |
| 21.978 | -5.254 | -28.555 | -0.54 | -0.07 | -0.079 | Cl |
| 23.541 | -8.279 | -28.439 | -0.27 | 0.34 | 0.208 | C |
| 22.328 | -8.370 | -28.665 | -0.17 | -1.24 | -0.288 | OA |
| 24.165 | -9.069 | -27.249 | -0.37 | 0.09 | 0.090 | C |
| 23.568 | -9.123 | -26.026 | -0.45 | 0.06 | 0.073 | C |
| 24.189 | -9.892 | -24.879 | -0.51 | 0.03 | 0.051 | C |
| 25.013 | -11.064 | -25.384 | -0.54 | 0.01 | 0.015 | C |
| 26.098 | -10.570 | -26.315 | -0.47 | 0.03 | 0.070 | C |
| 25.489 | -9.772 | -27.424 | -0.35 | 0.13 | 0.177 | C |
| 26.138 | -9.655 | -28.459 | -0.31 | -0.20 | -0.291 | OA |
| 22.369 | -8.470 | -25.725 | -0.73 | -0.37 | -0.363 | OA |
| 22.042 | -8.016 | -26.543 | 0.19 | 0.31 | 0.217 | HD |

Estimated Free Energy of Binding = -8.47 kcal/mol [=(1)+(2)+(3)-(4)]

(1) Final Intermolecular Energy = -9.96 kcal/mol

vdW + Hbond + desolv Energy = -9.08 kcal/mol

Electrostatic Energy = -0.88 kcal/mol

(2) Final Total Internal Energy = -1.15 kcal/mol

(3) Torsional Free Energy = +1.49 kcal/mol

(4) Unbound System's Energy [=(2)] = -1.15 kcal/mol

S.I. 29 - Docking output: P10 compound lowest-energy pose

| **Coordinates** | | | **Energies** | | **Partial charge** | **Atom type** |
| --- | --- | --- | --- | --- | --- | --- |
| x | y | z | vdW+H-bond | Electrostatic |  |  |
| 23.859 | -6.318 | -29.694 | -0.32 | 0.18 | 0.219 | C |
| 23.788 | -5.899 | -31.097 | -0.81 | -0.19 | -0.306 | OA |
| 24.071 | -4.519 | -31.289 | -0.31 | 0.06 | 0.185 | A |
| 25.023 | -4.099 | -32.125 | -0.39 | -0.03 | -0.181 | NA |
| 25.260 | -2.791 | -32.283 | -0.29 | 0.06 | 0.274 | A |
| 24.553 | -1.883 | -31.611 | -0.23 | -0.05 | -0.181 | NA |
| 23.592 | -2.237 | -30.765 | -0.21 | 0.06 | 0.185 | A |
| 23.324 | -3.586 | -30.582 | -0.24 | 0.03 | 0.089 | A |
| 26.261 | -2.332 | -33.165 | -0.35 | -0.05 | -0.297 | OA |
| 25.704 | -1.460 | -34.153 | -0.37 | 0.03 | 0.185 | C |
| 22.853 | -1.260 | -30.061 | -0.13 | -0.10 | -0.312 | OA |
| 21.456 | -1.574 | -30.087 | -0.31 | 0.06 | 0.183 | C |
| 23.252 | -7.771 | -29.449 | -0.32 | 0.38 | 0.208 | C |
| 22.217 | -8.076 | -30.054 | -0.32 | -1.26 | -0.288 | OA |
| 23.935 | -8.808 | -28.495 | -0.36 | 0.12 | 0.090 | C |
| 25.291 | -8.940 | -28.392 | -0.40 | 0.06 | 0.073 | C |
| 25.912 | -9.960 | -27.461 | -0.47 | 0.03 | 0.051 | C |
| 25.024 | -10.222 | -26.259 | -0.53 | 0.01 | 0.015 | C |
| 23.672 | -10.714 | -26.718 | -0.43 | 0.06 | 0.070 | C |
| 23.075 | -9.734 | -27.676 | -0.39 | 0.32 | 0.177 | C |
| 21.860 | -9.762 | -27.828 | -0.27 | -1.12 | -0.291 | OA |
| 26.213 | -8.180 | -29.126 | -0.33 | -0.20 | -0.363 | OA |
| 25.944 | -8.191 | -30.079 | -0.46 | 0.04 | 0.217 | HD |

Estimated Free Energy of Binding = -7.63 kcal/mol [=(1)+(2)+(3)-(4)]

(1) Final Intermolecular Energy = -9.72 kcal/mol

vdW + Hbond + desolv Energy = -8.25 kcal/mol

Electrostatic Energy = -1.47 kcal/mol

(2) Final Total Internal Energy = -0.40 kcal/mol

(3) Torsional Free Energy = +2.09 kcal/mol

(4) Unbound System's Energy [=(2)] = -0.40 kcal/mol

S.I. 30 - Docking output: P11 compound lowest-energy pose

| **Coordinates** | | | **Energies** | | **Partial charge** | **Atom type** |
| --- | --- | --- | --- | --- | --- | --- |
| x | y | z | vdW+H-bond | Electrostatic |  |  |
| 23.286 | -5.560 | -30.810 | -0.62 | -0.21 | -0.304 | OA |
| 23.659 | -6.236 | -29.573 | -0.31 | 0.19 | 0.219 | C |
| 23.258 | -7.766 | -29.538 | -0.31 | 0.38 | 0.208 | C |
| 22.306 | -8.139 | -30.243 | -0.43 | -1.13 | -0.288 | OA |
| 23.946 | -8.786 | -28.548 | -0.36 | 0.12 | 0.090 | C |
| 23.079 | -9.696 | -27.707 | -0.38 | 0.32 | 0.177 | C |
| 21.859 | -9.709 | -27.848 | -0.24 | -1.15 | -0.291 | OA |
| 23.671 | -10.656 | -26.729 | -0.44 | 0.06 | 0.070 | C |
| 25.021 | -10.154 | -26.275 | -0.52 | 0.01 | 0.015 | C |
| 25.915 | -9.919 | -27.476 | -0.48 | 0.03 | 0.051 | C |
| 25.302 | -8.920 | -28.435 | -0.40 | 0.06 | 0.073 | C |
| 26.234 | -8.182 | -29.184 | -0.34 | -0.19 | -0.363 | OA |
| 25.913 | -8.125 | -30.120 | -0.45 | 0.04 | 0.217 | HD |
| 23.936 | -4.311 | -30.930 | -0.25 | 0.08 | 0.213 | A |
| 23.680 | -3.308 | -30.087 | -0.23 | -0.07 | -0.179 | NA |
| 24.283 | -2.123 | -30.219 | -0.15 | 0.08 | 0.274 | A |
| 25.167 | -1.922 | -31.199 | -0.26 | -0.04 | -0.179 | NA |
| 25.470 | -2.884 | -32.070 | -0.34 | 0.04 | 0.213 | A |
| 24.849 | -4.119 | -31.956 | -0.34 | 0.04 | 0.222 | A |
| 25.128 | -5.121 | -32.832 | -0.13 | 0.02 | -0.197 | F |
| 23.990 | -1.047 | -29.308 | -0.12 | -0.10 | -0.297 | OA |
| 22.599 | -1.042 | -28.958 | -0.17 | 0.06 | 0.185 | C |
| 26.406 | -2.656 | -33.101 | -0.32 | -0.06 | -0.310 | OA |
| 25.913 | -1.673 | -34.013 | -0.35 | 0.03 | 0.183 | C |

Estimated Free Energy of Binding = -7.24 kcal/mol [=(1)+(2)+(3)-(4)]

(1) Final Intermolecular Energy = -9.33 kcal/mol

vdW + Hbond + desolv Energy = -7.94 kcal/mol

Electrostatic Energy = -1.39 kcal/mol

(2) Final Total Internal Energy = -0.46 kcal/mol

(3) Torsional Free Energy = +2.09 kcal/mol

(4) Unbound System's Energy [=(2)] = -0.46 kcal/mol

S.I. 31 - Docking output: P12 compound lowest-energy pose

| **Coordinates** | | | **Energies** | | **Partial charge** | **Atom type** |
| --- | --- | --- | --- | --- | --- | --- |
| x | y | z | vdW+H-bond | Electrostatic |  |  |
| 24.365 | -5.726 | -30.607 | -0.46 | -0.13 | -0.305 | OA |
| 24.157 | -6.142 | -29.201 | -0.28 | 0.15 | 0.219 | C |
| 22.901 | -7.066 | -28.946 | -0.29 | 0.36 | 0.208 | C |
| 21.793 | -6.682 | -29.344 | -0.33 | -0.65 | -0.288 | OA |
| 22.989 | -8.314 | -28.042 | -0.35 | 0.19 | 0.090 | C |
| 22.729 | -9.561 | -28.520 | -0.40 | 0.23 | 0.073 | C |
| 22.796 | -10.769 | -27.627 | -0.52 | 0.08 | 0.051 | C |
| 23.786 | -10.569 | -26.493 | -0.47 | 0.01 | 0.015 | C |
| 23.407 | -9.350 | -25.683 | -0.49 | 0.05 | 0.070 | C |
| 23.315 | -8.161 | -26.579 | -0.28 | 0.19 | 0.177 | C |
| 23.477 | -7.054 | -26.071 | -0.53 | -0.22 | -0.291 | OA |
| 22.359 | -9.819 | -29.845 | -0.23 | -1.70 | -0.363 | OA |
| 23.163 | -9.684 | -30.424 | -0.43 | 0.42 | 0.217 | HD |
| 23.883 | -4.399 | -30.825 | -0.26 | 0.08 | 0.196 | A |
| 23.062 | -3.824 | -29.957 | -0.23 | -0.08 | -0.180 | NA |
| 22.585 | -2.603 | -30.157 | -0.15 | 0.10 | 0.274 | A |
| 22.927 | -1.895 | -31.234 | -0.22 | -0.05 | -0.180 | NA |
| 23.759 | -2.396 | -32.142 | -0.21 | 0.05 | 0.196 | A |
| 24.258 | -3.683 | -31.958 | -0.35 | 0.03 | 0.139 | A |
| 25.350 | -4.391 | -33.143 | -0.77 | 0.00 | -0.075 | Cl |
| 21.679 | -2.101 | -29.223 | -0.23 | -0.11 | -0.297 | OA |
| 21.524 | -3.008 | -28.160 | -0.28 | 0.08 | 0.185 | C |
| 24.126 | -1.639 | -33.284 | -0.20 | -0.07 | -0.311 | OA |
| 25.404 | -1.020 | -33.085 | -0.33 | 0.03 | 0.183 | C |

Estimated Free Energy of Binding = -7.17 kcal/mol [=(1)+(2)+(3)-(4)]

(1) Final Intermolecular Energy = -9.25 kcal/mol

vdW + Hbond + desolv Energy = -8.29 kcal/mol

Electrostatic Energy = -0.97 kcal/mol

(2) Final Total Internal Energy = -0.72 kcal/mol

(3) Torsional Free Energy = +2.09 kcal/mol

(4) Unbound System's Energy [=(2)] = -0.72 kcal/mol

S.I. 32 - Docking output: P13 compound lowest-energy pose

| **Coordinates** | | | **Energies** | | **Partial charge** | **Atom type** |
| --- | --- | --- | --- | --- | --- | --- |
| x | y | z | vdW+H-bond | Electrostatic |  |  |
| 23.734 | -8.077 | -28.653 | -0.28 | 0.30 | 0.208 | C |
| 22.550 | -8.177 | -28.987 | -0.25 | -1.05 | -0.288 | OA |
| 24.227 | -8.836 | -27.432 | -0.36 | 0.09 | 0.090 | C |
| 25.493 | -9.597 | -27.561 | -0.35 | 0.13 | 0.177 | C |
| 26.047 | -10.397 | -26.427 | -0.47 | 0.04 | 0.070 | C |
| 26.125 | -9.557 | -28.611 | -0.32 | -0.19 | -0.291 | OA |
| 24.914 | -10.812 | -25.509 | -0.54 | 0.01 | 0.015 | C |
| 24.123 | -9.588 | -25.063 | -0.49 | 0.03 | 0.051 | C |
| 23.586 | -8.826 | -26.247 | -0.42 | 0.07 | 0.073 | C |
| 22.433 | -8.115 | -26.039 | -0.78 | -0.40 | -0.363 | OA |
| 22.201 | -7.670 | -26.874 | 0.18 | 0.32 | 0.217 | HD |
| 24.595 | -7.100 | -29.432 | -0.31 | 0.15 | 0.219 | C |
| 23.921 | -6.739 | -30.645 | -0.89 | -0.23 | -0.306 | OA |
| 23.921 | -5.330 | -30.792 | -0.30 | 0.09 | 0.183 | A |
| 23.176 | -4.571 | -29.993 | -0.23 | -0.11 | -0.193 | NA |
| 23.152 | -3.252 | -30.116 | -0.18 | 0.09 | 0.227 | A |
| 23.881 | -2.642 | -31.045 | -0.22 | -0.06 | -0.205 | NA |
| 22.136 | -2.304 | -29.036 | -0.46 | -0.02 | -0.041 | Cl |
| 24.649 | -3.341 | -31.872 | -0.35 | 0.03 | 0.144 | A |
| 24.693 | -4.724 | -31.771 | -0.44 | 0.01 | 0.075 | A |
| 25.607 | -2.516 | -33.097 | -0.70 | -0.01 | -0.061 | Cl |
| 23.734 | -8.077 | -28.653 | -0.28 | 0.30 | 0.208 | C |

Estimated Free Energy of Binding = -7.37 kcal/mol [=(1)+(2)+(3)-(4)]

(1) Final Intermolecular Energy = -8.86 kcal/mol

vdW + Hbond + desolv Energy = -8.15 kcal/mol

Electrostatic Energy = -0.71 kcal/mol

(2) Final Total Internal Energy = -1.01 kcal/mol

(3) Torsional Free Energy = +1.49 kcal/mol

(4) Unbound System's Energy [=(2)] = -1.01 kcal/mol

S.I. 33 - Docking output: P14 compound lowest-energy pose

| **Coordinates** | | | **Energies** | | **Partial charge** | **Atom type** |
| --- | --- | --- | --- | --- | --- | --- |
| x | y | z | vdW+H-bond | Electrostatic |  |  |
| 23.703 | -7.710 | -29.397 | -0.26 | 0.28 | 0.208 | C |
| 22.535 | -7.711 | -29.789 | -0.43 | -0.86 | -0.288 | OA |
| 24.645 | -6.713 | -30.009 | -0.30 | 0.11 | 0.219 | C |
| 23.943 | -6.037 | -31.084 | -0.85 | -0.17 | -0.305 | OA |
| 23.762 | -4.617 | -30.899 | -0.23 | 0.08 | 0.194 | A |
| 23.024 | -4.109 | -29.916 | -0.22 | -0.10 | -0.192 | NA |
| 22.866 | -2.796 | -29.771 | -0.19 | 0.09 | 0.227 | A |
| 21.897 | -2.193 | -28.471 | -0.44 | -0.02 | -0.041 | Cl |
| 23.452 | -1.939 | -30.600 | -0.19 | -0.06 | -0.204 | NA |
| 24.207 | -2.372 | -31.602 | -0.27 | 0.04 | 0.155 | A |
| 24.966 | -1.241 | -32.677 | -0.51 | -0.01 | -0.060 | Cl |
| 24.382 | -3.739 | -31.777 | -0.37 | 0.03 | 0.127 | A |
| 25.361 | -4.338 | -33.073 | -0.77 | -0.01 | -0.076 | Cl |
| 24.144 | -8.804 | -28.452 | -0.36 | 0.11 | 0.090 | C |
| 25.420 | -9.219 | -28.305 | -0.36 | 0.05 | 0.073 | C |
| 25.760 | -10.336 | -27.356 | -0.45 | 0.03 | 0.051 | C |
| 24.773 | -10.388 | -26.190 | -0.54 | 0.01 | 0.015 | C |
| 23.358 | -10.553 | -26.712 | -0.40 | 0.07 | 0.070 | C |
| 23.072 | -9.473 | -27.701 | -0.37 | 0.33 | 0.177 | C |
| 21.902 | -9.220 | -27.991 | 0.05 | -1.39 | -0.291 | OA |
| 26.478 | -8.667 | -28.999 | -0.28 | -0.22 | -0.363 | OA |
| 26.336 | -8.842 | -29.936 | -0.43 | 0.07 | 0.217 | HD |

Estimated Free Energy of Binding = -8.19 kcal/mol [=(1)+(2)+(3)-(4)]

(1) Final Intermolecular Energy = -9.68 kcal/mol

vdW + Hbond + desolv Energy = -8.16 kcal/mol

Electrostatic Energy = -1.52 kcal/mol

(2) Final Total Internal Energy = -0.24 kcal/mol

(3) Torsional Free Energy = +1.49 kcal/mol

(4) Unbound System's Energy [=(2)] = -0.24 kcal/mol
